# Morphometric synthesis of *Pollimyrus* (Teleostei, Mormyridae) with the description of four new species

**DOI:** 10.1111/jfb.15983

**Published:** 2024-11-24

**Authors:** Katrien Dierickx, Soleil Wamuini Lunkayilakio, Roger Bills, Emmanuel Vreven

**Affiliations:** ^1^ Department of Archaeology and Cultural History Norwegian University of Science and Technology (NTNU) Trondheim Norway; ^2^ Section of Vertebrates, Ichthyology, Royal Museum for Central Africa Tervuren Belgium; ^3^ Institut Supérieur Pédagogique (ISP) de Mbanza‐Ngungu Mbanza‐Ngungu Democratic Republic of the Congo; ^4^ South African Institute for Aquatic Biodiversity Makhanda South Africa

**Keywords:** morphology, *Pollimyrus ibalazambai* sp. nov., *Pollimyrus krameri* sp. nov., *Pollimyrus vanneeri* sp. nov., *Pollimyrus weyli* sp. nov., taxonomy

## Abstract

Mormyridae, a species‐rich family endemic to Africa, remains taxonomically understudied. This has been the case for the genus *Pollimyrus* Taverne, 1971, which hinders further understanding of the distribution, ecology, and conservation of its species. Therefore, an in‐depth morphometric comparison of all currently valid species is carried out using most of the available type specimens. Species delineations were re‐evaluated, and four species new to science described: *Pollimyrus ibalazambai* sp. nov. (the Luki River, the Democratic Republic of the Congo), *Pollimyrus krameri* sp. nov. (the Lugenda River, Mozambique), *Pollimyrus vanneeri* sp. nov. (the Kouilou‐Niari River, the Republic of the Congo), and *Pollimyrus weyli* sp. nov. (the Buzi River, Mozambique). In this study, *Pollimyrus guttatus* is confirmed to belong to *Pollimyrus*, whereas *Pollimyrus eburneensis* and *Cyphomyrus plagiostoma* seem more similar to species allocated to other genera. No or only little morphological differences were found between the type series of several species, which could indicate the need for synonymization of these species (*Pollimyrus cuandoensis* with *Pollimyrus marianne* and *Pollimyrus nigripinnis* with *Pollimyrus pulverulentus*). As such 20 species are currently morphologically identifiable in the genus *Pollimyrus*. The present study highlights the critical need for further synthetic efforts and new collecting efforts across Africa for this and other Mormyridae genera.

## 1 INTRODUCTION

The Mormyridae family, with 227 species distributed across 22 genera (Fricke et al., 2024), is a highly diverse freshwater fish endemic to Africa. These fish are well known for their unique appearance with a forked caudal fin and sometimes a protrusion on the head (elongated snout, chin swelling, or appendage), and for being weakly electric. Nevertheless, the species and generic taxonomy of this family remain understudied. This is illustrated by the regular description of new species (e.g., *Petrocephalus petersi* Kramer et al., 2012; *Pollimyrus cuandoensis* Kramer et al., 2013; *Marcusenius kaninginii* Kisekelwa et al., 2016; *Paramormyrops ntotom* Rich et al., 2017; *Marcusenius wamuinii* Decru et al., 2019; *Cyphomyrus lufirae* Mukweze Mulelenu et al., 2019; and *Marcusenius verheyenorum* Mambo et al., 2019) and even a new genus (*Cryptomyrus* Sullivan et al., 2016) in the scientific literature.

With the rise of the study of their electrical signals, that is, electric organ discharges (EODs), and genetics, it is possible to examine these fish in new, more integrative, ways. These new techniques also allowed for the discovery of new species that, based solely on the use of morphological characteristics, would have remained unnoticed (Feulner et al., 2006; Hopkins, 1999; Rich et al., 2017). However, morphological analyses continue to be an important tool to assess species diversity in fishes, especially when living fish and adequately preserved tissue material are unavailable for behavioral and molecular approaches. One of the mormyrid genera for which both its delimitation, in terms of included species, and species differentiation of included species have largely been neglected is *Pollimyrus* Taverne, 1971. This lack of knowledge hinders a better understanding of their hydro‐geographic distribution and thus also their conservation. Therefore, a synthesis of the morphological description of all species of the genus *Pollimyrus* is provided, which until now was lacking in the scientific literature.

The genus *Pollimyrus* was established by Taverne in 1971. He identified *Mormyrus isidori* Valenciennes, 1847, originally described from the “Nile” without further specifications, as its type species. Further, 24 valid species were added to the new genus by Taverne (1971a). These had previously been assigned to three other genera: *Marcusenius* Gill, 1862, *Mormyrus* Linnaeus, 1758, and *Petrocephalus* Marcusen, 1854 (Taverne, 1971a) (Table 1). The following year, Taverne (1972) rediagnosed the genus *Pollimyrus* on an osteological basis and reallocated some species to the genera *Petrocephalus* and *Heteromormyrus* Steindachner, 1866, the latter a former subgenus of *Mormyrus*, which he elevated to genus level (Table 1). During the decades following Taverne's revisions (1971a, 1971b, 1972), there have been quite some generic reassignments, with species being transferred between *Pollimyrus* and other genera and new species being described (e.g., Bigorne, 1990; Bigorne, 2003; Hopkins et al., 2007; Kramer et al., 2012; Kramer et al., 2013; Kramer & Van der Bank, 2011; Lavoué et al., 2010; Lévêque et al., 1991; Levin & Golubtsov, 2018; Rich et al., 2017; Skelton, 2019; Stiassny et al., 2021; Sullivan et al., 2022; Teugels & Hopkins, 1998) (Table 1). Nevertheless, some questions remain regarding the generic assignment of certain species, such as *Petrocephalus guttatus* Fowler, 1936, that might belong to *Pollimyrus* (Lavoué et al., 2010). To date, 18 valid species are recognized within the genus (Fricke et al., 2024; see Table 1). Taverne (1971a, 1971b, 1972) diagnosed the genus *Pollimyrus* osteologically as species with a small lateral ethmoid bone, a large and curved mesethmoid bone, six circumorbital bones with the antorbital and first infraorbital not fused, and four hypural bones (i.e., fused ventral hypurals as defined by Teugels & Hopkins, 1998). Further, with regard to the external morphology, the species of the genus have a short or elongated body. Their snout is shorter than the postorbital part of the head. The mouth is terminal, inferior, or subinferior. The caudal peduncle depth fits two to five times in the peduncle length. Meristically, they have 15–36 dorsal‐fin rays, 21–31 anal‐fin rays, 9–12 pectoral‐fin rays, 35–70 lateral‐line scales, 7–21/8–23 scales in transverse line, 8–17/6–20 scales in transverse line between the dorsal‐ and anal‐fin origins, 12–20 scales around the caudal peduncle, 5–9/6–10 bicuspid teeth on the oral jaws, and 39–45 vertebrae (Taverne, 1971a, 1971b, 1972). Besides these osteological and meristic diagnostic characteristics, these species can further be recognized morphologically based on the combination of the following, additional, qualitative characteristics: rounded head in lateral view; no mental lobe; nostrils well separated and posterior nostril close to anterior rim of eye; and dorsal‐ and anal‐fin origins at the same level (Bigorne, 2003; Hopkins et al., 2007).

**TABLE 1:** Taxonomic revision history of the nominal species and subspecies previously or currently assigned to *Pollimyrus* Taverne, 1971, according to Fricke et al. (2024) and their type localities according to the original description.

Species	Author(s)	Taverne, 1971a, 1971b	Taverne, 1972	Latest taxonomic revision	Current status	Type locality
*Mormyrus adspersus*	Günther, 1866	*Pollimyrus adspersus*	*P. adspersus*	Taverne, 1971	*P. adspersus*	West Africa
*Marcusenius aequipinnis*	Pellegrin, 1924	*Pollimyrus aequipinnis*	*P. aequipinnis*	Poll, 1976 (jun. syn.)	*P. tumifrons*	N'Gombe, Kasaï River
*Petrocephalus anterodorsalis*	David & Poll, 1937	*P. tumifrons*	*P. tumifrons*	Taverne, 1971 (jun. syn.)	*P. tumifrons*	Panga, Aruwimi River
*Marcusenius brevis*	Boulenger, 1913	*Pollimyrus brevis*	*P. brevis*	Taverne, 1971	*P. brevis*	Dungu, Uele River
*Marcusenius budgetti	Boulenger, 1904	*Pollimyrus budgetti*	*P. budgetti*	Kramer & van der Bank 2011 (jun. syn.)	*Cyphomyrus psittacus*	Assay, Niger River
*Marcusenius cabrae	Boulenger, 1900	*Pollimyrus kingsleyae*	*P. kingsleyae*	Hopkins et al., 2007 (jun. syn.)	*Paramormyrops kingsleyae*	“Kop‐Malafu,” Mayombe
*Marcusenius castelnaui*	Boulenger, 1911	*Pollimyrus castelnaui*	*P. castelnaui*	Taverne, 1971	*P. castelnaui*	Lake Ngami
*Pollimyrus cuandoensis*	Kramer et al., 2013			Kramer et al., 2013	*P. cuandoensis*	Kwando River
*Marcusenius fasciaticeps*	Boulenger, 1920	*Pollimyrus fasciaticeps*	*P. fasciaticeps*	Taverne, 1971 (subspecies)	*P. isidori fasciaticeps /P. osborni*	Kinshasa, Pool Malebo
*Marcusenius gaillardi	Pellegrin, 1909	*P. isidori*	*P. isidori*	Taverne, 1971 (jun. syn.)	*P. isidori*	Bol, Lake Chad
*Petrocephalus guttatus*	Fowler, 1936		*P. guttatus*	Lavoué, 2010	*P. guttatus*	Kribi, Kineke River
*Marcusenius hutereaui	Boulenger, 1913	*Pollimyrus hutereaui*	*Petrocephalus hutereaui*	Taverne, 1972	*P. hutereaui*	Uele River
***Mormyrus isidori* (type)**	Valenciennes, 1847	*P. isidori*	*P. isidori*	Taverne, 1971	*P. isidori*	Nile River
*Mormyrus kingsleyae	Günther, 1896	*P. kingsleyae*	*P. kingsleyae*	Hopkins et al., 2007	*Paramormyrops kingsleyae*	Old Calabar, Ogooué River
*Pollimyrus kingsleyae eburneensis*	Bigorne, 1990			Rich et al., 2017 (elevation)	*Pollimyrus eburneensis*	Agnébi basin
*Mormyrus lhuysi	Steindachner, 1870	*Pollimyrus lhuysi*	*P. lhuysi*	Bigorne, 2003 (jun. syn.)	*Brevimyrus niger*	Senegal
*P. lhuysi	Non Steindachner			Bigorne, 1990 (jun. syn.)	*P. isidori?*	Senegal
*Marcusenius macroterops	Boulenger, 1920			Sullivan et al., 2022	*Pollimyrus macroterops*	Poko, Bomokandi River
*Marcusenius macularius	Fowler, 1936	*P. lhuysi*	*P. lhuysi*	Bigorne, 2003 (jun. syn.)	*Brevimyrus niger*	Sibut
*Marcusenius maculipinnis*	Nichols & La Monte, 1934	*Pollimyrus maculipinnis*	*P. maculipinnis*	Taverne, 1971	*P. maculipinnis*	Kananga, Lulua River
*Petrocephalus marchei	Sauvage, 1879	*Pollimyrus marchii*	*P. marchei*	Hopkins et al., 2007	*Ivindomyrus marchei*	Doumé, Ogooué River
*Pollimyrus marianne*	Kramer et al., 2003			Kramer et al., 2003	*P. marianne*	Zambezi River
*Marcusenius nigricans*	Boulenger, 1906	*Pollimyrus nigricans*	*P. nigricans*	Taverne, 1971	*P. nigricans*	Mouth Katonga River
*Marcusenius nigripinnis*	Boulenger, 1899	*P. nigripinnis*	*P. nigripinnis*	Taverne, 1971	*P. nigripinnis*	Kutu, Lake Mai‐Ndombe
*Marcusenius osborni*	Nichols & Griscom, 1917	*Pollimyrus osborni*	*P. osborni*	Taverne, 1971	*P. osborni*	Uele River
*Mormyrus pauciradiatus	Steindachner, 1866	*Pollimyrus pauciradiatus*	*Heteromormyrus pauciradiatus*	Poll & Gosse, 1995	*H. pauciradiatus*	Angola (Kwanza River?)
*Marcusenius pedunculatus*	David & Poll, 1937	*Pollimyrus pedunculatus*	*P. pedunculatus*	Taverne, 1971	*P. pedunculatus*	Boma, Pool Malebo
*Marcusenius petherici	Boulenger, 1898	*Pollimyrus petherici*	*P. petherici*	Levin & Golubtsov, 2018	*Cyphomyrus petherici*	Khartum, Nile River
*Marcusenius petricolus*	Daget, 1954	*Pollimyrus petricolus*	*P. petricolus*	Taverne 1971	*P. petricolus*	Markala
*Marcusenius plagiostoma*	Boulenger, 1898	*Pollimyrus plagiostoma*	*P. plagiostoma*	Stiassny et al., 2021	*Cyphomyrus plagiostoma*	Matadi, Lower Congo River
*Marcusenius pulverulentus*	Boulenger, 1899	*Pollimyrus pulverulentus*	*P. pulverulentus*	Taverne 1971	*P. pulverulentus*	Mbandaka, Middle Congo River
*Marcusenius rudebeckii*	Svensson, 1933	*P. isidori*	*P. isidori*	Taverne 1971 (jun. syn.)	*P. isidori*	Gambia River
*Pollimyrus schreyeni*	Poll, 1972		*P. schreyeni*	Taverne, 1972	*P. schreyeni*	Boende

Species	Author(s)	Taverne, 1971a, 1971b	Taverne, 1972	Latest taxonomic revision	Current status	Occurrence
*Marcusenius squalostoma	Boulenger, 1915	*Pollimyrus squalostoma*	*Petrocephalus squalostoma*	Taverne, 1972	*P. squalostoma*	Lukinda River
*Marcusenius stappersii*	Boulenger, 1915	*Pollimyrus stappersii*	*P. stappersii*	Taverne, 1971	*P. stappersii*	Lukinda River
*M. stappersii kapangae*	David, 1935	*Pollimyrus kapangae*	*P. kapangae*	Taverne, 1971 (subspecies)	*P. stappersii*	Shaba
*Marcusenius tumifrons*	Boulenger, 1902	*P. tumifrons*	*P. tumifrons*	Taverne, 1971	*P. tumifrons*	Mobayi‐Mbongo, Ubangi River
*Petrocephalus vanderbilti*	Fowler, 1936	*P. fasciaticeps*	*P. fasciaticeps*	Taverne, 1971 (jun. syn.)	*P. isidori*	Sibut
**Total valid**		**25**	**23**		**18**	

*Note*: The species are sorted alphabetically based on their species names used in their original description. The generic name from the original description is also provided. Taxa indicated with an * are not further discussed in this study, as these concern junior synonyms or species assigned to other genera. The type species of the genus is highlighted in bold.

Abbreviation: Jun. syn., junior synonym.

Identifying collected specimens from the Luki (Democratic Republic of the Congo), Kouilou‐Niari (Republic of the Congo), Buzi (Mozambique), and the Lugenda (Mozambique) rivers was hindered by the lack of an updated alpha‐taxonomical synthesis of the genus since its original description by Taverne (1971a, 1971b, 1972), which goes beyond mere listing of species. Further, it soon became clear that there were also other taxonomical problems within this genus, at least, for the Congo basin (see also, e.g., Hopkins et al., 2007). Therefore, in this study, a taxonomic overview of the species of *Pollimyrus* is provided based on a detailed morphometric re‐examination of the type specimens of all nominal species; taxonomical issues encountered during this re‐examination are discussed and, based on this new synthesis, four potential new species for science are analysed: *Pollimyrus* sp. “*luki*,” *Pollimyrus* sp. “*kouilou‐niari*,” *Pollimyrus* sp. “*buzi*,” and *Pollimyrus* sp. “*lugenda*.” Finally, the potential implications of the geographical distribution of the genus and its constituting species on their conservation are also discussed.

## 2 MATERIALS AND METHODS

### 2.1 Taxa and specimens examined

To provide a morphological synthesis, the type specimens of all but one (i.e., *Pollimyrus macroterops*; see later) currently valid nominal species placed in the genus *Pollimyrus* were studied. Only type specimens were selected, except for a few specific cases (see later), to provide a morphometric delineation of each of the type series as an initial taxonomic basis for the genus. Non‐type specimens have been identified in the past based on resemblance to types and/or to descriptions of species or on geographical proximity to the type location. However, these could potentially represent other species than the one they are currently assigned. This is largely due to a lack of an updated taxonomic overview of the species in the genus (personal observation; Hopkins et al., 2007). Therefore, these were generally not included in this study.

Some species, currently assigned to the genera *Cyphomyrus* Myers, 1960, and *Petrocephalus* Marcusen, 1854, were also included because, according to literature, there has been some discussion about their generic placement (*P. guttatus*, Lavoué et al., 2010; *Cyphomyrus plagiostoma*, Stiassny et al., 2021; Peterson et al., 2022). Due to the confusion in the past concerning the validity of *Pollimyrus isidori fasciaticeps* as a distinct species from *P. isidori* and *Pollimyrus osborni* (Konan et al., 2013), the holotype of this species was also included in the study. Additionally, the type specimens of the subspecies and junior synonyms of *Pollimyrus stappersii* and *Pollimyrus tumifrons*, respectively, were included, as they were available at Royal Museum for Central Africa (RMCA), where the study was performed. *Pollimyrus macroterops* was not included in this study, as this species was not considered a (potential) *Pollimyrus* until recently (Sullivan et al., 2022), which was after the data collection phase for the present study.

In total, 140 specimens, including 125 type specimens of 25 nominal species, from collections of the American Museum of Natural History, New York (AMNH), Academy of Natural Sciences of Drexel University, Philadelphia (ANSP), Muséum National d'Histoire Naturelle, Paris (MNHN), Natural History Museum, London (NHM), Royal Museum for Central Africa, Tervuren (RMCA), South African Institute for Aquatic Biodiversity, Makhanda (SAIAB), and Zoologische Staatssammlung München, München (ZSM) were examined (Table 2). The four species that are new to science are described in detail. The previously described nominal species are only discussed within the scope of genus and species delineations.

**TABLE 2:** Overview of the number of studied specimens per species.

Species	Holotype	Paratype	Syntype	Non‐types	Total
*P. adspersus*			2		2
*P. brevis*			2		2
*P. castelnaui*			2		2
*P. cuandoensis*	1	11			12
*P. eburneensis*	1	7			8
*P. guttatus*	1	3			4
*P. isidori*	1 + 1			5	7
*P. maculipinnis*	1	1			2
*P. marianne*	1	10			11
*P. nigricans*			9		9
*P. nigripinnis*			7	5	12
*P. osborni*	1			5	6
*P. pedunculatus*	1				1
*P. petricolus*	1	3			4
*C. plagiostoma*			2		2
*P. pulverulentus*			4		4
*P. schreyeni*	1	3			4
*P. stappersii*	1 + 1				2
*P. tumifrons*	1 + 1 + 1				3
*P*. sp. “*luki*”	1	17			18
*P*. sp. “*lugenda*”	1	1			2
*P*. sp. “*kouilou‐niari*”	1	20			21
*P*. sp. “*buzi*”	1	1			2
Total	16 + 4	77	28	15	140

*Note*: See list of comparative material for more details. Holotypes of junior synonyms and subspecies are noted as +1 in the holotype column.

### 2.2 Morphology

**FIGURE 1:**
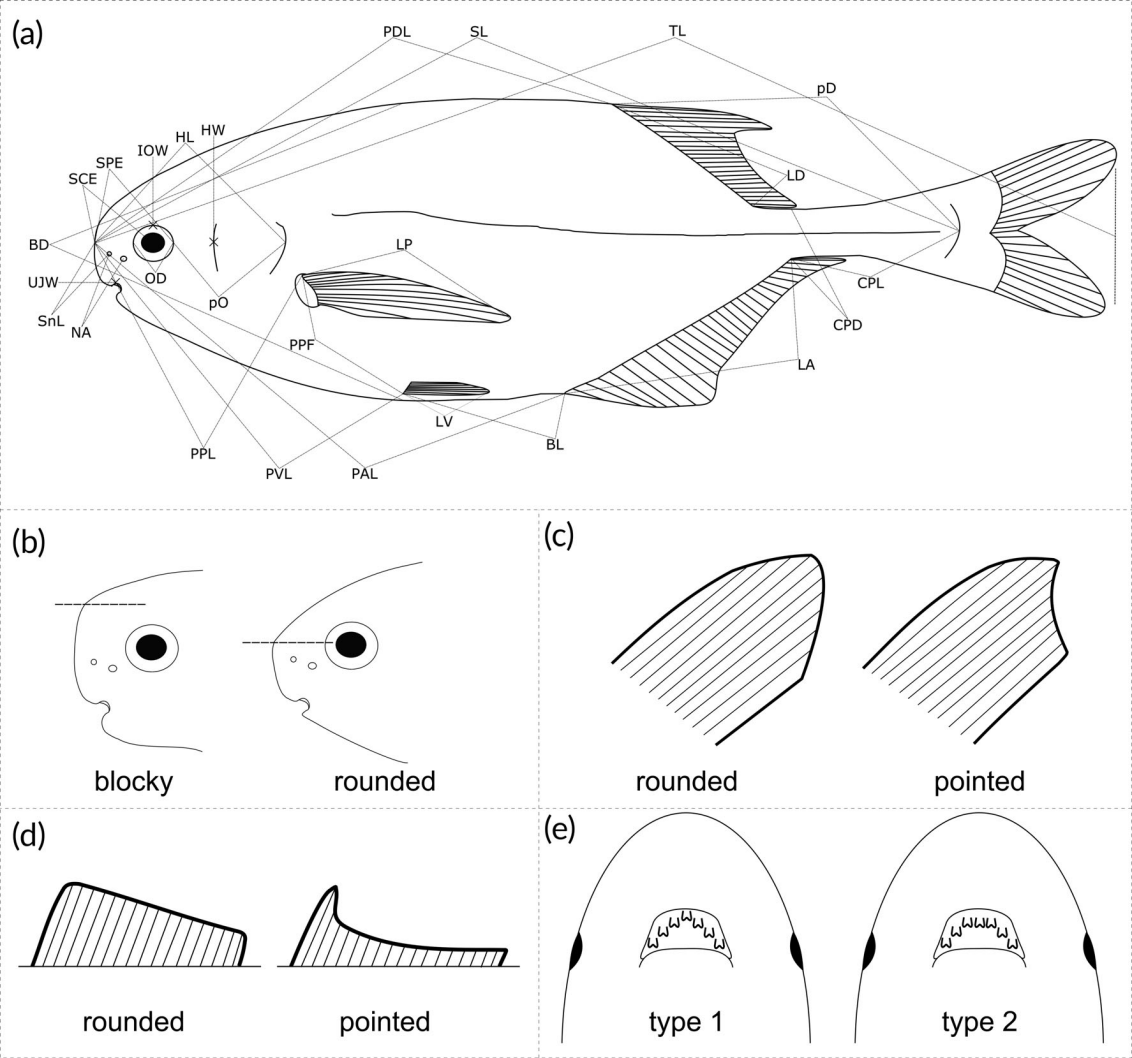
Methodology of measurements taken and qualitative observations made on *Pollimyrus* specimens. (a) Measurements (see Table 3 for abbreviations and further explanation) (based on Boden et al., 1997; Figure 1). (b) Shape of head: a blocky snout with the angle between the anterior most/vertical side of the snout and the dorsal part of the head positioned above the level of the eye (indicated by a dotted line) with mental lobe or a rounded snout with the angle at the level of the eye without mental lobe. (c) Pectoral‐fin shape: rounded with convex diminution of the shorter rays or pointed with concave diminution of shorter rays. (d) Dorsal‐ and anal‐fin shape: rounded with a rather straight overall edge or pointed with concave edge. (e) Dentition type: type 1 dentition, with one tooth occupying the most anterior and middle position, or type 2 dentition, with three teeth on one line occupying the most anterior and middle position together.

In total, 27 measurements, 12 meristics (including the number of vertebrae obtained through X‐rays, using a radiograph cabin VisiX–MedexLoncin SA 2011 [www.medex.be] with a DeReO WA detector and a GemX‐160 generator, housed at the RMCA) and 9 qualitative observations on the body, including fin shape, head shape, and dentition, were carried out. The measurements and meristics taken follow Boden et al. (1997) and Kramer et al. (2013) (Table 3). Two additional measurements and qualitative characteristics were also included (Table 3). Abbreviations and short explanations of these measurements and meristics are given in Table 3. A visual overview of the measurements and some of the qualitative features is given in Figure 1. Specimens were not sexed, as potential differences between males and females were hard to see using the shape of the anal fin (e.g., Greisman & Moller, 2005), and it is not clear if the studied species show the same sexual dimorphism as found in other mormyrid species.

**TABLE 3:** Measurements, meristics, and qualitative observations taken in this study.

Measurements (Figure 1a)	Abbrev.	Description
Standard length^a^	SL	Distance from snout to base of caudal fin
Total length^a^	TL	Distance between snout and end of tail fin
Body depth at pelvic fins^a^	BD	Distance between pelvic‐fin origin and dorsum perpendicular to pelvic‐fin origin
Pre‐dorsal length^a^	PDL	Distance between snout and dorsal‐fin origin
Pre‐anal length^a^	PAL	Distance between snout and anal‐fin origin
Pre‐pectoral length^a^	PPL	Distance between snout and pectoral‐fin origin
Pre‐pelvic length^a^	PVL	Distance between snout and pelvic‐fin origin
Length of dorsal fin^a^	LD	Distance between dorsal‐fin origin and end
Length of anal fin^a^	LA	Distance between anal‐fin origin and end
Length of pectoral fin^a^	LP	Distance between pectoral‐fin origin and tip
Length of pelvic fin^a^	LV	Distance between pelvic‐fin origin and tip
Posterodorsal distance^b^	pD	Distance between dorsal‐fin origin and posterior end of last vertebra
Pectoral–pelvic fin distance^b^	PPF	Distance between pectoral‐ and pelvic‐fin origins
Caudal peduncle length^a^	CPL	Distance between anal‐fin end and posterior end of last vertebra
Caudal peduncle depth^a^	CPD	Distance between anal‐fin end and dorsal side of caudal peduncle perpendicular to anal‐fin end
Belly length	BL	Distance between pelvic‐ and anal‐fin origins
Head length^a^	HL	Distance between snout and the most posterior border of opercle
Head width^a^	HW	Distance between the most posterior borders of left and right opercles
Postorbital length^a^	pO	Distance between posterior border of eye and most posterior side of opercle
Snout–eye posterior distance^b^	SPE	Distance between snout and posterior side of orbit
Snout–eye center distance^b^	SCE	Distance between snout and center of eye
Snout–anterior nostril length	Snl	Distance between snout and anterior nostril
Internasal length^a^	NA	Minimum distance between both nostrils
Eye diameter^a^	OD	Distance between the most anterior and the most posterior edge of eye
Interorbital width^a^	IOW	Distance between both left and right most dorsal sides of eyes
Upper‐jaw width	UJW	Distance between left and right most posterior parts of upper jaw
Lower‐jaw width	LJW	Distance between left and right most posterior parts of lower jaw
**Meristics**		
Dorsal‐fin rays^a^		Number of dorsal‐fin rays
Anal‐fin rays^a^		Number of anal‐fin rays
Pectoral‐fin rays^a^		Number of pectoral‐fin rays
Pelvic‐fin rays^a^		Number of pelvic‐fin rays
Upper‐jaw teeth^a^		Number of teeth in the lower jaw
Lower‐jaw teeth^a^		Number of teeth in the upper jaw
Lateral‐line scales^a^		Number of scales along the lateral line
Transverse dorsal–lateral lines scales^a^		Number of scales between dorsal‐fin origin and lateral‐line scales
Transverse anal–lateral‐line scales^b^		Number of scales between anal‐fin origin and lateral‐line scales
Transverse pelvic–lateral‐line scales^a^		Number of scales between pelvic‐fin origin and lateral‐line scales
Circumpeduncular scales^a^		Number of scales around the caudal peduncle at the level of anal‐fin end
Vertebrae		Number of vertebrae, counted using X‐ray photographs

Qualitative observation (Figure 1b–e)	Abbrev.	Description
Lowest nostril		Nostril closest to mouth level
Head shape		Blocky or rounded snout (Figure 1b)
Chin		Presence/absence of enlargement or bump on lower jaw (Figure 1b)
Pectoral‐fin shape		Shape of distal edge of pectoral fin (Figure 1c)
Dorsal‐fin shape		Shape of distal edge of dorsal fin (Figure 1d)
Anal‐fin shape		Shape of distal edge of anal fin (Figure 1d)
Teeth shape		Shape of teeth in both upper and lower jaws
Teeth position		Relative position of teeth in upper jaw (Figure 1e)

*Note*: See Figure 1 for the landmarks related to each measurement and illustrations showing the different morphological traits.

Abbreviation: Abbrev., abbreviation.

^a^ Meristics and measurements following Boden et al. (1997).

^b^ Additional measurements and meristics following Kramer et al. (2013).

### 2.3 Analysis

Principal component analysis (PCA) was performed in Past (version 3.16; Hammer et al., 2001) to explore the multivariate dataset. Measurements were log transformed, and the covariance matrix used. Total length was excluded. When using log‐transformed measurements, the individual loads of all variables on the first principal component (PC 1) are of the same magnitude and sign, and PC 1 can therefore be regarded as a proxy for multivariate size (Jolicoeur, 1963; Snoeks, 2004; Van Steenberge et al., 2015; also see Figure S21 in the Supplementary file S1). The correlation matrix was used for the raw meristic data.

**FIGURE 2:**
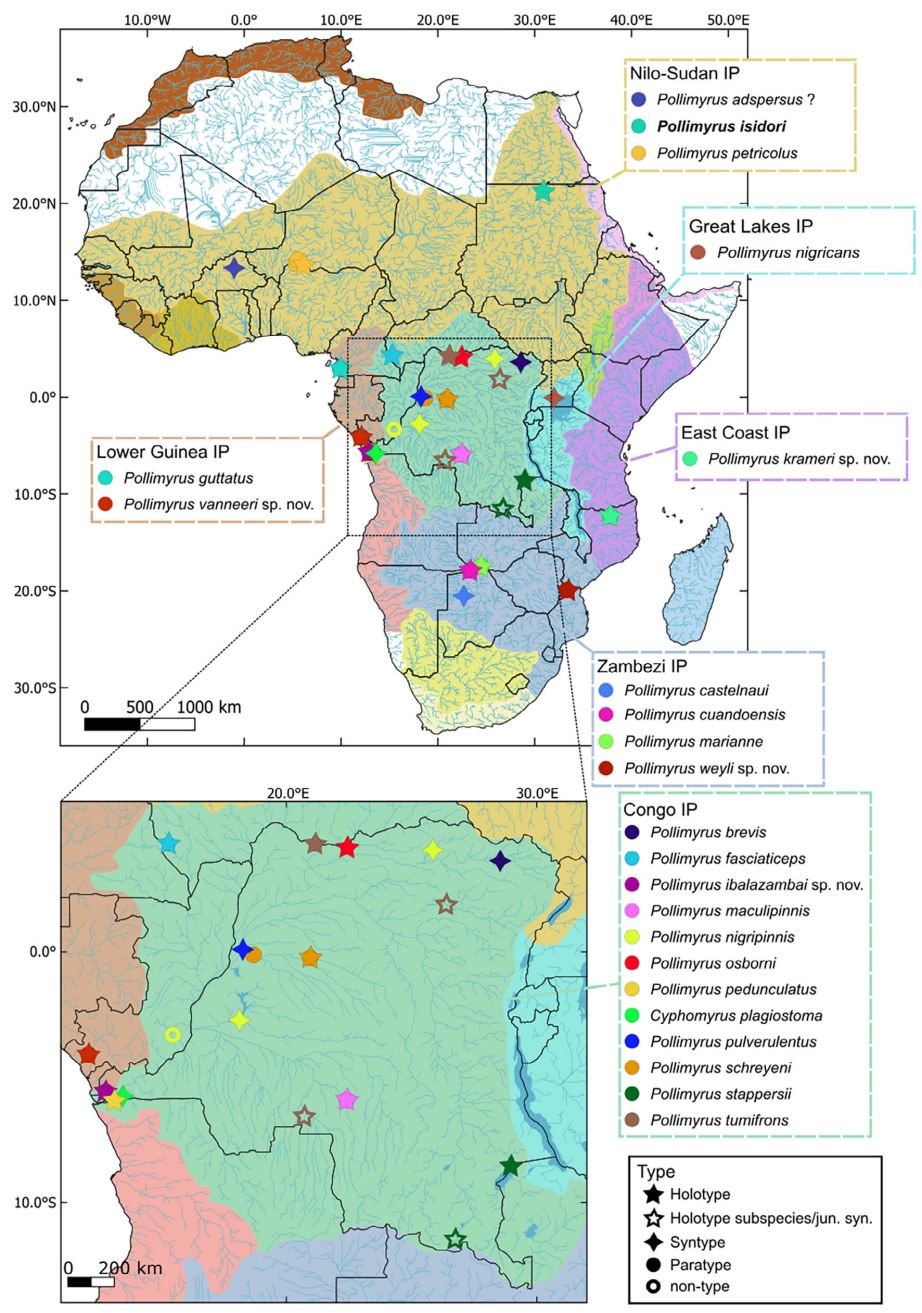
Map showing catch locations of the specimens studied of all nominal *Pollimyrus* species retained or reallocated to the genus and the four species identified as new to science, divided by ichthyogeographical province (IP; colored areas) following Lévêque and Paugy (2017). The precise type locality for *Pollimyrus isidori* and *Pollimyrus adspersus* is unclear.

Based on the measurements, meristic, and qualitative observations collected, it was first verified whether the studied specimen of each species should be assigned to the genus *Pollimyrus* using its present diagnosis (following Bigorne, 2003; Hopkins, 2007; Taverne, 1971a, 1971b; Taverne, 1972) or, instead, might need reassignment to another genus. Subsequently, specimens and species within the genus *Pollimyrus* were classified into morphological groups. Every specimen and species was then compared to all other nominal species of the same group to re‐evaluate the validity of the individuals composing nominal species and/or identify it as a potential new species for science. A map with all the localities of the studied specimens per species is provided in Figure 2.

DNA analysis was not included in this study as it intended to work only on the type series of the nominal valid species. Destructive sampling would have been too impactful for the preservation of these specimens, many of which have been collected over a century ago. Although DNA analyses on preserved mormyrid specimens have become more successful recently (e.g., Sullivan et al., 2022), this technique was not proven to be as successful during the time of the data collection of this study (2019). There was no guarantee that DNA data could be obtained for each species, making an in‐depth comparison not possible.

### 2.4 ETHICAL STATEMENT

The specimens from the Buzi River system in the Manica Province, Mozambique, were collected with a Samus electro‐fisher during a survey of the Chimanimani Transfrontier Conservation Area funded by the Transfrontier Conservation Areas and Institutional Strengthening Project based in Maputo. Specimens were killed by overdosing in clove oil and fixed in 10% formalin immediately after. The preserved specimens were sent to SAIAB (Makhanda, South Africa).

The specimens from the Rovuma River system in the Niassa Province, Mozambique, were collected with a 5 m seine net during a biodiversity survey for the Sociedade para a Gestão e Desenvolvimento da Reserva do Niassa (SRN) who manages the Niassa Reserve. Specimens were killed by overdosing in clove oil and immediately after were fixed in 10% formalin. The preserved specimens were sent to SAIAB (Makhanda, South Africa).

The specimens from the Luki Basin were collected using gill nets and traps as part of the MbiSa‐Congo project (2013–2019) by collaborators of the Institut Supérieur Pédagogique de Mbanza‐Ngungu. Specimens were also killed by overdosing in clove oil and fixed in 10% formalin immediately after. The collection missions were approved by the political‐administrative authorities of the province of Kongo Central (DRC). The preserved specimens were sent to the RMCA (Tervuren, Belgium).

## 3 RESULTS

### 3.1 Generic (re)assignments

In this section, the generic assignments for several species are investigated as these are still under debate or unresolved. These species have been the topic of recent assignments to other genera (*Cyphomyrus plagiostoma*; Stiassny et al., 2021; Peterson et al., 2022) or have been suggested to belong to *Pollimyrus* (*Petrocephalus guttatus*; Lavoué et al., 2010). Additionally, the generic status of *Pollimyrus eburneensis* is analysed due to its clear morphological differentiation from other *Pollimyrus* and specifically its type species *P. isidori*.

*Pollimyrus eburneensis* Bigorne, 1990 (Figure S1) was originally described from the Agnébi basin in Ivory Coast in a stream near the village Attienguié (Daget & de Rham, 1970) as a valid subspecies of *Pollimyrus kingsleyae* Günther, 1896 (now *Paramormyrops kingsleyae*). Despite *P. kingsleyae* having been reassigned to *Brienomyrus* Taverne, 1971 (Teugels & Hopkins, 1998) and later *Paramormyrops* Taverne, Thys van den Audenaerde and Heymer, 1977 (Hopkins et al., 2007), *P. eburneensis* was only recently elevated to species level but remained assigned to the genus *Pollimyrus* (Rich et al., 2017). However, *P. eburneensis* is clearly distinct from all other *Pollimyrus* species by having a more elongated body (body depth [BD] 18.7%–21.3% standard length [SL] vs. 22.2%–35.7% SL; 31.9% SL in *P. isidori*; 22.5%–25.7% SL in *Pollimyrus petricolus*, another elongated species found in West Africa), a deeper caudal peduncle (32.7%–33.6% BD vs. 12.8%–31.0% BD; 23.3% BD in *P. isidori*; 25.4%–28.8% BD in *P. petricolus*), and a, usually, smaller eye diameter (10.5%–14.6% head length [HL] vs. 13.7%–28.7% HL; 22.9% HL in *P. isidori*; 21.6%–23.7% HL in *P. petricolus*). Further, the ventral hypurals are visible and unfused on the X‐rays of the largest specimens (all those >53 mm SL), including the holotype of *P. eburneensis*, whereas fused is diagnostic for the genus *Pollimyrus* according to Taverne (1971a, 1971b). A sharp X‐ray photograph could not be obtained for the smallest of the *P. eburneensis* paratypes. In conclusion, this species is not considered a valid member of the genus *Pollimyrus* (see Discussion), but its characteristics currently match best with those of the genus *Paramormyrops* (see Sullivan et al., 2016).

*Petrocephalus guttatus* Fowler, 1936 (Figure S2) was originally described from near Kribi in Cameroon. This species has been reported to be morphologically more similar to the species of the genus *Pollimyrus* rather than *Petrocephalus* Marcusen, 1854 (Lavoué et al., 2010). Observations of the type specimens of this species show that its nostrils are placed further apart from each other than the posterior nostril is to the eye, as is typical in *P. isidori* and other members of the genus *Pollimyrus*, whereas in *Petrocephalus* the nostrils are positioned closer together than one is to the eye (see Figures S1–S23). The species has 7–8 teeth in the lower jaw (Table 6), whereas most *Petrocephalus* species have 14–37, including 22–29 in *Petrocephalus bane* (Lacepède, 1803), the type species of the genus (Kramer & van der Bank, 2000; Lavoué et al., 2004; Lavoué et al., 2010; Stiassny et al., 2007). Furthermore, the mouth is placed clearly anteriorly to the level of the eye (Figure S5), similar as in *Pollimyrus*, and all meristics (Table 7) fall within the ranges known for the other species of the genus *Pollimyrus*. Because there is no further resemblance to *Petrocephalus*, the species is reassigned to *Pollimyrus* (see Discussion) and is further referred to as *Pollimyrus guttatus* in this study.

*Cyphomyrus plagiostoma* (Boulenger, 1898) (Figure S3) was originally described from the Lower Congo River near Matadi (DRC). Although previously being placed in *Pollimyrus* (Taverne, 1971a, 1971b), the species has recently been reassigned to *Cyphomyrus* based on morphological evidence and tentative unpublished genetic evidence (Stiassny et al., 2021). A more recent study, however, placed the species in the genus *Pollimyrus* based on genetic analysis (Peterson et al., 2022). A morphological comparison of this species with the two genera is considered here. *C. plagiostoma* shares some characteristics with *P. isidori* and other species assigned to the genus *Pollimyrus*, whereas in others it more closely resembles *Cyphomyrus* spp., such as *Cyphomyrus petherici* (Figure S4). Its dorsal fin has more fin rays than in *Pollimyrus* species (32–33 vs. 14–28; 20 in *P. isidori*; 30–37 in *Cyphomyrus psittacus*, type species of *Cyphomyrus*), and its anal fin is shorter than the dorsal fin (73.3%–76.9% dorsal fin length (LD) vs. 90.0%–233.1% LD; 119.4% LD in *P. isidori*; 60.7%–63.6% LD in *C. petherici*), thus being more similar to the members of the genus *Cyphomyrus*. The dorsal‐fin origin, however, is positioned only slightly anterior to the anal‐fin origin like in *P. isidori* and other *Pollimyrus* species. *Cyphomyrus plagiostoma* has 9 teeth in the upper jaw and 8–10 in the lower jaw, whereas *Pollimyrus* species have 7–10 teeth in the upper jaw and 8–11 teeth in the lower jaw. The tail complex of *C. plagiostoma* also has fused ventral hypurals, which is diagnostic for the genus *Pollimyrus* following Taverne (1971a, 1971b). Further, the caudal peduncle is very thin relative to the BD compared to other *Cyphomyrus* species (13.3%–13.9% BD in *C. plagiostoma* vs. 22.7%–23.7% BD in *C. petherici*, although this falls within the ranges found in members of the genus *Pollimyrus*, 12.7%–31.2% BD; 23.3% BD in *P. isidori*). Due to this overall intermediate morphological state, its current generic position is inconclusive and awaits further data. For reasons of nomenclatorial stability, however, this species is further referred to as *Cyphomyrus plagiostoma* in this study, as this is the currently valid status (Fricke et al., 2024; see also Discussion).

*Pollimyrus guttatus* and *C. plagiostoma* are further included in this study and compared to the other species assigned to *Pollimyrus*. *Paramormyrops eburneensis* is not further analysed.

### 3.2 The delineation of morphological groups, species complexes, and species within the genus *Pollimyrus*

**FIGURE 3:**
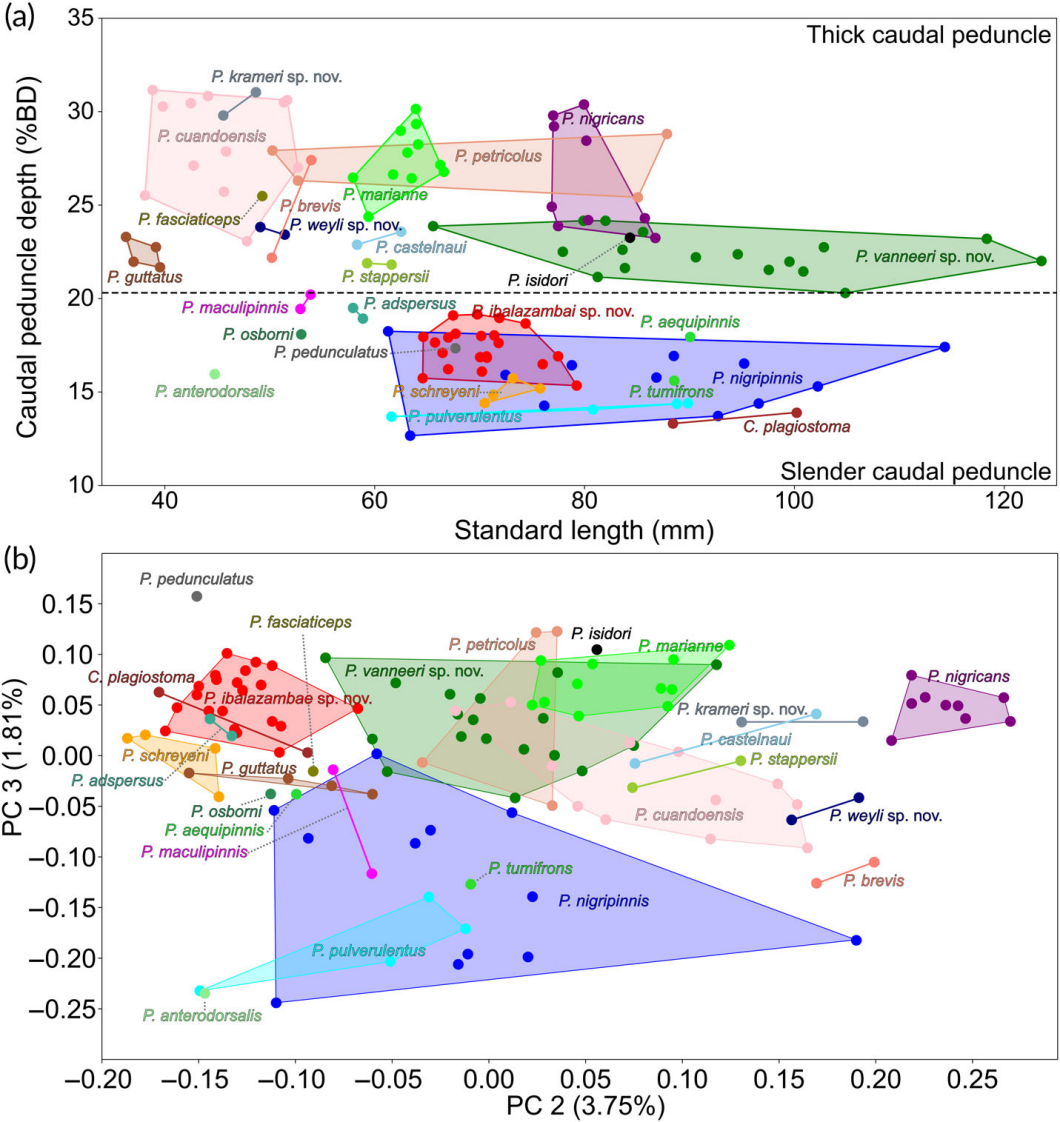
Differentiation of morphological groups. (a) Caudal peduncle depth (CPD) (% BD) against standard length (SL) (mm) for all *Pollimyrus* specimens examined (*n* = 140) (see Table 4); a differentiation can be seen between 0.20 and 0.21 CPD/BD (dotted line). (b) Scatterplot of second principal component (PC 2) against PC 3 for a principal component analysis (PCA), with explained variance between brackets, on 26 log‐transformed measurements for all *Pollimyrus* specimens examined (*n* = 140) (see Table S1 for the loadings and Figure S21 for a plot of PC 1 against SL).

Between the type specimens of *Pollimyrus*, five morphological groups can be differentiated based on the caudal peduncle depth and several qualitative characteristics, that is, snout shape (Figure 1b), the presence or absence of a mental lobe (Figure 1b), the shape of the pectoral fins (Figure 1c), and dorsal and anal fins (Figure 1d) (Table 4; Figure 3). Meristic data did not show differentiation between these groups. Each morphological group is named after the main characteristic defining it, and for the smaller groups, the main taxon in it.

**TABLE 4:** Qualitative characteristics (see text, Table 3, and Figure 1b–e for definitions) and caudal peduncle depth (CPD% BD) of the five morphological groups retained within *Pollimyrus*.

Morphological group	Species	Snout shape	Mental lobe	Pectoral‐fin shape	Dorsal‐ and anal‐fin shape	CPD (% BD)
Thick‐tailed group	*P. brevis*	Rounded	Small	Rounded	Rounded	22.2–27.4
	*P. castelnaui*	Rounded	Small	Rounded	Rounded	22.9–23.6
	*P. cuandoensis*	Rounded	Small	Rounded	Rounded	23.1–31.2
	*P. guttatus*	Rounded	Small	Rounded	Rounded	21.7–23.3
	*P. marianne*	Rounded	Small	Rounded	Rounded	24.4–30.1
	*P. petricolus*	Rounded	Small	Rounded	Rounded	25.4–28.8
	*P. stappersii (+ P. s. kapangae)*	Rounded	Small	Rounded	Rounded	21.8–21.9
	*P*. sp. “*buzi*”	Rounded	Small	Rounded	Rounded	23.4–23.8
	*P*. sp. “*lugenda*”	Rounded	Small	Rounded	Rounded	23.4
*P. isidori* group	*P. isidori*	Blocky	None	Pointed	Longer, rounded	23.3
	*P. nigricans*	Blocky	None	Pointed	Longer, rounded	23.2–30.4
	*P. fasciaticeps*	Blocky	None	Pointed	Pointed	25.5
*P*. sp. “*kouilou‐niari*”	*P*. sp. “*kouilou‐niari*”	Blocky	None	Rounded	Pointed or rounded	20.3–24.2
Slender‐tailed group	*P. adspersus*	Blocky	None	Pointed	Pointed	18.9–19.5
	*P. maculipinnis*	Blocky	None	Pointed	Pointed	19.4–20.2
	*P. nigripinnis*	Blocky	None	Pointed	Pointed	15.3–18.3
	*P. osborni*	Blocky	None	Pointed	Pointed	18.1
	*P. pedunculatus*	Blocky	None	Pointed	Pointed	17.3
	*C. plagiostoma*	Blocky	None	Pointed	Pointed	13.3–13.9
	*P. pulverulentus*	Blocky	None	Pointed	Pointed	12.7–14.4
	*P. schreyeni*	Blocky	None	Pointed	Pointed	14.9–15.7
	*P*. sp. “*luki*”	Blocky	None	Pointed	Pointed	15.3–19.2
*P. tumifrons* group	*P. tumifrons*	Rounded	None	Pointed	Pointed	15.6
	*P. anterodorsalis*	Rounded	None	Pointed or rounded	Pointed	16.0
	*P. aequipinnis*	Rounded	Small	Pointed	Pointed	17.9

Abbreviation: BD, body depth.

One can divide those *Pollimyrus* species with a thick caudal peduncle (21.7%–31.2% BD) from those with a slender caudal peduncle (12.7%–20.2% BD) (Figure 3a). The caudal peduncle depth (% BD) does not show any allometric size effect (Figure 3a). A PCA on 26 log‐transformed measurements showed that specimens with thick and slender caudal peduncle are somewhat separated by a combination of PC 2 and PC 3 (Figure 3b). The most important loadings on PC 2 are for the caudal peduncle depth, eye diameter, anal‐fin length, lower‐jaw width, and dorsal‐fin length, and on PC 3 for the caudal peduncle depth, caudal peduncle length, snout–anterior nostril length, interorbital width, and lower‐jaw width (see Table S1), verifying the separation of *Pollimyrus* on the caudal peduncle depth (% BD) into two large groups, which are further divided into morphological groups using qualitative characteristics (Table 4). PC 1 has a strong correlation with the SL (Figure S21).

Because these five morphological groups are clearly distinct from each other, these will further be analysed separately in the sections below: (1) the thick‐tailed group, (2) the *P. isidori* group, (3) the *Pollimyrus* sp. “*kouilou‐niari*” group, (4) the slender‐tailed group, and (5) the *P. tumifrons* group. Per morphological group, the differentiating criteria and the taxonomic status of each species are provided and discussed. The order in which the species are listed and discussed is determined by the ease to distinguish each of them from the other species in the group. Those species already differentiated have been left out when providing the differentiating character set for the following species.

#### 3.2.1 Thick‐tailed group

**FIGURE 4:**
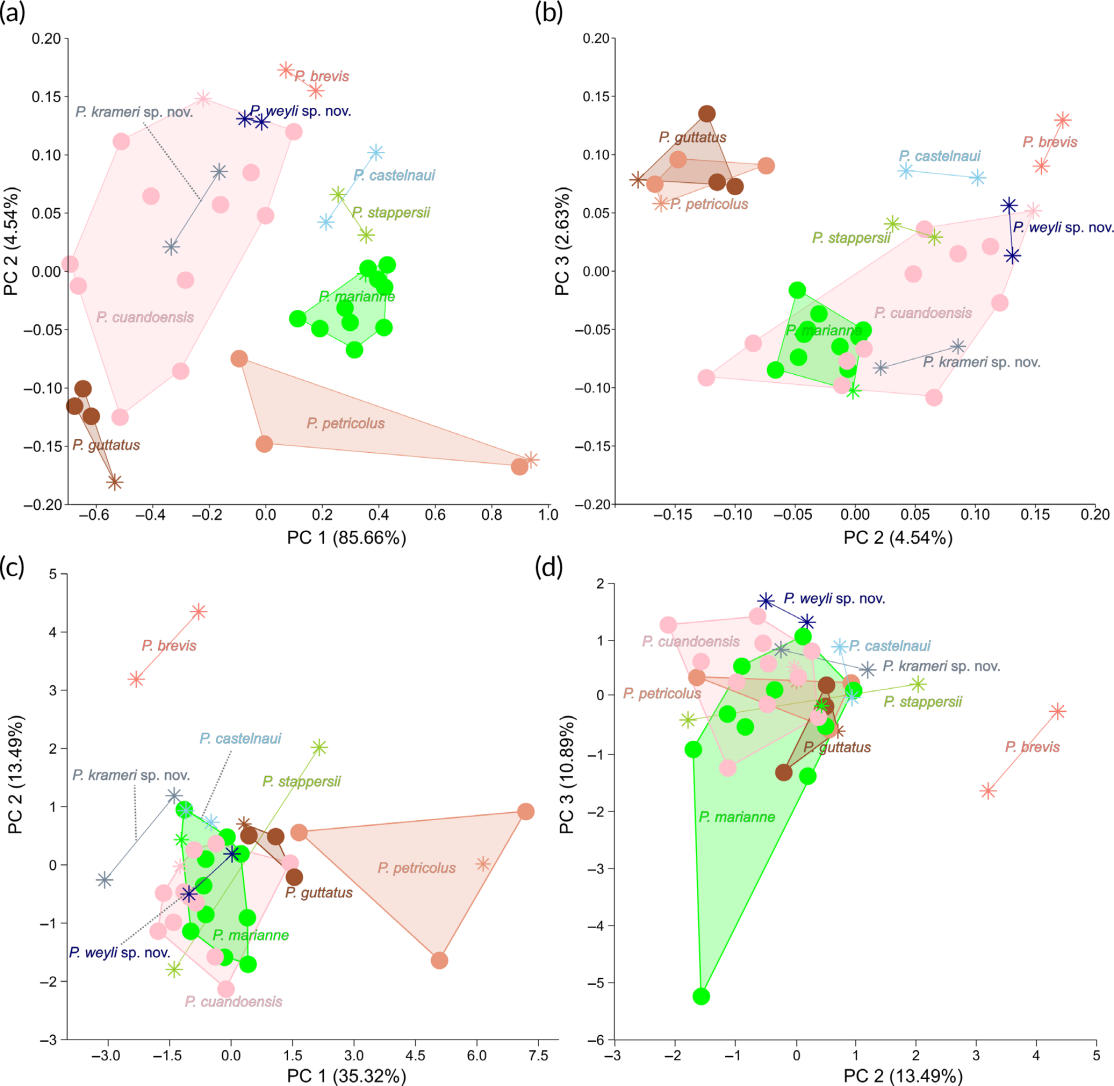
Plots of morphometric data of thick‐tailed *Pollimyrus*. (a) First principal component (PC 1) (proxy for standard length [SL]) against PC 2 for a principal component analysis (PCA) on 26 log‐transformed measurements (*n* = 41). (b) PC 2 against PC 3 for a PCA on 26 log‐transformed measurements (*n* = 41). (c) PC 1 against PC 2 for a PCA on 12 meristics (*n* = 41). (d) PC 2 against PC 3 for a PCA on 12 meristics (*n* = 41) (also see Tables 6 and 7, S2 and S3). Stars indicate holotypes and syntypes, and circles indicate paratypes. Explained variance is noted between brackets for each PC axis.

A PCA on 26 log‐transformed measurements shows a separation of *P. guttatus* and *P. petricolus* on a combination of PC 2, with the most important loadings being the lower‐jaw width, eye diameter, and the length of the dorsal fin, and PC 3, with the most important loadings being the caudal peduncle length, eye diameter, and the lower‐jaw width (Figure 4; Table S2 for the loadings). A PCA on the meristics shows a separation of *P. petricolus* on PC 1, with the most important loadings being the number of lateral‐line scales, dorsal‐fin rays, and vertebrae, and of *P. brevis* on PC 2, with the most important loadings being the number of anal‐fin rays, teeth in the upper jaw, and caudal peduncle scales (Figure 4c,d and Table S3 for the loadings).

*Pollimyrus petricolus* (Daget, 1954) (Figure S5) can be distinguished on meristics from all other thick‐tailed *Pollimyrus* species by its higher number of lateral‐line scales (58–67 vs. 40–54), usually higher number of dorsal‐fin rays (19–22 vs. 15–19), and, usually a higher number of vertebrae (41–43 vs. 36–41). The dorsal‐fin length is nearly equal (89.2%–112.2% BD) to the body depth in *P. petricolus*, whereas it is less than 82.0% (i.e., 82.0% BD for one paratype of *Pollimyrus marianne* being the closest to this range) for the other thick‐tailed *Pollimyrus*. Further, it has a shallower body depth compared to four other thick‐tailed species, *P. brevis*, *P. castelnaui, P. guttatus*, and *P*. sp. “*buzi*” (22.2–25.7% SL vs. 29.1%–32.1% SL). It differs from *P. cuandoensis* and *P*. sp. “*lugenda*” by having a longer dorsal fin (21.5%–25.0% SL vs. 16.1%–18.9% SL and 17.3%–17.5% SL, respectively). Finally, *P. petricolus* differs from *P. stappersii* by having more circumpeduncular scales (16–20 vs. 13) and from *P. marianne* by having more dorsal–lateral scales (13–14 vs. 9–11).

*Pollimyrus guttatus* (Fowler, 1936) (Figure S2) differs from *P. petricolus* by the aforementioned characteristics (see above). *Pollimyrus guttatus* differs from all other thick‐tailed species, except *P. cuandoensis* and *P. marianne*, by its larger eye diameter (22.1%–27.3% HL vs. 14.3%–19.4% HL). It differs from *P. brevis* by having a wider head (43.4%–52.4% HL vs. 58.5%–60.2% HL), a slenderer upper jaw (13.9%–15.6% HL vs. 19.1%–22.2% HL) and lower jaw (14.2%–17.3% HL vs. 23.5%–25.7% HL), a shorter postorbital distance (55.8%–58.7% HL vs. 61.3–63.5% HL), and more circumpeduncular scales (15–17 vs. 11–13). It differs from *P. castelnaui* by having a longer pectoral fin (23.2%–26.1% SL vs. 20.7%–21.0% SL), slenderer upper jaw (13.9%–15.6% HL vs. 18.0%–19.4% HL) and lower jaw (14.2%–17.3% HL vs. 21.7%–22.1% HL), and more circumpeduncular scales (15–17 vs. 12–13). It can be distinguished from *P. cuandoensis* by having a longer pectoral fin (23.2%–26.1% SL vs. 19.4–21.9% SL) and more dorsal‐fin rays (18–19 vs. 15–16). It differs from *P. stappersii* by having longer pectoral fins (23.2%–26.1% SL vs. 19.9%–20.6% SL), a shorter postorbital distance (55.8%–58.7% HL vs. 61.5%–62.3% HL), and more circumpeduncular scales (15–17 vs. 13). It differs from *P*. sp. “*buzi*” by having a slenderer upper jaw (13.9%–15.6% HL vs. 22.6%–24.5% HL), a longer anal fin (25.0%–27.1% SL vs. 20.2%–22.5% SL), and a longer pectoral fin (23.2%–26.1% SL vs. 18.1%–19.6% SL). It differs from *P*. sp. “*lugenda*” by having a deeper body (30.1%–32.1% SL vs. 25.3%–26.7% SL), a longer pectoral fin (23.2%–26.1% SL vs. 19.2%–20.5% SL), and more dorsal‐fin rays (18–19 vs. 15). Finally, it differs from *P. marianne* by having a deeper body (30.1%–32.1% SL vs. 23.7%–27.0% SL), a longer pre‐anal distance (62.2%–65.8% SL vs. 56.6%–59.7% SL), longer pre‐ventral distance (43.7%–45.7% SL vs. 38.9%–41.2% SL), and a slenderer head (43.4%–52.4% HL vs. 58.4%–61.4% HL).

*Pollimyrus brevis* (Boulenger, 1913) (Figure S6) differs from *P. marianne* by its deeper body (29.1%–32.1% SL vs. 23.7%–27.0% SL), a longer pre‐dorsal distance (67.4%–67.5% SL vs. 61.2%–65.5% SL), a longer pre‐anal distance (62.3%–64.5% SL vs. 56.6%–59.7% SL), a shorter caudal peduncle length (15.2% SL vs. 17.2%–20.5% SL), a wider lower jaw (23.5%–25.7% HL vs. 14.6%–18.8% HL), fewer lateral‐line scales (40–46 vs. 48–54), and by its most anterior teeth being the same size as the posterior teeth, and not bigger as in *P. marianne*. It can be distinguished from *P. castelnaui* by its longer pelvic fin (11.1%–11.3% SL vs. 9.7% SL), a wider head (58.5%–60.2% HL vs. 53.8%–54.1% HL), and fewer scales between the pelvic fin and the lateral line (10 vs. 13). *P. brevis* differs from *P. cuandoensis* by having a shorter caudal peduncle length (15.2% SL vs. 16.4–16.9% SL) and more dorsal‐fin rays (18 vs. 15–16). It differs from *P. stappersii* (including *P. stappersii kapangae*) by having a wider head (58.5%–60.2% HL vs. 52.7%–54.0% HL), a wider lower jaw (23.5%–25.7% HL vs. 17.9%–18.3% HL), and fewer lateral‐line scales (40–46 vs. 48–53). It can be distinguished from *P*. sp. “*buzi*” by having a longer anal fin (24.3%–24.4% SL vs. 20.2%–22.5% SL), a wider lower jaw (23.5%–25.7% HL vs. 17.1%–19.1% HL), and fewer lower‐jaw teeth (seven vs. nine). It differs from *P*. sp. “*lugenda*” by having a deeper body (29.1%–32.1% SL vs. 25.3%–26.7% SL), a longer dorsal fin (19.1%–20.4% SL vs. 17.3%–17.5% SL), a wider head (58.5%–60.2% HL vs. 52.7%–53.8% HL), a larger eye (18.8%–19.0% HL vs. 14.3%–15.5% HL), a wider lower jaw (23.5%–25.7% HL vs. 14.7%–16.2% HL), a wider interorbital width (39.2%–44.6% HL vs. 31.7%–33.1% HL), and more dorsal‐fin rays (18 vs. 15).

*Pollimyrus castelnaui* (Boulenger, 1911) (Figure S7) differs from *P. marianne* by having a deeper body (29.5%–31.2% SL vs. 23.7%–27.0% SL), a slenderer head (53.8%–54.1% SL vs. 58.4%–61.4% SL), a wider lower jaw (21.7%–22.1% HL vs. 14.6%–18.8% HL), a pelvic fin that is less than half the length of the belly (vs. slightly longer than half the belly length), and by its most anterior teeth being the same size as the posterior teeth (vs. anterior teeth being a bit larger). It can also be distinguished from *P. cuandoensis* by having a pelvic fin that is less than half the length of the belly. It can be distinguished from *P. stappersii/kapangae* by having a wider lower jaw (21.7%–22.1% HL vs. 17.9%–18.3% HL), having fewer scales between the lateral‐line and the anal‐fin origin (10–11 vs. 12–13), and by their geographical isolation. *Pollimyrus castelnaui* differs from *P*. sp. “*buzi*” by having a slenderer upper jaw (18.0%–19.4% HL vs. 22.6%–24.5% HL), a pelvic fin that is less than half the length of the belly (vs. slightly longer than half the belly length), and in its geographical occurrence. It differs from *P*. sp. “*lugenda*” by having a deeper body (29.5%–31.2% SL vs. 25.3%–26.7% SL), a longer snout–eye distance (42.3%–46.1% HL vs. 39.4%–39.7% HL), a larger eye (18.6%–19.9% HL vs. 14.3%–15.5% HL), a wider lower jaw (21.7%–22.1% HL vs. 14.7%–16.2% HL), a wider interorbital width (38.1%–41.5% HL vs. 31.7%–33.1% HL), more scales between the pelvic fin and the lateral line (13 vs. 11), and by having a pelvic fin that is less than half the length of the belly (vs. slightly longer than half the belly length).

Specimens found in the Buzi basin in Mozambique, here preliminarily identified as *Pollimyrus* sp. “*buzi*,” differ from *P. marianne*, *P. cuandoensis, P. stappersii/kapangae*, and *P*. sp. “*lugenda*” by having a wider upper jaw (22.6%–24.5% HL vs. 13.3%–20.2% HL). They can further be differentiated from *P. marianne* by having a deeper body (29.6%–29.9% SL vs. 23.7%–27.0% SL), a shorter post‐dorsal distance (37.6% SL vs. 39.8%–43.7% SL), and its most anterior teeth being the same size as the posterior teeth. They are distinguished from *P. stappersii/kapangae* by having a shorter belly length (17.5%–18.2% SL vs. 20.8%–21.6% SL). They further differ from *P*. sp. “*lugenda*” by having a deeper body (29.6%–29.9% SL vs. 25.3%–26.7% SL), a wider head (55.2%–58.7% HL vs. 52.7%–53.8% HL), a larger eye (17.1%–18.9% HL vs. 14.3%–15.5% HL), a wider lower jaw (22.6%–24.5% HL vs. 19.5%–19.7% HL), and a wider interorbital width (37.2%–40.9% SL vs. 31.7%–33.1% SL). As these specimens are morphologically distinct from all other *Pollimyrus*, they are proposed as a new species to science: *Pollimyrus weyli* sp. nov. (Figure 12). The detailed description of this species new to science is provided below.

Specimens found in the Ruvuma basin in Mozambique, here preliminarily identified as *Pollimyrus* sp. “*lugenda*,” differ from *P. marianne* by its shorter post‐dorsal distance (36.8%–37.0% SL vs. 39.8%–43.7% SL), a slenderer head (52.7%–53.8% HL vs. 58.4%–61.4% HL), a smaller eye (14.3%–15.5% HL vs. 17.7%–21.9% HL), and its most anterior teeth being the same size as the posterior teeth. They differ from *P. stappersii/kapangae* by having a short snout–eye length (39.4%–39.7% HL vs. 44.3%–44.5% HL). There are slight differences between these specimens and *P. cuandoensis* in eye diameter (14.3%–15.5% HL vs. 15.5%–21.9% HL) and snout–eye length (39.4%–39.7% HL vs. 40.5%–45.3% HL). As these specimens are morphologically distinct from all other *Pollimyrus*, they are proposed as a new species to science: *Pollimyrus krameri* sp. nov. (Figure 8). The detailed description of this species new to science is provided below.

*Pollimyrus marianne* Kramer et al., 2003 (Figure S8) differs from *P. stappersii/kapangae* by having a wider head (58.4%–61.4% HL vs. 52.7%–54.0% HL) and a shorter pre‐anal distance (56.6%–59.7% SL vs. 61.6%–63.5% SL). It cannot be distinguished from *P. cuandoensis* (see below).

**FIGURE 5:**
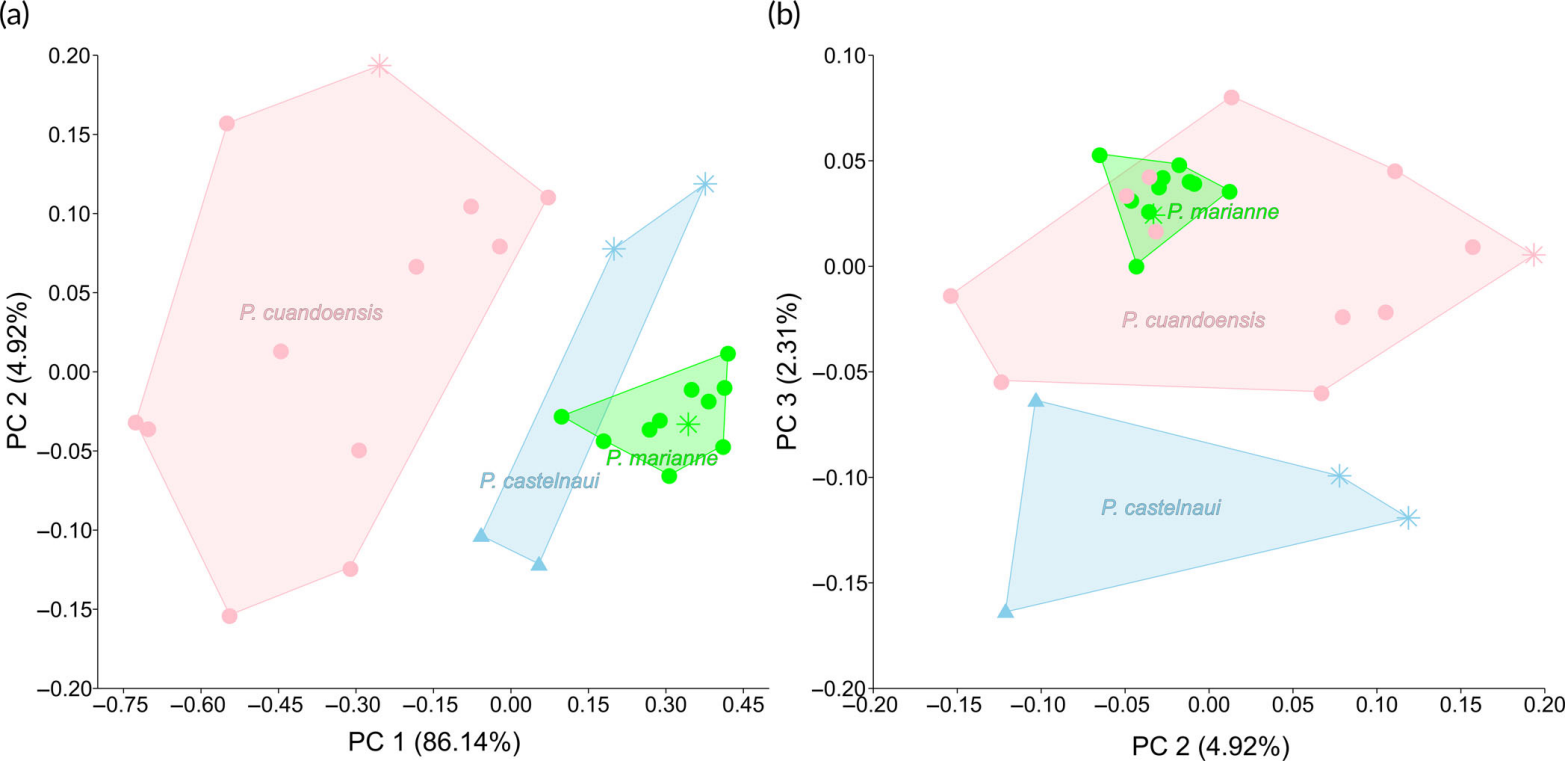
Plots of morphometric data of the marianne species complex, including *Pollimyrus marianne* Kramer et al., 2003, (*n* = 11) and *Pollimyrus cuandoensis* Kramer et al., 2013, (*n* = 12), and comparing with morphological similar yet distinct *Pollimyrus castelnaui* (Boulenger, 1911) (*n* = 4) from the Zambezi IP (see also Table S6). (a) First principal component (PC 1) (proxy for standard length [SL]) against PC 2 for a principal component analysis (PCA) on 26 log‐transformed measurements. (b) PC 2 against PC 3 for a PCA on 26 log‐transformed measurements. Stars indicate holotypes or syntypes, circles indicate paratypes, and triangles indicate non‐type specimens. Explained variance is noted between brackets for each PC axis.

*Pollimyrus cuandoensis* Kramer et al., 2013 (Figure S8) differs slightly from *P. stappersii/kapangae* by having a thicker caudal peduncle (6.6%–8.8% SL vs. 5.9%–6.1% SL), a shorter belly (17.3%–20.1% SL vs. 20.8%–21.6% SL), and more circumpeduncular scales (14–16 vs. 13). It cannot be distinguished from *P. marianne*. Because *P. marianne* and *P. cuandoensis* both occur in the Upper Zambezi basin in adjacent rivers (main Zambezi River and Kwando River, respectively), and the species don't separate on a PCA of 26 log‐transformed measurements (Figure 5; Table S6), it is suggested here to place the two species in synonymy (also see Discussion). *Pollimyrus cuandoensis* has large ranges for many measurements and meristics (Tables S11 and S12). Despite these large ranges there is no indication that these specimens belong to a different species. All type specimens of *P. cuandoensis* seem to be conspecific.

*Pollimyrus stappersii* (Boulenger, 1915) (Figure S9) differs from all other thick‐tailed species by the aforementioned characteristics. It consists of two subspecies, *P. s. stappersii* and *P. s. kapangae*. The holotype of *P. s. kapangae* shows some differences compared to that of *P. s. stappersii* and other non‐type specimens currently identified to belong to this complex (see Supplementary file S2), such as slenderer teeth, more anal‐fin rays (26 vs. 22), and fewer lateral‐line scales (48 vs. 53; although there remains some uncertainty due to the scales being covered by skin), which in our view are important differences between both (sub)species when considering the difference found between other species within the genus. However, these are insufficient to elevate *P. s. kapangae* to species level due to the lack of differences in measurements and the present lack of additional topotypic specimens. As there are no non‐type specimens available in museum collections from the same localities as the holotypes of both subspecies, this complex cannot be further analysed. Therefore, for the time being, a single species *P. stappersii* is recognized here with two valid subspecies, considering the differences found.

#### 3.2.2 *P. isidori* group

This group consists of three currently valid species: *P. isidori* (type species of *Pollimyrus*), *P. fasciaticeps*, and *P. nigricans*.

*Pollimyrus isidori* (Valenciennes, 1847) (Figure S10) differs from both the other species of the group by its higher number of dorsal‐fin rays (20 vs. 14–17 in *P. nigricans* and 17 in *P. fasciaticeps*) and more pelvic–anal scales (16 vs. 12 and 14, respectively). It can further be distinguished from both the other species by its deeper body (33.3% SL vs. 23.5%–29.5% SL in *P. nigricans* and 29.7% SL in *P. fasciaticeps*). Finally, it has very short pectoral fins that do not reach halfway of the pelvic fin (vs. almost reaching the distal tip of the pelvic fin in *P. nigricans* and *P. fasciaticeps*).

*Pollimyrus nigricans* (Boulenger, 1906) (Figure S11) differs from the other two species, *P. isidori* and *P. fasciaticeps*, by having a shorter dorsal fin (16.0%–19.1% SL vs. 22.5% SL and 22.3% SL), a shorter post‐dorsal distance (35.5%–37.5% SL vs. 42.8% SL and 43.5% SL), a larger postorbital distance (62.4%–67.3% SL vs. 57.3% SL and 52.2% HL), a slightly shorter anal fin (21.0%–24.6% SL vs. 26.8% SL and 27.7% SL), and fewer pelvic–anal scales (12 vs. 16 and 14). This species also has longer but not pointed anterior parts of the dorsal and anal fins (vs. pointed in *P. fasciaticeps*). The pectoral fins almost reach the posterior tip of the pelvic fins (vs. not reaching halfway the pelvic fin in *P. isidori*).

*Pollimyrus fasciaticeps* (Boulenger, 1920) (Figure S12) was originally described based on a single specimen. The holotype can be distinguished from the type specimens of the other two species by the longer distance between the snout and the anterior eye margin (50% HL vs. 41.4% HL in *P. isidori* and 37.5%–42.6% HL in *P. nigricans*), slightly shorter pre‐dorsal distance (61.6% SL vs. 63.8% SL in *P. isidori* and 64.5%–67.8% SL in *P. nigricans*), and having a higher number of dorsal–lateral scales (16 vs. 14 in *P. isidori* and 11–14 in *P. nigricans*). It can further be distinguished from *P. isidori* by the wider head (59.7% HL vs. 50.9% HL). This species also has pointed anterior parts of the dorsal and anal fins (vs. longer but not pointed in *P. isidori* and *P. nigricans*). The pectoral fins almost reach the posterior part of the pelvic fins (vs. not reaching halfway the pelvic fin in *P. isidori*).

#### 3.2.3 *Pollimyrus* sp. “*kouilou‐niari*” group

The specimens originating from the Kouilou‐Niari River, a coastal river basin of the Lower Guinea ichthyogeographical province (IP) in the Republic of the Congo, is here preliminarily identified as *Pollimyrus* sp. “*kouilou‐niari*.” It can be distinguished from all other *Pollimyrus* species by having a thick caudal peduncle compared to the slender caudal peduncle of all *Pollimyrus* species (20.3%–24.2% BD vs. 12.7%–20.2% BD), a blocky snout (vs. usually round), and no mental lobe (vs. small mental lobe) compared to thick‐tailed *Pollimyrus*, and rounded pectoral fin (vs. pointed) compared to the members of the *P. isidori* group.

Only one other congeneric species occurs in the same IP, *P. guttatus*, which occurs near Kribi, in the Kineke River in southern Cameroon. The specimens from the Kouilou‐Niari River can be distinguished from *P. guttatus* by having a subterminal mouth (vs. terminal mouth), a shallower body depth (22.6%–28.7% SL vs. 30.7%–32.1% SL), a shorter pre‐ventral distance (33.7%–40.2% SL vs. 43.7%–45.7% SL), more dorsal‐fin rays (21–24 vs. 18–19), more vertebrae (41–43 vs. 37–38), and more lateral‐line scales (59–73 vs. 43–52).

As these specimens are morphologically distinct from all other *Pollimyrus*, they are here identified as a new species to science: *Pollimyrus vanneeri* sp. nov. (Figure 11). The detailed description of this species new to science is provided later.

#### 3.2.4 Slender‐tailed group

**FIGURE 6:**
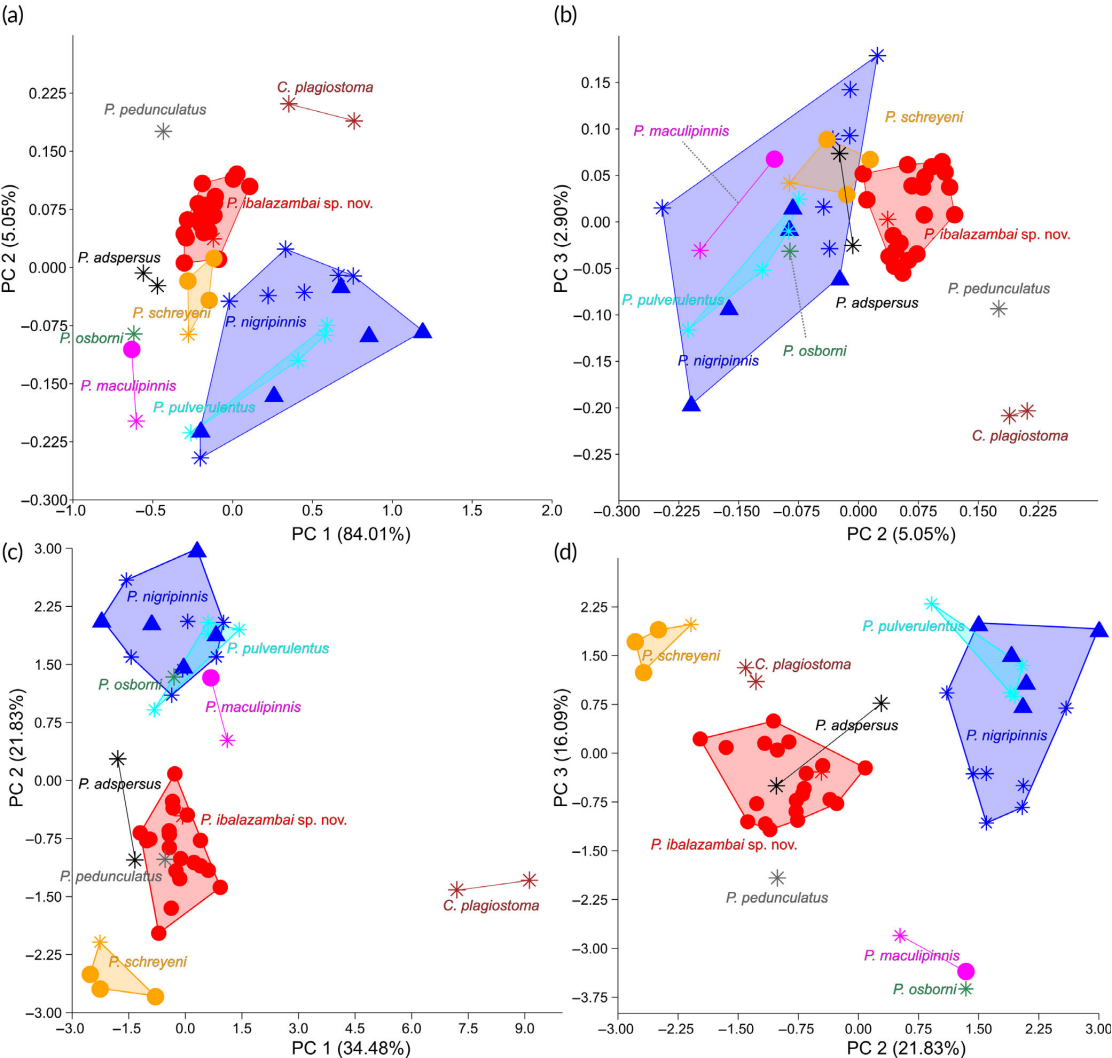
Plots of morphometric data of slender‐tailed *Pollimyrus*. (a) PC 1 (proxy for SL) against PC 2 for a principal component analysis (PCA) on 26 log‐transformed measurements (*n* = 49). (b) PC 2 against PC 3 for a PCA on 26 log‐transformed measurements (*n* = 49). (c) PC 1 against PC 2 for a PCA on 12 meristics (*n* = 49). (d) PC 2 against PC 3 for a PCA on 12 meristics (*n* = 49) (also see Tables 6, 7 and S4, S5). Stars indicate holotypes and syntypes, circles indicate paratypes, and triangles indicate non‐type specimens. Explained variance is noted between brackets for each PC axis.

A PCA on 26 log‐transformed measurements shows a slight separation of *C. plagiostoma*, *P. pedunculatus*, and *P*. sp. “*luki*” on a combination of PC 2, with the most important loadings being the caudal peduncle length, length of the dorsal fin, the posterodorsal distance, and PC 3, with the most important loadings being the snout length, the length of the dorsal fin, and the eye diameter (Figure 6b; Table S4 for the loadings; also see Figure S22). A PCA on the meristics shows a separation of *C. plagiostoma* on PC 1, with the most important loadings being the number of dorsal‐fin rays, pectoral‐fin rays, and scales between the anal‐fin origin and lateral line, *P. pulverulentus, P. nigripinnis, P. maculipinnis, P. osborni*, and *P. schreyeni* on PC 2, with the most important loadings being the number of teeth in the upper jaw, teeth in the lower jaw, and vertebrae, and *P. maculipinnis, P. osborni*, and *P. schreyeni* on PC 3, with the most important loadings being the number of anal‐fin rays, caudal peduncle scales, and the scales between the lateral line and dorsal‐fin origin (Figure 6d; Table S5 for the loadings).

*Cyphomyrus plagiostoma* (Boulenger, 1898) (Figure S4) differs from all the other slender‐tailed *Pollimyrus* by having more dorsal‐fin rays (32–33 vs. 14–28) and a dorsal fin that is longer than the anal fin (130.0%–136.4% LD vs. 42.9%–111.1% LD). These characteristics also differentiate this species from all other *Pollimyrus* species.

*Pollimyrus schreyeni* Poll, 1972 (Figure S13) has fewer scales between the pelvic fin origin and the lateral line compared to the other slender‐tailed *Pollimyrus* (9–11 [median 10] vs. 11–18 [median 14]). It differs by the number of dorsal‐fin rays from *P. pedunculatus* (17 vs. 19), from *P. adspersus* (17 vs. 19–20), from *P. pulverulentus* (17 vs. 22–24), and *P. nigripinnis* (17 vs. 18–23). It has a dorsal fin that originates clearly posteriorly to the level of the anal‐fin origin, whereas in all other *Pollimyrus* species, it originates slightly anteriorly, slightly posteriorly, or at the same level as the anal fin. Its dorsal fin is slightly shorter than half the anal fin, whereas in all other species it is longer than half this length or even slightly longer than the entire length of the anal fin (42.9%–46.8% LA vs. 63.1%–136.98% LA). The anal fin is slightly longer than one third of the standard length in *P. schreyeni* (34.7%–35.4% SL vs. 19.9%–32.1% SL in other *Pollimyrus*). It has more fin rays (30–32 vs. 20–28) than all other *Pollimyrus* species. Further, compared to all other *Pollimyrus* species, the four type specimens included in the study have a distinct colouration after preservation. These fish are generally light brown with large‐sized, dark brown spots, covering several scales, whereas most other *Pollimyrus* species are plainly colored, with sometimes a vague midlateral line or small‐sized spots, which are smaller than a scale (see Figure S16).

*Pollimyrus pedunculatus* (David & Poll, 1937) (Figure S14), only known from the holotype, is the only slender‐tailed *Pollimyrus* species in which the anterior nostril is positioned lower than the posterior one. Further, it has a long and slim caudal peduncle compared to the other slender‐tailed *Pollimyrus* species (CPD%CPL; 20.6% CPL vs. 24.4%–37.6% CPL), except for *C. plagiostoma*, which also has a rather slender tail (19.3%–25.6% CPL). The pre‐pelvic distance is shorter compared to that of all other *Pollimyrus* (34.3% SL vs. 36.0%–42.9% SL). Its pelvic fin seems shorter than in all other *Pollimyrus* species (7.0% SL vs. 8.4%–13.4% SL). However, it cannot be ruled out that this is caused by some damage, as the distal tip of the holotype's fin seems slightly tapered.

*Pollimyrus maculipinnis* (Nichols and LaMonte, 1934) (Figure S15) has a holo‐ and a paratype that are small (52.9–53.9 mm SL). This species clearly differs from the other slender‐tailed *Pollimyrus* species by several measurements and meristics. It differs from *P. nigripinnis* and *P. pulverulentus* by having a thicker caudal peduncle (5.6%–6.4% SL vs. 3.9%–5.3% SL and 4.1%–4.7% SL) and fewer teeth in the upper jaw (7 vs. 8–10 and 9–10). It differs from *P. adspersus, P*. sp. “*luki*,” *P. osborni*, and *P. pedunculatus* by having a longer head (25.3%–25.3 SL vs. 21.5%–23.7% SL). *P. maculipinnis* has fewer dorsal‐fin rays compared to the specimens identified as *P. nigripinnis*, *P. adspersus*, and *P. pedunculatus* (17–17 vs. 19–23), and fewer anal fin rays than *P. pulverulentus* and *P. schreyeni* (24–24 vs. 27–32).

**FIGURE 7:**
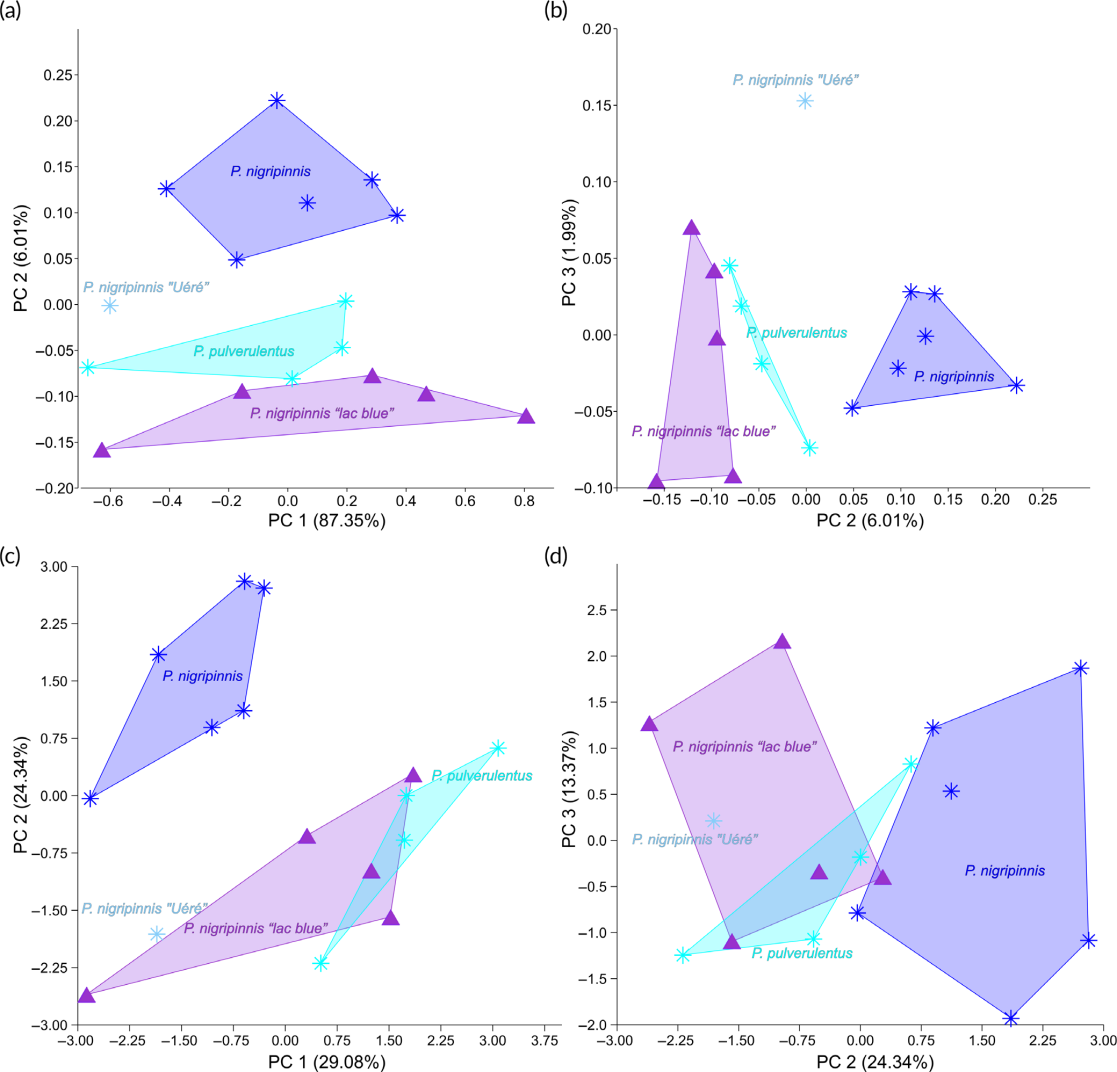
Plots of morphometric data of the *Pollimyrus pulverulentus* complex (*n* = 16). (a) PC 1 (proxy for SL) against PC 2 for a principal component analysis (PCA) on 26 log‐transformed measurements (*n* = 16). (b) PC 2 against PC 3 for a PCA on 26 log‐transformed measurements (*n* = 16). (c) PC 1 against PC 2 for a PCA on 12 meristics (*n* = 16). (d) PC 2 against PC 3 for a PCA on 12 meristics (*n* = 16) (also see Tables 6, 7, S2 and S3). Stars indicate syntypes, and triangles indicate non‐type specimens. Explained variance is noted between brackets for each PC axis.

*Pollimyrus nigripinnis* (Boulenger, 1899) (Figure S16) and *P. pulverulentus* (Boulenger, 1899) (Figure S16) are hard to distinguish from each other. Based on the collected morphometric data, the type specimens of *P. nigripinnis* and *P. pulverulentus* overlap in measurements and meristics (also see Table 5). A PCA on 26 log‐transformed measurements shows a clear separation of the two nominal species on PC 2, with the most important loadings being the snout length, belly length, and the caudal peduncle length. The same is true for a PCA on the meristics on PC 2, with the most important loadings being the number of vertebrae, caudal peduncle scales, and lateral‐line scales (Figure 7; also see Tables S7 and S8 for the loadings). However, they cannot be distinguished visually on any qualitative or quantitative characteristic, and the ranges for the measurements and meristics do not allow for a clear separation due to overlap. With the type localities of both nominal species being in the Middle Congo basin (Figure 2), they might belong to one and the same species. Therefore, both species are regarded as synonyms and from now on referred to as *P. pulverulentus* (Boulenger, 1899) (see Discussion for more details). Non‐type specimens identified to the *P. pulverulentus* complex found in Lac Bleu appear to resemble the types of *P. pulverulentus* most morphologically, as seen in the observed ranges for the different nominal species (Table 5) and in PCAs on the measurements and meristics (Figure 7).

**TABLE 5:** Overview of the ranges for the most differentiating morphometric characteristics of the species in the *Pollimyrus pulverulentus* complex.

Measurement	*Pollimyrus pulverulentus* syntype (*n* = 4)	*Pollimyrus nigripinnis* syntype (*n* = 6)	*P. nigripinnis* “Uéré” syntype (*n* = 1)	*P. nigripinnis* “lac bleu” (*n* = 5)
Characteristic (in % SL)				
BD	30.0–33.4	28.4–30.7	28.2	30.7–35.7
BL	18.1–23.6	22.2–24.7	21.6	19.0–22.4
PPL	29.2–30.1	26.6–28.7	29.8	27.9–30.2
LD^a^	22.8–25.4	20.9–23.6	18.5	22.3–24.8
LA	28.6–30.8	27.1–28.7	28.1	28.0–29.8
LV	11.3–12.3	9.9–11.1	11.8	10.8–12.4
CPL	14.0–16.3	16.4–18.1	16.5	13.6–17.3
HL^a^	24.9–26.8	23.5–25.3	27.1	25.0–27.7
Characteristic (in % BD)				
CPD^a^	13.7–14.4	15.3–16.9	18.3	12.7–17.4
Characteristic (in % HL)				
SnL	16.4–16.7	9.8–17.4	15.4	18.2–20.0
HW	47.7–54.7	47.5–51.3	49.3	47.6–54.1
Na	7.7–8.3	6.8–8.2	7.8	8.5–9.1
OD^a^	22.2–24.5	22.8–24.2	25.3	17.5–24.9
IOW^a^	29.8–38.0	31.1–35.7	38.7	31.1–39.1
UJW	16.2–19.5	15.0–17.8	17.2	15.5–21.5
LJW^a^	15.6–17.9	15.9–18.6	18.8	14.2–20.8
Meristic				
Dorsal‐fin rays^a^	22–24	19–21	18	19–23
Anal‐fin rays	27–28	25	25	25–27
Vertebrae^a^	38	38–40	37	37–39
Upper‐jaw teeth^a^	9–10	8–9	10	9–10
Lateral‐line scales	49–54	47–54	47	42–52
Caudal peduncle scales	11–14	12–16	13	11–12

*Note*: See Table 3 and Figure 1 for definitions of the abbreviations. The full list of morphometric characteristics can be found in Table S9.

^a^ Indicates the measurements and meristics where the syntype series of *P. nigripinnis* (Boulenger, 1899) differ.

*Pollimyrus pulverulentus* differs from *P. adspersus*, *P. osborni, P. pedunculatus*, and *P*. sp. “*luki*” by having a longer pre‐pectoral distance (26.6%–30.2% SL vs. 20.1%–25.9% SL). It also differs in dentition, having 8–10 teeth in the upper jaw and 9–11 teeth in the lower jaw, from *P. osborni* (7 teeth in the upper jaw) and *P. adspersus* (8 teeth in the lower jaw). Their teeth have small cusps that are not as obvious as those of *P. adspersus*, *P. fasciaticeps*, *P. maculipinnis*, *P. osborni*, and *Pollimyrus* sp. “*luki*”.

One of the syntypes of *P. nigripinnis* shows some morphological differences from the other syntypes. Six syntypes were collected from Kutu at Lake Mai‐Ndombe, and one was collected from the Uéré River (RMCA P.344: 61.3 mm SL) (Figure 2; also see Supplementary file S1 for hydro‐geographic locations). The latter has a deeper caudal peduncle relative to its body depth (18.3% CPL vs. 15.3%–16.9% CPL), a longer head (27.1% SL vs. 23.5%–25.3% SL), a shorter dorsal fin (18.5% SL vs. 20.9%–23.6% SL), a wider lower jaw (18.8 vs. 15.9%–18.6% HL), and a wider interorbital distance (38.7% HL vs. 31.1%–35.7% HL) compared to the other syntypes of *P. nigripinnis* from Kutu. Its mouth is positioned subterminally, whereas in the Kutu syntypes, it is positioned terminally. Further, it also has fewer dorsal‐fin rays (18 vs. 19–21) and vertebrae (37 vs. 38–40), but more upper‐jaw teeth (10 vs. 8–9) (Table 5). Finally, it is also clearly separated on a PCA of the 26 log‐transformed measurements on PC 3, with the most important loadings being the lower‐jaw width, dorsal‐fin length, and eye diameter (Figure 7). This specimen is, however, not as well preserved as the other syntypes. Even though it is possible that this specimen is simply badly preserved, some characteristics indicate that the syntype series of *P. nigripinnis* is polyspecific. This idea is further supported by the wide hydro‐geographical separation of Kutu and Uéré. Thus, syntype RMCA P.344 is further referred to as *P. nigripinnis* “Uéré,” given the clear differences in morphology from the other syntypes. Although *P. osborni* has been described from the Uele River, of which the Uéré River is an affluent, *P. nigripinnis* “Uéré” clearly differs from the holotype of *P. osborni* by its lower number of circumpeduncular scales (13 vs. 17 in *P. osborni*) and in, for example, head width (49.3% HL vs. 57.5% HL in *P. osborni*) (also see Tables 6 and 7). Thus, both these two specimens are clearly not conspecific despite occurring in the same (sub)basin. It is currently unclear if *P. nigripinnis* “Uéré” could represent a species new to science due to the lack of similar specimens from the same locality or (sub)basin.

**TABLE 6:** Measurements of all *Pollimyrus* species identified as valid in this study, listed alphabetically per morphological group.

Morphological group	Thick‐tailed group													
Species	*Pollimyrus brevis*			*Pollimyrus castelnaui*			*Pollimyrus guttatus*				*Pollimyrus marianne*			
	Syntype (*n* = 2)			Syntype (*n* = 2)			Holotype	Paratype (*n* = 3)		All	Holotype	Paratype (*n* = 10)		All
	Min	Max	Mean	Min	Max	Mean		Min	Max	Mean		Min	Max	Mean
Standard length	50.2	54.0	52.1	58.3	62.5	60.4	39.5	36.3	39.2	38.0	63.9	57.9	66.6	63.0
Measurements in % SL														
Total length	115.4	117.0	116.2	112.6	113.9	113.2	123.6	115.6	119.6	118.8	113.1	111.4	118.1	113.7
Body depth	29.1	32.1	30.6	29.5	31.2	30.4	31.2	30.7	32.1	31.2	24.3	23.7	27.0	25.1
Pre‐dorsal length	67.4	67.5	67.5	66.0	66.1	66.0	65.3	65.0	66.4	65.4	64.1	61.2	65.5	63.4
Pre‐anal length	62.3	64.5	63.4	60.1	62.4	61.2	65.7	62.2	65.8	64.4	58.0	56.6	59.7	57.9
Pre‐pectoral length	27.2	28.4	27.8	25.9	26.1	26.0	29.0	28.0	29.8	29.0	25.0	24.3	26.4	25.4
Pre‐pelvic length	42.6	45.1	43.8	41.4	42.2	41.8	43.7	43.7	45.7	44.5	40.5	38.9	41.2	40.0
Length of dorsal fin	19.1	20.4	19.8	18.3	19.5	18.9	22.1	19.8	22.0	21.3	18.4	18.3	20.1	18.9
Length of anal fin	24.3	24.4	24.4	23.4	25.3	24.3	27.1	25.0	25.8	25.9	24.5	23.6	26.5	24.5
Length of pectoral fin	20.5	22.9	21.7	20.7	21.0	20.8	26.1	23.2	25.4	24.9	22.2	19.7	23.5	21.5
Length of pelvic fin	11.1	11.3	11.2	9.7	9.7	9.7	10.9	9.5	11.1	10.6	10.8	9.5	11.1	10.6
Posterodorsal distance	35.7	38.3	37.0	38.1	38.8	38.5	42.0	38.9	41.0	40.4	39.9	39.8	43.7	41.2
Pectoral–pelvic distance	16.2	21.4	18.8	17.1	19.1	18.1	17.0	17.9	19.6	18.2	15.7	15.3	17.0	15.9
Caudal peduncle length	15.2	15.2	15.2	16.4	16.9	16.7	18.1	16.2	17.9	17.5	20.5	17.2	19.9	19.0
Caudal peduncle depth	7.1	8.0	7.5	6.8	7.4	7.1	6.8	7.0	7.2	7.0	7.3	6.6	7.4	6.9
Belly length	18.7	20.9	19.8	19.6	20.7	20.1	19.4	18.4	19.0	18.9	17.9	17.0	19.1	18.1
Head length	24.6	24.9	24.7	22.4	23.1	22.7	24.6	23.9	26.4	25.3	22.5	21.5	23.3	22.3
Measurements in % HL														
Head width	58.5	60.2	59.4	53.8	54.1	54.0	43.4	51.2	52.4	49.7	58.6	58.4	61.4	59.8
Snout–posterior side of eye	44.3	44.7	44.5	42.3	46.1	44.2	48.5	42.1	46.5	45.3	39.5	41.4	45.8	42.9
Snout–center of eye	34.4	38.0	36.2	33.0	36.3	34.6	34.8	28.3	33.2	31.7	29.0	31.9	36.3	33.6
Internasal length	9.7	10.9	10.3	10.1	10.1	10.1	11.1	8.9	9.6	9.7	8.3	8.6	10.2	9.3
Eye diameter	18.8	19.0	18.9	18.6	19.9	19.2	25.1	22.1	27.3	24.6	18.0	17.7	21.9	19.8
Lower‐jaw width	23.5	25.7	24.6	21.7	22.1	21.9	14.2	15.5	17.3	15.9	18.8	14.6	18.0	16.6
Upper‐jaw width	19.1	22.2	20.7	18.0	19.4	18.7	14.2	13.9	15.6	14.5	18.2	16.8	19.8	18.2
Snout length	14.3	15.1	14.7	11.9	13.4	12.7	13.7	12.1	13.3	13.1	14.7	12.9	15.8	14.0
Interorbital width	39.2	44.6	41.9	38.1	41.5	39.8	39.5	33.9	43.5	39.7	32.2	32.5	40.5	36.8
Postorbital length	61.3	63.5	62.4	60.3	63.0	61.6	56.6	55.8	58.7	56.8	62.8	53.3	62.1	60.5
Measurements (other %)														
Caudal peduncle depth (% BD)	22.2	27.4	24.8	22.9	23.6	23.2	21.7	22.0	23.3	22.4	30.1	24.4	29.3	27.5
Caudal peduncle depth (% CPL)	46.8	52.5	49.7	41.2	43.5	42.4	37.3	39.4	40.0	38.9	35.8	33.4	39.6	36.4
Anal‐fin length (% LD)	119.2	127.7	123.5	127.7	130.0	128.9	123.1	116.7	130.3	121.7	133.4	121.7	140.1	129.6

Morphological groups	Thick‐tailed group											
Species	*Pollimyrus petricolus*			*Pollimyrus stappersii kapangae*	*Pollimyrus stappersii stappersii*		*Pollimyrus krameri* sp. nov.			*Pollimyrus weyli* sp. nov.		
	Holotype	Paratype (*n* = 3)		All	Holotype	Holotype	Holotype	Paratype (*n* = 1)	All	Holotype	Paratype (*n* = 1)	All
		Min	Max	Mean					Mean			Mean
Standard length	87.9	50.3	85.1	69.0	61.6	59.3	45.6	48.7	47.1	51.4	49.1	50.3
Measurements in % SL												
Total length	109.7	113.6	114.9	113.1	112.7	116.6	112.9	117.4	115.1	110.3	115.1	112.7
Body depth	22.2	22.5	25.7	23.6	27.9	27.1	26.7	25.3	26.0	29.6	29.9	29.7
Pre‐dorsal length	60.3	61.5	64.0	62.1	66.9	68.8	66.8	67.9	67.3	66.8	70.4	68.6
Pre‐anal length	58.5	59.6	61.6	60.1	61.6	63.5	60.6	62.2	61.4	59.4	62.6	61.0
Pre‐pectoral length	24.0	23.6	27.3	25.5	26.3	26.8	26.6	26.6	26.6	26.4	27.3	26.8
Pre‐pelvic length	38.4	39.7	43.3	41.2	41.8	43.5	40.7	43.2	41.9	42.0	45.1	43.5
Length of dorsal fin	24.9	21.4	24.0	23.5	20.2	17.3	17.3	17.5	17.4	18.8	18.1	18.4
Length of anal fin	24.3	21.3	23.6	23.0	24.8	22.9	24.5	23.1	23.8	22.5	20.2	21.4
Length of pectoral fin	15.5	18.5	19.2	18.0	20.6	19.9	20.5	19.2	19.9	18.1	19.6	18.8
Length of pelvic fin	8.6	9.5	10.8	9.9	10.9	10.6	10.8	10.5	10.7	9.7	10.8	10.2
Posterodorsal distance	41.8	38.7	41.2	40.3	38.7	37.5	37.0	36.8	36.9	37.6	37.6	37.6
Pectoral–pelvic distance	16.4	17.1	18.5	17.4	17.0	18.9	16.7	18.5	17.6	18.5	20.3	19.4
Caudal peduncle length	15.1	15.4	16.2	15.7	16.7	17.9	16.8	18.0	17.4	16.7	18.0	17.4
Caudal peduncle depth	6.4	5.7	6.8	6.4	6.1	5.9	7.9	7.9	7.9	6.9	7.1	7.0
Belly length	20.4	18.5	22.1	19.9	20.8	21.6	19.5	19.7	19.6	18.2	17.5	17.8
Head length	22.1	22.1	25.8	23.9	23.7	23.8	24.8	25.2	25.0	23.5	25.5	24.5
Measurements in % HL												
Head width	47.6	45.3	47.0	46.6	54.0	52.7	53.8	52.7	53.2	58.7	55.2	56.9
Snout–posterior side of eye	43.6	42.4	45.9	43.6	44.3	44.5	39.7	39.4	39.6	43.5	39.6	41.6
Snout–center of eye	33.2	31.6	33.0	32.7	35.1	34.3	31.8	32.2	32.0	36.5	33.1	34.8
Internasal length	7.5	7.4	8.1	7.7	9.8	9.4	8.2	9.1	8.7	11.0	9.6	10.3
Eye diameter	21.6	22.4	23.7	22.8	18.0	16.9	15.5	14.3	14.9	18.9	17.1	18.0
Lower‐jaw width	15.1	13.8	16.1	15.0	17.9	18.3	14.7	16.2	15.4	19.1	17.1	18.1
Upper‐jaw width	15.9	14.8	18.2	16.7	16.6	18.2	19.7	19.5	19.6	24.5	22.6	23.5
Snout length	13.9	11.9	13.4	12.8	14.0	12.3	13.5	13.5	13.5	14.7	13.7	14.2
Interorbital width	26.6	25.3	33.1	29.3	40.2	37.6	31.7	33.1	32.4	40.9	37.2	39.1
Postorbital length	58.0	55.3	58.5	56.8	61.5	62.3	64.2	60.9	62.6	60.5	62.5	61.5
Measurements (other %)												
Caudal peduncle depth (% BD)	28.8	25.4	27.9	27.1	21.8	21.9	23.4	23.4	23.4	23.4	23.8	23.6
Caudal peduncle depth (% CPL)	42.3	37.2	42.1	40.8	33.2	36.5	47.3	43.7	45.5	41.4	39.6	40.5
Anal‐fin length (% LD)	97.4	95.7	99.6	97.8	132.1	122.7	141.6	132.0	136.8	120.0	111.6	115.8

Morphological group	Slender‐tailed group										
Species	*Pollimyrus adspersus*			*Pollimyrus maculipinnis*			*Pollimyrus osborni*	*Pollimyrus pedunculatus*	*Cyphomyrus plagiostoma*		
	Syntype (*n* = 2)			Holotype	Paratype (*n* = 1)	All	Holotype	Holotype	Syntype (*n* = 2)		
	Min	Max	Mean			Mean			Min	Max	Mean
Standard length	57.9	58.9	58.4	52.9	53.9	53.4	53.0	67.7	88.4	100.2	94.3
Measurements in % SL											
Total length	116.6	119.7	118.2	109.9	117.4	113.6	118.2	118.4	119.2	120.5	119.9
Body depth	30.2	31.7	30.9	30.6	31.5	31.1	33.9	27.0	31.8	34.6	33.2
Pre‐dorsal length	63.4	63.9	63.6	64.6	65.0	64.8	64.3	58.6	54.7	58.5	56.6
Pre‐anal length	55.0	57.0	56.0	56.6	60.0	58.3	58.3	52.1	55.7	57.8	56.8
Pre‐pectoral length	24.5	25.9	25.2	26.9	26.3	26.6	25.4	21.0	23.5	24.8	24.2
Pre‐pelvic length	36.0	36.7	36.4	41.1	42.7	41.9	42.2	34.3	36.3	37.6	37.0
Length of dorsal fin	21.9	24.1	23.0	19.0	19.5	19.3	21.1	25.0	34.4	37.9	36.1
Length of anal fin	28.7	30.4	29.5	30.2	28.2	29.2	28.9	28.9	26.5	27.8	27.1
Length of pectoral fin	22.8	24.6	23.7	19.8	22.0	20.9	23.1	20.9	20.8	22.3	21.5
Length of pelvic fin	10.0	10.6	10.3	10.6	10.5	10.5	10.8	7.0	12.5	13.4	12.9
Posterodorsal distance	42.1	44.3	43.2	41.0	41.6	41.3	41.8	46.9	51.4	52.8	52.1
Pectoral–pelvic distance	16.7	17.0	16.8	17.5	19.3	18.4	18.4	16.5	16.7	17.0	16.9
Caudal peduncle length	17.8	18.1	17.9	18.8	16.9	17.9	19.3	22.8	18.8	21.7	20.2
Caudal peduncle depth	5.9	6.0	5.9	6.0	6.4	6.2	6.1	4.7	4.2	4.8	4.5
Belly length	16.9	19.2	18.1	16.1	18.9	17.5	18.5	15.4	19.9	20.5	20.2
Head length	23.2	23.5	23.3	25.3	25.3	25.3	23.7	20.1	21.4	22.2	21.8
Measurements in % HL											
Head width	51.1	51.9	51.5	55.8	49.5	52.6	57.5	49.4	47.7	54.5	51.1
Snout–posterior side of eye	45.9	49.8	47.8	49.3	46.5	47.9	48.8	44.4	43.8	45.4	44.6
Snout–center of eye	34.5	37.7	36.1	36.6	33.7	35.2	37.7	32.6	35.7	36.7	36.2
Internasal length	8.8	9.5	9.2	7.7	6.8	7.3	9.8	9.2	7.2	7.8	7.5
Eye diameter	25.0	27.6	26.3	28.7	27.1	27.9	27.5	19.3	19.1	19.3	19.2
Lower‐jaw width	15.1	16.6	15.8	18.2	17.3	17.8	18.3	16.5	16.6	16.8	16.7
Upper‐jaw width	15.1	16.0	15.6	17.7	14.7	16.2	14.4	18.3	13.9	18.3	16.1
Snout length	10.5	11.8	11.1	14.4	10.9	12.7	14.4	13.0	18.7	22.0	20.4
Interorbital width	36.9	40.8	38.9	41.2	39.0	40.1	43.8	40.0	37.0	37.3	37.1
Postorbital length	56.8	59.5	58.1	58.5	57.1	57.8	53.6	56.9	53.5	56.9	55.2
Measurements (other %)											
Caudal peduncle depth (% BD)	18.9	19.5	19.2	19.4	20.2	19.8	18.1	17.3	13.3	13.9	13.6
Caudal peduncle depth (% CPL)	33.1	33.2	33.2	31.7	37.6	34.7	31.8	20.6	19.5	25.6	22.5
Anal‐fin length (% LD)	118.8	139.0	128.9	158.6	144.5	151.6	136.7	115.8	73.3	76.9	75.1

Morphological group	Slender‐tailed group										
Species	*Pollimyrus pulverulentus*			*Pollimyrus ichreyeni*				*Pollimyrus ibalazambai* sp. nov.			
	Syntype (*n* = 16)			Holotype	Paratype (*n* = 3)		All	Holotype	Paratype (*n* = 20)		All
	Min	Max	Mean		Min	Max	Mean		Min	Max	Mean
Standard length	61.3	114.3	82.1	70.5	71.3	75.8	72.7	70.2	64.6	79.3	70.2
Measurements in % SL											
Total length	114.0	128.3	118.5	112.7	108.9	113.7	111.8	118.5	113.3	121.1	117.2
Body depth	28.2	35.8	31.1	31.1	29.2	30.2	30.1	33.6	27.4	34.4	31.5
Pre‐dorsal length	61.8	67.3	65.2	65.7	60.0	66.1	64.2	62.3	60.3	65.3	62.1
Pre‐anal length	57.2	64.9	60.8	52.7	53.4	56.8	54.3	56.7	55.5	59.1	57.1
Pre‐pectoral length	26.6	30.2	28.9	25.4	23.8	25.7	25.0	24.9	22.5	25.6	24.0
Pre‐pelvic length	37.4	42.9	40.5	36.6	36.0	37.5	36.7	39.7	37.0	40.7	38.9
Length of dorsal fin	18.5	25.4	22.9	15.2	15.4	16.2	15.7	20.5	20.4	23.9	22.1
Length of anal fin	27.1	30.8	28.8	35.3	34.7	35.4	35.2	30.0	28.1	32.1	29.7
Length of pectoral fin	23.2	26.7	25.0	22.1	22.7	26.2	23.6	25.6	23.1	27.5	25.3
Length of pelvic fin	9.9	12.4	11.4	10.0	9.3	10.3	9.9	9.5	8.4	11.1	9.5
Posterodorsal distance	38.7	42.9	40.6	39.7	40.0	42.6	40.7	43.4	40.3	45.9	43.2
Pectoral–pelvic distance	14.0	17.6	15.5	13.4	13.4	14.9	14.0	18.5	16.1	18.6	17.5
Caudal peduncle length	13.6	18.1	16.0	15.1	16.2	17.8	16.3	17.4	16.6	19.5	18.4
Caudal peduncle depth	3.9	5.4	4.7	4.5	4.3	4.8	4.5	5.4	4.9	6.0	5.5
Belly length	18.1	24.7	21.5	18.0	17.5	20.7	18.9	18.1	17.3	19.9	18.5
Head length	23.5	27.7	25.7	21.5	20.0	20.5	20.6	22.1	20.9	22.8	21.8
Measurements in % HL											
Head width	47.5	54.7	50.4	50.8	51.7	58.1	53.2	55.7	48.3	57.0	53.9
Snout–posterior side of eye	44.7	53.8	48.3	46.5	46.5	49.7	47.5	44.7	43.7	48.3	46.2
Snout–center of eye	32.4	43.8	37.0	35.7	37.0	38.3	37.1	36.6	32.3	38.1	35.3
Internasal length	6.8	9.1	8.1	7.6	7.8	8.9	8.3	8.8	8.1	10.1	8.9
Eye diameter	17.5	25.3	22.8	22.3	24.2	26.0	24.2	26.0	23.8	27.3	25.3
Lower‐jaw width	14.2	20.8	17.2	16.4	14.6	16.9	15.9	14.7	13.9	16.8	15.4
Upper‐jaw width	15.0	21.5	17.6	14.9	14.7	15.7	15.2	17.2	15.7	19.9	17.4
Snout length	9.8	19.9	16.3	14.7	14.9	15.6	15.2	16.0	11.4	16.7	14.6
Interorbital width	29.8	39.1	34.7	37.0	34.2	43.6	38.5	40.5	36.2	46.8	39.5
Postorbital length	49.7	58.0	55.1	56.2	53.2	62.1	57.0	55.3	54.5	58.2	56.5
Measurements (other %)											
Caudal peduncle depth (% BD)	12.7	18.3	15.3	14.4	14.9	15.7	15.0	16.1	15.3	19.2	17.4
Caudal peduncle depth (% CPL)	26.6	32.5	29.5	29.7	24.4	29.4	27.9	31.1	27.3	33.1	29.7
Anal‐fin length (% LD)	120.0	126.7	122.9	233.1	213.8	229.4	224.9	146.1	121.9	146.8	134.3

Morphological group	*Pollimyrus tumifrons* group			*Pollimyrus isidori* group					*Pollimyrus vanneeri* sp. nov.			
Species	*P. tumifrons*	*Pollimyrus aequipinnis*	*Pollimyrus anterodorsalis*	*Pollimyrus fasciaticeps*	*P. isidori*	*Pollimyrus nigricans*			*P. vanneeri* sp. nov.			
	Holotype	Holotype	Holotype	Holotype	Holotype	Syntype (*n* = 9)			Holotype	Paratype (*n* = 17)		All
						Min	Max	Mean		Min	Max	Mean
Standard length	88.6	90.1	44.8	49.3	84.3	76.9	86.8	80.2	100.9	65.6	123.6	91.9
Measurements in % SL												
Total length	115.9	116.0	121.3	116.6	116.2	113.2	116.5	114.4	116.2	108.0	120.1	116.9
Body depth	28.7	27.5	29.3	28.3	31.9	23.9	31.1	28.0	26.3	22.6	28.7	26.1
Pre‐dorsal length	60.3	62.0	60.4	61.6	63.8	64.5	67.8	66.0	62.3	56.4	65.0	61.2
Pre‐anal length	61.3	58.6	61.4	58.9	62.7	59.2	62.5	61.2	60.3	52.4	58.8	56.9
Pre‐pectoral length	26.1	26.1	28.1	27.3	25.0	26.0	27.5	26.7	26.5	22.5	26.8	25.4
Pre‐pelvic length	40.1	38.6	43.9	40.9	44.2	39.8	45.0	42.5	39.8	33.7	40.2	37.9
Length of dorsal fin	29.3	30.6	29.3	22.3	22.5	16.0	19.1	17.7	22.5	22.1	25.1	23.4
Length of anal fin	26.4	28.2	26.4	27.7	26.8	21.0	24.6	22.8	26.7	24.0	30.2	27.3
Length of pectoral fin	21.2	26.2	22.6	21.3	20.7	21.1	22.5	21.9	21.7	17.7	22.7	20.9
Length of pelvic fin	11.2	12.4	12.7	9.9	10.6	8.9	10.4	9.8	10.8	8.6	11.3	10.5
Posterodorsal distance	43.2	45.8	46.4	43.5	42.8	35.5	38.7	37.5	40.1	39.9	45.3	42.7
Pectoral–pelvic distance	16.4	15.8	16.0	16.9	21.4	16.4	21.0	18.8	16.3	14.2	17.6	15.6
Caudal peduncle length	17.8	17.3	18.5	20.7	19.1	15.6	20.0	17.5	18.5	15.6	19.5	18.1
Caudal peduncle depth	4.5	4.9	4.7	7.2	7.4	6.9	8.1	7.4	5.6	5.2	6.5	5.9
Belly length	20.8	19.6	18.5	17.4	19.2	17.9	20.7	19.4	21.1	17.5	20.6	19.2
Head length	24.3	24.0	26.6	25.2	23.3	23.7	25.6	24.5	25.0	22.5	25.7	24.1
Measurements in % HL												
Head width	49.3	53.5	50.8	59.7	50.9	51.2	57.3	54.2	45.5	40.0	54.1	47.7
Snout–posterior side of eye	45.1	47.0	50.0	50.0	41.4	37.5	42.6	40.9	43.7	40.4	45.4	43.5
Snout–center of eye	37.7	34.3	36.9	37.0	32.9	30.3	34.1	32.6	33.9	29.0	37.2	34.1
Internasal length	7.7	8.8	7.8	7.2	8.3	7.1	9.0	8.0	8.0	7.0	9.6	7.8
Eye diameter	18.2	20.1	23.7	26.8	22.9	15.3	19.0	16.5	18.9	13.7	21.6	19.4
Lower‐jaw width	18.6	14.8	18.9	16.9	16.9	17.4	20.9	19.5	13.6	12.2	15.7	14.1
Upper‐jaw width	17.5	13.5	16.9	14.2	16.3	15.2	21.2	18.5	14.0	12.7	17.0	15.0
Snout length	20.3	17.0	18.3	13.2	16.7	12.6	14.9	13.8	17.9	15.3	21.0	17.9
Interorbital width	41.0	41.5	42.9	41.4	36.6	24.9	38.0	31.4	33.3	26.9	34.4	29.8
Postorbital length	54.0	53.9	53.3	52.2	57.3	62.4	67.3	64.5	56.6	52.7	59.9	57.2
Measurements (other %)												
Caudal peduncle depth (% BD)	15.6	17.9	16.0	25.5	23.3	23.2	30.4	26.5	21.5	20.3	24.2	22.4
Caudal peduncle depth (% CPL)	25.1	28.6	25.3	34.9	38.9	38.3	47.60	42.30	30.4	28.0	33.6	32.6
Anal‐fin length (% LD)	90.0	92.2	90.1	124.1	119.4	121.0	145.0	129.2	118.5	107.3	131.3	116.8

*Note*: See Tables S9–S12 for additional data on synonymized species.

Abbreviations: BD, body depth; CPL, caudal peduncle length; HL, head length; LD, length of dorsal fin; SL, standard length.

**TABLE 7:** Meristics of all *Pollimyrus* species as valid in this study, listed alphabetically per morphological group.

Morphological group	Thick‐tailed group									
Species	*Pollimyrus brevis*		*Pollimyrus castelnaui*		*Pollimyrus guttatus*			*Pollimyrus marianne*		
	Syntype (*n* = 2)		Syntype (*n* = 2)		Holotype	Paratype (*n* = 3)		Holotype	Paratype (*n* = 10)	
	Min	Max	Min	Max		Min	Max		Min	Max
Dorsal‐fin rays	18	18	16	17	19	18	18	16	15	17
Anal‐fin rays	23	25	22	22	22	22	23	23	22	23
Pectoral‐fin rays	10	10	10	10	10	10	10	9	9	10
Pelvic‐fin rays	6	6	6	6	6	6	6	6	6	9
Vertebra	39	39	38	38	38	37	38	39	38	39
Teeth in upper jaw	7	8	7	7	7	6	7	7	5	8
Teeth in lower jaw	7	7	8	9	8	7	8	8	6	8
Lateral‐line scales	40	46	47	47	52	43	51	48	48	54
Circumpeduncular scales	11	13	12	13	16	15	17	16	14	17
Scales lateral line–anal‐fin origin	9	11	10	11	10	11	13	10	10	12
Scales lateral line–dorsal‐fin origin	8	9	10	10	10	10	12	9 or 10	9	11
Scales lateral line–pelvic‐fin origin	10	10	13	13	12	14	14	10	11	13

Morphological groups	Thick‐tailed group								
Species	*Pollimyrus petricolus*			*Pollimyrus stappersii stappersii*	*Pollimyrus stappersii kapangae*	*Pollimyrus krameri* sp. nov.		*Pollimyrus weyli* sp. nov.	
	Holotype	Paratype (*n* = 3)		Holotype	Holotype	Holotype	Paratype (*n* = 1)	Holotype	Paratype (*n* = 1)
		Min	Max						
Dorsal‐fin rays	21	19	22	18	16	15	15	17	17
Anal‐fin rays	24	22	25	22	26	21	24	23	21
Pectoral‐fin rays	10	10	10	10	9	9	9	9	9
Pelvic‐fin rays	6	6	6	6	6	6	6	6	6
Vertebra	42	41	43	39	38	37	38	39	38
Teeth in upper jaw	7	7	7	7	5	7	7	7	7
Teeth in lower jaw	8	8	8	8	8	8	9	9	9
Lateral‐line scales	62	58	67	53	48	46	53	?	?
Circumpeduncular scales	17	16	20	13	13	13	14	14	12
Scales lateral line–anal‐fin origin	13	13	14	13	12	9	12	?	11?
Scales lateral line–dorsal‐fin origin	14	13	13	10	10	9	9	10	11?
Scales lateral line–pelvic‐fin origin	14	14	15	14	12	11	11	13?	?

Morphological group	Slender‐tailed group						
Species	*Pollimyrus adspersus*		*Pollimyrus maculipinnis*		*Pollimyrus osborni*	*Pollimyrus pedunculatus*	*Cyphomyrus plagiostoma*	
	Syntype (*n* = 2)		Holotype	Paratype (*n* = 1)	Holotype	Holotype	Syntype (*n* = 2)	
							Min	Max
Dorsal‐fin rays	19	20	17	17	16	19	32	33
Anal‐fin rays	27	27	24	24	24	24	27	29
Pectoral‐fin rays	9	10	10	10	10	10	12	12
Pelvic‐fin rays	6	6	6	6	6	?	6	6
Vertebra	37	40	38	38	37	40	44	44
Teeth in upper jaw	7	8	7	7	7	7	9	9
Teeth in lower jaw	8	8	9	9	9	7	8	10
Lateral‐line scales	47	47	62	51	51	56	69	73
Circumpeduncular scales	?	?	15	16	17	16	14	14
Scales lateral line–anal‐fin origin	11	12	14	14	12	11	14	17
Scales lateral line–dorsal‐fin origin	11	13	14	15	15	11	14	17
Scales lateral line–pelvic‐fin origin	12	13	16	17	15	13	17	18

Morphological group	Slender‐tailed group							
Species	*Pollimyrus pulverulentus*		*Pollimyrus schreyeni*			*Pollimyrus ibalazambai* sp. nov.		
	Syntype (*n* = 16)		Holotype	Paratype (*n* = 3)		Holotype	Paratype (*n* = 20)	
	Min	Max		Min	Max		Min	Max
Dorsal‐fin rays	18	24	17	17	17	19	17	21
Anal‐fin rays	25	28	31	30	32	26	25	28
Pectoral‐fin rays	9	10	10	10	10	10	10	10
Pelvic‐fin rays	6	6	6	6	6	6	6	6
Vertebra	37	40	39	40	41	40	38	41
Teeth in upper jaw	8	10	7	7	7	7	6	8
Teeth in lower jaw	9	11	8	7	9	9	7	9
Lateral‐line scales	42	54	52	51	58	55	50	59
Circumpeduncular scales	11	16	12	12	13	13	12	15
Scales lateral line–anal‐fin origin	10	14	10	9	11	12	11	15
Scales lateral line–dorsal‐fin origin	10	13	10	10	11	12	11	14
Scales lateral line–pelvic‐fin origin	12	18	11	9	10	14	11	16

Morphological group	*Pollimyrus tumifrons* group			*Pollimyrus isidori* group				*Pollimyrus vanneeri* sp. nov.		
Species	*P. tumifrons*	*Pollimyrus aequipinnis*	*Pollimyrus anterodorsalis*	*Pollimyrus fasciaticeps*	*P. isidori*	*Pollimyrus nigricans*		*P. vanneeri* sp. nov.		
	Holotype	Holotype	Holotype	Holotype	Holotype	Syntype (*n* = 9)		Holotype	Paratype (*n* = 17)	
						Min	Max		Min	Max
Dorsal‐fin rays	28	25	24	17	20	14	17	23	21	24
Anal‐fin rays	26	27	24	24	24	22	24	26	24	27
Pectoral‐fin rays	12	12	12	10	10	10	10	11	10	12
Pelvic‐fin rays	6	6	6	6	6	6	6	6	6	6
Vertebra	42	41	40	37	38	37	39	41	41	43
Teeth in upper jaw	9	8	7	7	?	6	8	7	6	8
Teeth in lower jaw	10	10	8	8	?	8	9	7	5	9
Lateral‐line scales	62	67	63	52	51	48	55	72	59	73
Circumpeduncular scales	16	18	13	18	16	17	20	19	17	23
Scales lateral line–anal‐fin origin	15	15	15	14	14	11	14	18	12	18
Scales lateral line–dorsal‐fin origin	16	16	16	16	14	11	14	18	13	20
Scales lateral line–pelvic‐fin origin	17	17	15	14	16	12	12	19	14	20

*Note*: See Tables S9–S12 for additional data on synonymized species.

*Pollimyrus osborni* (Nichols and Griscom, 1917) (Figure S17) is only known from the holotype. The species differs from most slender‐tailed species by the aforementioned characteristics for each of the species already differentiated in the complex. Further, it can be distinguished from *P. adspersus* by having a wider head length (57.5% HL vs. 51.1%–51.9% HL), a longer pre‐ventral distance (42.2% SL vs. 36.0%–36.7% SL), and fewer dorsal‐ (17 vs. 19–20) and anal‐fin rays (24 vs. 27). Finally, it differs from *P*. sp. “luki” by having more circumpeduncular scales (17 vs. 12–15) and relatively long and broad teeth that are packed closely together (vs. teeth that are smaller and have an open space between them).

Specimens found in the Luki River in the Democratic Republic of the Congo, here preliminarily identified as *Pollimyrus* sp. “*luki*,” differ from all previously mentioned slender‐tailed species as described earlier. They have overlapping ranges of all measurements with *P. adspersus* (Table 6). No differences in meristics could be found (Table 7). Nevertheless, the teeth of these specimens are small, whereas the teeth of *P. adspersus* are larger and take in more space in the upper jaw. In the lower jaw, the teeth of these specimens are more embedded and have mostly symmetrical cusps, whereas the teeth of *P. adspersus* are asymmetrical, with one cusp being larger than the other. In the upper jaw, all specimens found in the Luki River have a type 1 dentition (Figure 1e), whereas the type specimens of *P. adspersus* have either a type 2 or have an additional tooth behind the most anterior tooth. As such, the specimens found in the Luki River can be regarded as separate species based on their well‐distinct dentition. Furthermore, the species probably differ in their known geographic occurrence, with the syntypes of *P. adspersus* having been reported from “West Africa” (Günther, 1866). As these specimens are morphologically distinct from all other *Pollimyrus*, they are proposed as a new species to science: *Pollimyrus ibalazambai* sp. nov. (Figure 15). The detailed description of this species new to science is provided below.

*Pollimyrus adspersus* (Günther, 1866) (Figure S18) differs from all other slender‐tailed *Pollimyrus* as mentioned earlier.

#### 3.2.5 *P. tumifrons* group

*P. tumifrons* (Boulenger, 1902) (Figure S19) and its two junior synonyms, *P. aequipinnis* (Pellegrin, 1924) and *P. anterodorsalis* (David & Poll, 1937) differ from the other *Pollimyrus* species (except *C. plagiostoma*) by having an anal fin that is slightly shorter than the dorsal fin, whereas in the other species it is about equal or longer than the dorsal fin (90.0%–92.2% LD vs. 95.7%–223.1% LD). The dorsal fin is longer in *P. tumifrons* than in other *Pollimyrus* species (29.3%–30.6% SL vs. 15.2%–25.4% SL) and shorter than in *C. plagiostoma* (29.3%–30.6% SL vs. 34.4%–37.9% SL). The mouth is clearly placed inferior in *P. tumifrons*, whereas it is rather subinferior to terminal in other *Pollimyrus* species. The posterior edge of the mouth is positioned beneath the eye in *P. tumifrons*, whereas it is never positioned under the eye in other *Pollimyrus* species. They also have, although only slightly, more pectoral‐fin rays (12 vs. 9–11) than most other *Pollimyrus* specimens (except one specimen of *P*. sp. “*kouilou‐niari*” and most specimens of *C. plagiostoma*). The anterior nostril is positioned lower than the posterior one in *P. tumifrons*, whereas the anterior one is positioned higher than the posterior one in all other *Pollimyrus* species, except *P. pedunculatus*. Although this species complex shows characteristics that are different from most congenerics and is therefore easily differentiated from the other *Pollimyrus* species, it remains unclear to which genus *P. tumifrons* would correspond better than *Pollimyrus*. For this reason, the species is retained within *Pollimyrus*.

*Pollimyrus tumifrons* has been described from the Ubangi River near Banzyville (nowadays: Mobayi‐Mbongo) in north‐west DRC, *P. anterodorsalis* from Panga (David & Poll, 1937) along the Aruwimi in north‐eastern DRC, and *P. aequipinnis* from the Kasaï River at N'Gombe (Pellegrin, 1924) in south‐western DRC (Figure 2; also see Supplementary file S1 for hydro‐geographic locations). *Pollimyrus anterodorsalis* was synonymized with *P. tumifrons*, by Taverne (1971a, 1971b), although, unfortunately, without further explanations. Later on, *P. aequipinnis* was also synonymized with *P. tumifrons* by Poll (1976), based on its overall similar appearance but, unfortunately, also without any further detailed explanation. However, morphologically, the holotype of *P. aequipinnis* has a visually small snout, whereas the holotypes of the other two nominal species have large snouts. Further, *P. aequipinnis* differs from both by having a shorter pre‐pelvic distance (38.5% SL vs. 40.1% SL in *P*. *tumifrons* and 43.9% SL in *P. anterodorsalis*) and more lateral‐line scales (67 vs. 62 in *P*. *tumifrons* and 63 in *P. anterodorsalis*) and more circumpeduncular scales (18 vs. 16 in *P*. *tumifrons* and 13 in *P. anterodorsalis*). These differences would suggest that *P. aequipinnis* can be a species distinct from the other two synonymized species within the *P. tumifrons* group. Furthermore, two studied specimens identified as *P. tumifrons* (RMCA 158002 and 158003) morphologically resemble the type specimens of both *P. tumifrons* and *P. anterodorsalis* in the aforementioned characteristics more than that of *P. aequipinnis*, although they were collected sympatrically with the last one. Indeed, both RMCA specimens originate from the Luachimo River, which is near the type locality of *P. aequipinnis*, the Kasaï River, with the former being an affluent of the latter. However, there are no data for specimens similar to the holotype of *P. aequipinnis*, and no other specimens are available from the type localities of all three synonymized species to confirm the observed differences. Therefore, the alpha‐taxonomic issues within this group cannot be resolved until more specimens become available. We therefore suggest to retain all three synonymized nominal species within a single valid species, *P. tumifrons*, for the time being.

### 3.3 New species descriptions in *Pollimyrus*

Class Actinopterygii Klein, 1885

Order Osteoglossiformes Berg, 1940

Family Mormyridae Bonaparte, 1831

Subfamily Mormyrinae Bonaparte, 1831

Genus *Pollimyrus* Taverne, 1971

*Pollimyrus* Taverne, 1971: 140 (type species: *Mormyrus isidori* Valenciennes, 1847, by original designation).

**Pollimyrus ibalazambai sp. nov**.

Zoobank registration: urn:lsid:zoobank.org:act:EA58D11A‐D2BB‐43E4‐982B‐4A461985C857

**FIGURE 8:**
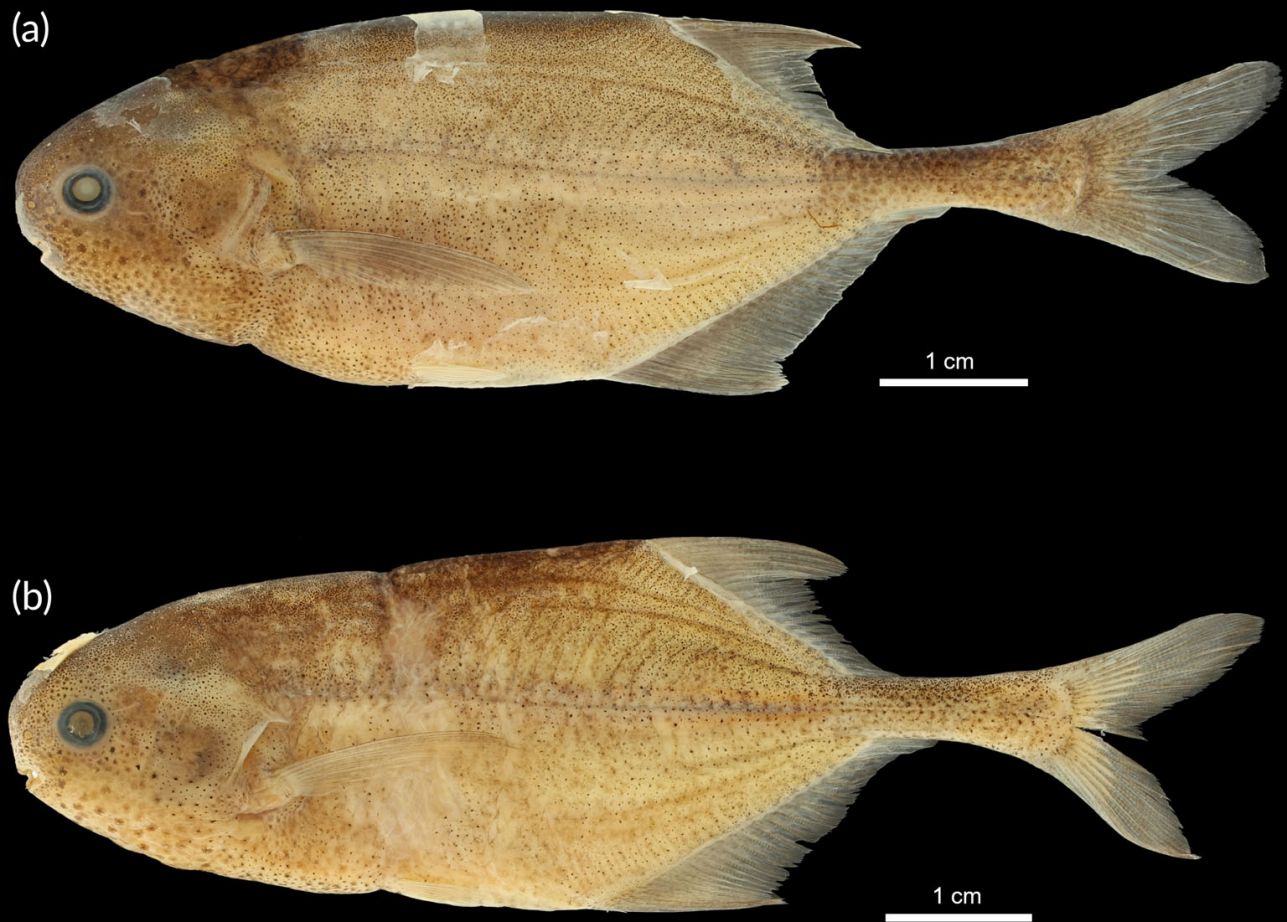
Photographs of preserved specimens of *Pollimyrus ibalazambai* sp. nov. (a) Holotype (Royal Museum for Central Africa [RMCA] 2017.014.P.0002: 70.2 mm SL [standard length]). (b) Paratype (RMCA 2017.014.P.0020‐0021, ID = 22: 70.7 mm SL).

**FIGURE 9:**
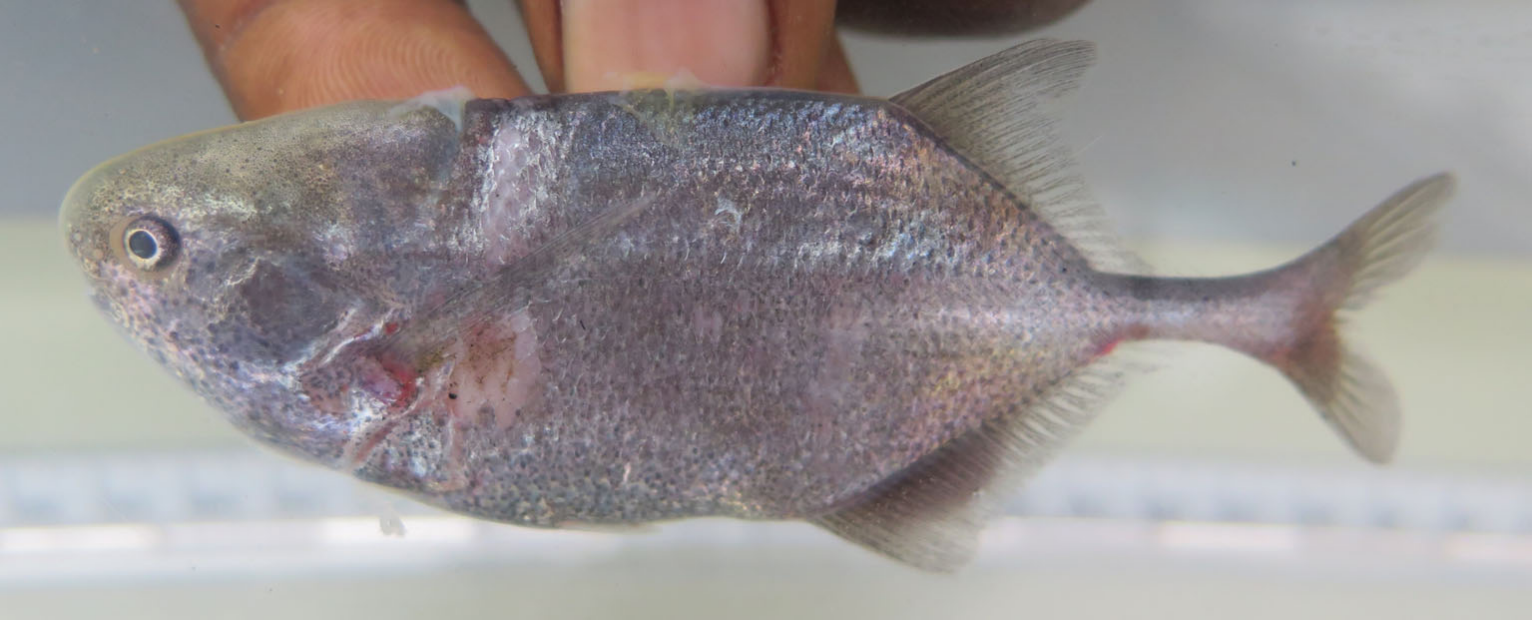
Photograph of a live type specimen of *Pollimyrus ibalazambai* sp. nov. from the Luki River, near the Kimbozi Bridge (by S.W.L., Mbisa‐Congo I, August 10, 2016).

Figures 8 and 9; Tables 6 and 7.

*Pollimyrus* sp. “*luki*”

**Type material**

**Holotype**

**DEMOCRATIC REPUBLIC OF THE CONGO •** Luki River near Kimbozi Bridge; 5°42′3.6″ S, 12°57′35.1″ E; June 24, 2017; S. Wamuini Lukayilakio and Z.J. Kosi leg.; 70.2 mm SL; RMCA 2017.014.P.0002.

**Paratypes**

**DEMOCRATIC REPUBLIC OF THE CONGO •** one paratype; same data as holotype; 76.0 mm SL; RMCA 2017.014.P.0001 **•** three paratypes; same data as holotype; 67.0–79.3 mm SL; RMCA 2017.014.P.0003‐0005 **•** two paratypes; same data as holotype; 67.5–67.7 mm SL; AMNH 281713 **•** two paratypes; same data as holotype; 70.7–71.4 mm SL; RMCA 2017.014.P.0008‐0009 **•** six paratypes; same data as holotype; 64.6–71.9 mm SL; RMCA 2017.014.P.0010‐0015 **•** two paratypes; same data as holotype; 70.1–74.4 mm SL; BMNH 2024.9.20.2‐3 **•** two paratypes; same data as holotype; 69.8–70.2 mm SL; ZSM 49655 **•** two paratypes; same data as holotype; 70.7–77.5 mm SL; RMCA 2017.14.P.0020‐0021.

**Diagnosis**

Distinguished from its congeners by the following unique combination of characters: a slender tail (15.3%–19.2% BD vs. thicker, 21.7%–31.2% BD, in *P. brevis*, *P. castelnaui*, *P. fasciaticeps*, *P. guttatus*, *P. isidori*, *P. krameri*, *P. marianne, P. nigricans*, *P. petricolus*, *P. stappersii*, *P. vanneeri*, and *P. weyli*); a blocky snout (vs. rounded snout in *P. brevis*, *P. castelnaui*, *P. guttatus*, *P. krameri*, *P. marianne, P. petricolus*, *P. stappersii*, *P. vanneeri*, and *P. weyli*); pointed pectoral fin (vs. rounded in *P. brevis*, *P. castelnaui*, *P. guttatus*, *P. krameri*, *P. marianne, P. petricolus*, *P. stappersii*, *P. tumifrons, P. vanneeri*, and *P. weyli*); concave anal and dorsal fins (vs. rounded in *P. brevis*, *P. castelnaui*, *P. guttatus*, *P. krameri*, *P. marianne, P. petricolus*, *P. stappersii*, *P. vanneeri*, and *P. weyli*); absence of chin (vs. obvious chin in *P. brevis*, *P. castelnaui*, *P. guttatus*, *P. krameri*, *P. marianne, P. petricolus*, *P. stappersii*, *P. vanneeri*, and *P. weyli*); small, widely spaced, symmetrical teeth with clear cusps; a short head (20.9%–22.8% SL vs. 23.2%–27.7% SL in *P. adspersus, P. maculipinnis, P. osborni*, and *P. pulverulentus*); a short dorsal fin (20.4%–23.9% SL vs. shorter, 15.2%–19.5% SL, in *P. maculipinnis* and *P. schreyeni*, and longer, 25.0%–37.9% SL, in *P. pedunculatus* and *C. plagiostoma*); and anterior nostril positioned higher than the posterior one (vs. vice versa in *P. pedunculatus*). Also see Supplementary file S1 for details and a differential diagnosis with each species.

**Description**

Based on holotype and paratypes. Measurements and meristics are given in Tables 6 and 7. A relatively small species, with a maximum observed size of 79.3 mm SL. Body oblong or diamond shaped and dorso‐ventrally compressed. Deepest point of body around anal‐fin origin. Head blocky and less deep than body. Mouth small and subterminal, not reaching level of eye. No obvious chin. Anterior nostril positioned higher than posterior one. Posterior nostril close to eye. Nostrils separated by less than half snout–eye distance. Very small, close‐set, bicuspid teeth in a single row, six to eight in the upper jaw and seven to nine in the lower jaw. Arch of teeth not filling entire upper and lower jaw. Most anterior tooth on upper jaw clearly positioned anteriorly of second most teeth left and right of it. Anal‐fin origin slightly in front of dorsal‐fin origin. Anal fin longer than dorsal fin. Anterior parts of anal and dorsal fins higher than posterior part with concave diminution (see Figure 1 for definition). Pectoral fin long, reaching tip of or even extending beyond pelvic fin. First three pectoral‐fin rays longer than others with concave diminution. Caudal‐fin lobes somewhat rounded and long. Long and slender caudal peduncle.

**Color in life**

Body generally light silvery‐grayish with some greenish/olive spots on the head and dorsum and some dark gray spots on the underside of the head. Dorsal, anal, and caudal fins darker gray at base and translucent at distal end. Pectoral fins light to dark gray at base and translucent at distal end. Pelvic fins slightly pinkish and translucent at distal end. Slight darker gray midlateral stripe of one to two scales wide running from behind the head to the caudal peduncle (Figure 9).

**Color in preserved specimens**

Body generally light brownish with large darker brown spots on the head and smaller ones over the entire body. Head slightly darker. Dorsal side of the body and caudal peduncle dark brown. Dorsal, anal, and caudal fins yellow to brown at base and translucent at distal end. Pectoral and pelvic fins light yellow to light brown at base and translucent at distal end. Lateral line gray to black. Some specimens with darker supralateral line(s) (Figure 8).

**Distribution and habitat**

**FIGURE 10:**
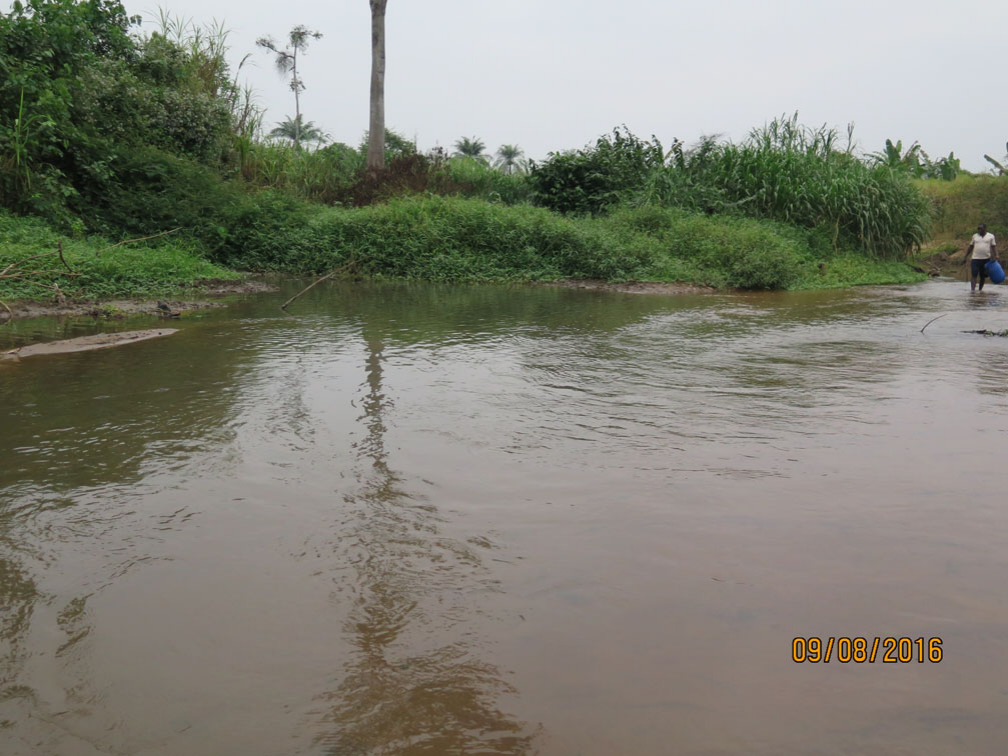
Photograph of the habitat where the type specimens of *Pollimyrus ibalazambai* sp. nov. were caught: Luki River downstream from the Kimbozi Bridge (by S.W.L., MbiSa‐Congo I, August 9, 2016).

The specimens of *P. ibalazambai* were collected near the bridge of Kimbozi Village in the Luki River, a left bank affluent of the Lukunga, itself a right bank affluent of the Lower Congo River (DRC) (Figures 2 and 10). The river where the specimens were caught was about 12 m wide and 60 cm deep. The river flowed at a speed of 0.3 m/s. The banks of the river were grassy, and the canopy above the river covered about 10% of its surface only. The floor of the river consisted of mostly sand with some mud, gravel, and pebbles. The following physicochemical parameters were collected at the type locality: water temperature about 22°C, pH 7.6, salinity 0.1 ppm, and oxygen level of 7.3 mg/L.

Etymology

The specific epithet is a noun honoring Professor Dr. Armel Ibala Zamba (1975–) (Université Marien Ngouabi, the Republic of the Congo) for his contributions to African ichthyology and his work in the Luki River basin (DRC) within the framework of his PhD (2005–2010).

**Pollimyrus krameri sp. nov**.

Zoobank registration: urn:lsid:zoobank.org:act:48E2A84F‐625C‐4891‐A7E8‐F6368805AE1E

**FIGURE 11:**
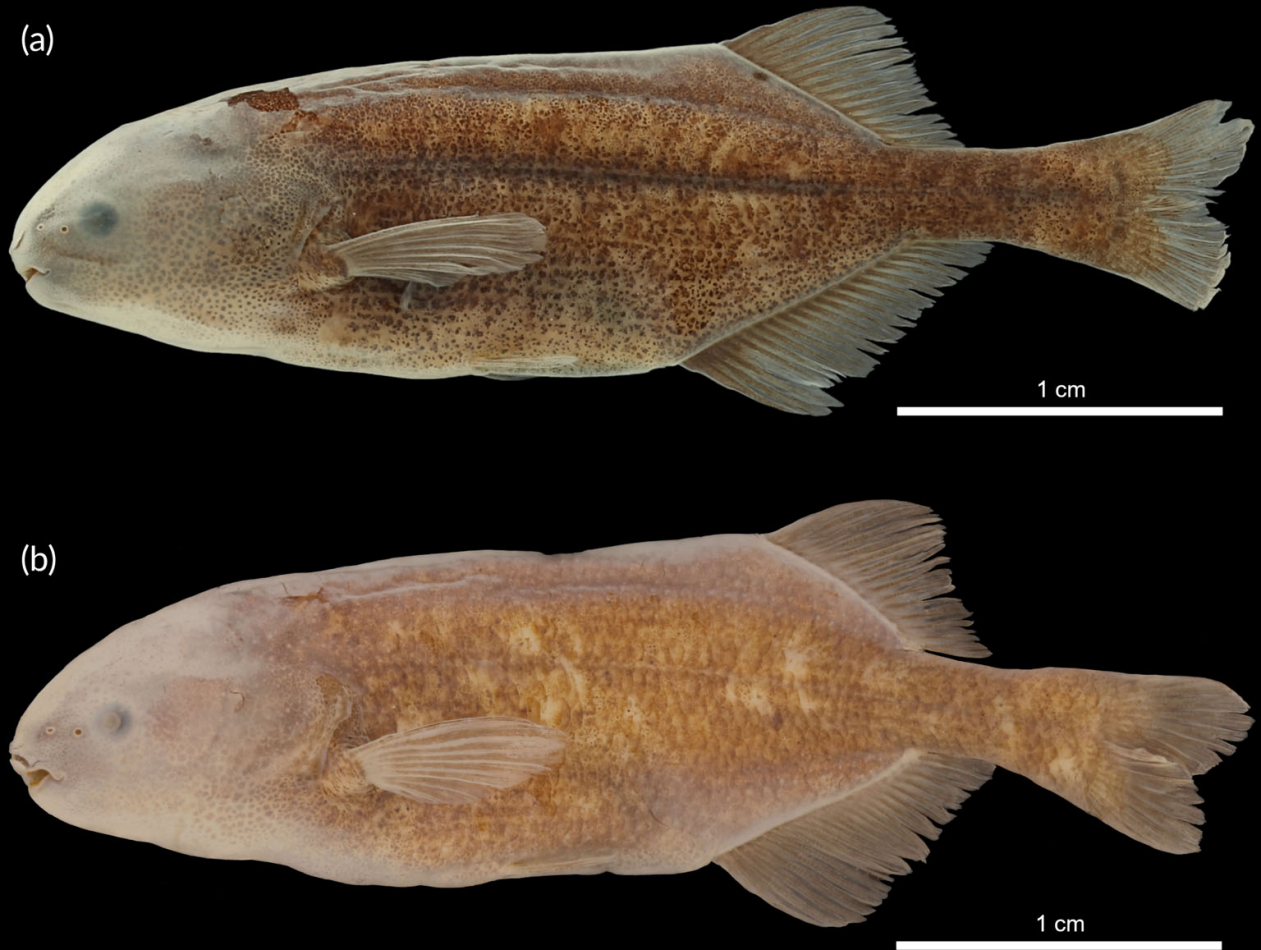
Photographs of preserved specimens of *Pollimyrus krameri* sp. nov. (a) Holotype (South African Institute for Aquatic Biodiversity [SAIAB] 73892: 45.6 mm SL [standard length]). (b) Paratype (SAIAB 237203: 48.7 mm SL).

**FIGURE 12:**
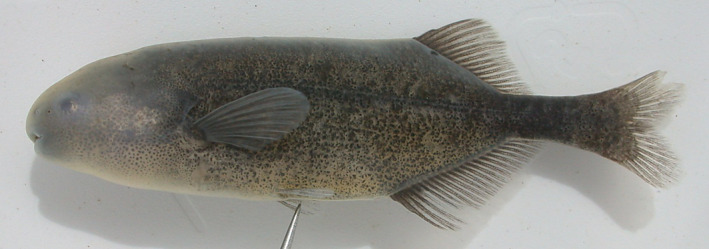
Photograph of dead, but not yet fixed, holotype specimen of *Pollimyrus krameri* sp. nov. (South African Institute for Aquatic Biodiversity [SAIAB] 73892: 45.6 mm SL [standard length]) from the Lugenda River (by R.B., August 22, 2003).

Figures 11 and 12; Tables 6 and 7

*Pollimyrus* sp. “lugenda”

**Type material**

**Holotype**

**MOZAMBIQUE •** Lugenda River near the Mpamanda Fishing camp; 12°26′51″ S, 37°35′45″ E; August 22, 2003; R. Bills leg.; 45.6 mm SL; SAIAB 73892.

**Paratypes**

**MOZAMBIQUE •** one paratype; same data as holotype; 48.7 mm SL; SAIAB 237203.

**Diagnosis**

Distinguished from its congeners by the following unique combination of characters: thick caudal peduncle (23.4%–23.4% BD vs. shallower, 12.7%–21.9% BD, in *P. adspersus*, *P. maculipinnis*, *P. osborni*, *P. pedunculatus*, *P. pulverulentus*, *P. schreyeni*, *P. stappersii*, *P. tumifrons*, *P. ibalazambai*, and *C. plagiostoma*, and deeper, 25.4%–28.8% BD, in *P. petricolus*); rounded pectoral fins (vs. pointed in *P. adspersus, P. fasciaticeps, P. isidori, P. maculipinnis, P. nigricans, P. osborni, P. pedunculatus, P. pulverulentus, P. schreyeni, P. ibalazambai*, and *C. plagiostoma*); rounded (similar height of rays) anterior part of dorsal and anal fins (vs. pointed in *P. adspersus, P. maculipinnis, P. osborni, P. pedunculatus, P. pulverulentus, P. schreyeni, P. tumifrons, P. ibalazambai* sp. nov, and *C. plagiostoma*); small chin (vs. no chin in *P. adspersus, P. fasciaticeps, P. isidori, P. maculipinnis, P. nigricans, P. osborni, P. pedunculatus, P. pulverulentus, P. schreyeni, P. vanneeri, P. ibalazambai*, and *C. plagiostoma*); shallow body (25.3%–26.7% SL vs. thicker, 28.2%–35.8% SL, in *P. adspersus, P. brevis, P. castelnaui, P. fasciaticeps, P. guttatus, P. isidori, P. maculipinnis, P. osborni, P. pedunculatus, P. pulverulentus, P. schreyeni, P. weyli*, and *C. plagiostoma*); short snout (13.5% HL vs. shorter, 10.5%–11.8% HL, in *P. adspersus* and longer, 14.9%–22.0% HL, in *P. isidori, P. schreyeni, P. tumifrons, P. vanneeri*, and *C. plagiostoma*); shorter snout–posterior side eye distance (39.4%–39.7% HL vs. longer, 39.5%–53.8% HL, in *P. adspersus, P. brevis, P. castelnaui, P. fasciaticeps, P. guttatus, P. isidori, P. maculipinnis, P. marianne, P. osborni, P. pedunculatus, P. petricolus, P. pulverulentus, P. schreyeni, P. stappersii, P. tumifrons, P. ibalazambai*, and *C. plagiostoma*); small eye (14.3%–15.5% HL vs. larger, 15.5%–28.7% HL, in *P. adspersus, P. brevis, P. castelnaui, P. fasciaticeps, P. guttatus, P. isidori, P. maculipinnis, P. marianne, P. osborni, P. pedunculatus, P. petricolus, P. pulverulentus, P. schreyeni, P. tumifrons, P. weyli, P. ibalazambai*, and *C. plagiostoma*); short posterodorsal distance (36.8%–37.0% SL vs. longer, 38.1%–52.8% SL, in *P. adspersus, P. castelnaui, P. fasciaticeps, P. guttatus, P. isidori, P. maculipinnis, P. osborni, P. pedunculatus, P. petricolus, P. pulverulentus, P. schreyeni, P. tumifrons, P. vanneeri*, *P. ibalazambai*, and *C. plagiostoma*); long head (24.8%–25.2% SL vs. shorter, 22.4%–23.3% SL, in *P. castelnaui* and *P. isidori*); few dorsal‐fin rays (15 vs. more, 17–33, in *P. adspersus, P. brevis, P. fasciaticeps, P. guttatus, P. isidori, P. maculipinnis, P. pedunculatus, P. petricolus, P. pulverulentus, P. schreyeni, P. tumifrons, P. vanneeri*, *P. weyli, P. ibalazambai*, and *C. plagiostoma*); and few circumpeduncular scales (13–14 vs. more, 16–23, in *P. fasciaticeps, P. isidori, P. nigricans, P. osborni, P. pedunculatus, P. petricolus*, and *P. vanneeri*). Also see Supplementary file S1 for details and a differential diagnosis with each species.

**Description**

Based on holotype and paratype. Measurements and meristics are given in Tables 6 and 7. A relatively small species, with a maximum observed size of 48.7 mm SL. Body oblong, elongated, and dorso‐ventrally compressed. Deepest point of body at anal‐fin origin, and not changing drastically over entire body. Head round and less deep than body. Mouth small and subterminal, not reaching level of eye. Slight chin. Snout protruding. Anterior nostril positioned higher than posterior one. Posterior nostril close to eye. Nostrils separated by less than snout–eye distance. Bicuspid teeth in single row, eight to nine in upper jaw and seven in lower jaw. Arch of teeth filling entire upper and lower jaw. Most anterior tooth on upper jaw clearly positioned anteriorly of second most teeth left and right of it. Anal‐fin origin posterior of dorsal‐fin origin. Anal fin longer than dorsal fin. Anterior parts of anal and dorsal fins slightly higher than posterior part. Anal‐ and dorsal‐fin edges straight. Pectoral fin rounded, reaching halfway pelvic fin. Caudal‐fin lobes rounded and broad, overlapping in middle. Thick caudal peduncle.

**Color in life**

Body dark brown or gray. Ventral side of head and belly light yellowish. Caudal peduncle slightly darker brown or gray. Dorsal, anal, and caudal fins dark brown at base and slightly translucent at distal end. Pectoral fins dark brown or gray and slightly translucent. Pelvic fins light gray and slightly translucent. Lateral line dark gray (Figure 12).

**Color in preserved specimens**

Body dark brown/reddish. Ventral side of head and belly yellow or pale yellow. Caudal peduncle slightly darker. Dorsal and anal fins brown at base and translucent at distal end. Caudal fin light brown or gray and translucent. Pectoral fins light brown or gray and translucent. Pelvic fin light brown or gray and translucent. Lateral line dark brown (Figure 11).

**Distribution and habitat**

**FIGURE 13:**
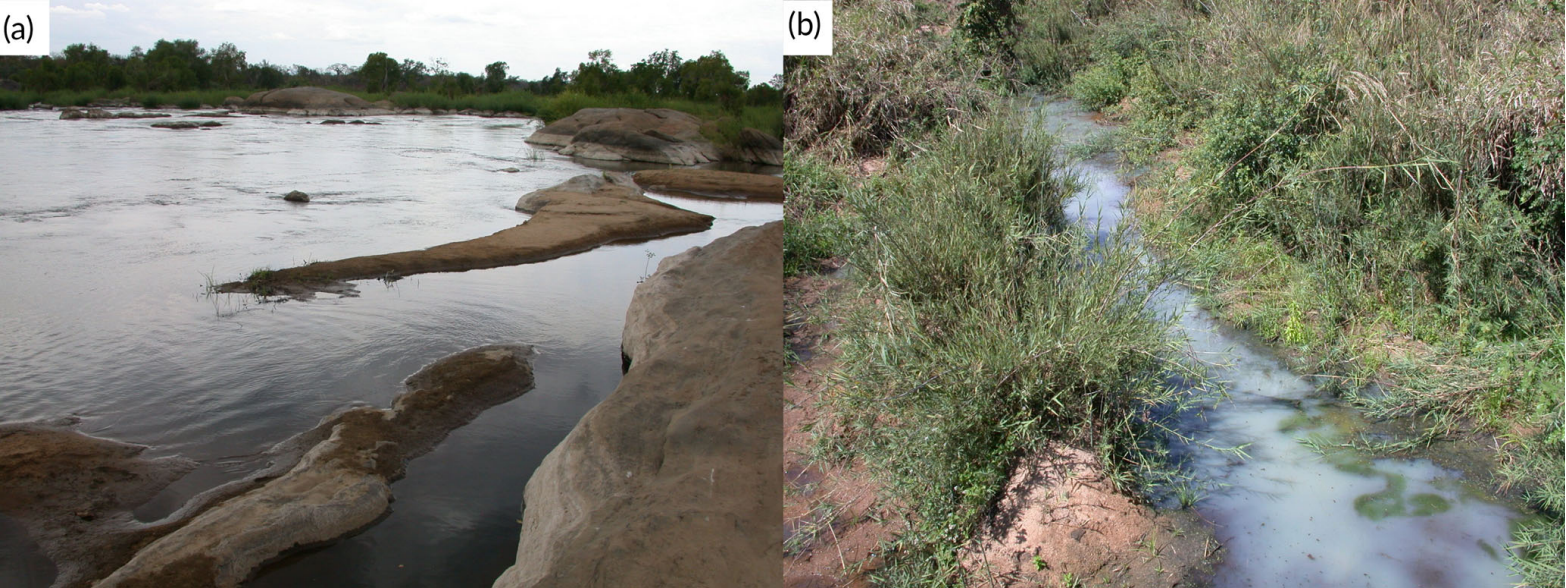
Photographs showing the habitat during the dry season where the specimens of *Pollimyrus krameri* sp. nov. were caught. (a) Main river, the Lugenda. (b) The Nkupo Stream (by R.B., August 22, 2003).

The specimens of *P. krameri* were found in a braid channel, the Nkupo Stream, during the dry season, which is part of the Lugenda River, itself a right bank affluent of the Rovuma River (Mozambique) (Figures 2 and 13). The stream has dense marginal vegetation. The bottom of the river is sandy and rocky, without aquatic weed beds. Marginal *Phragmites* reeds, grasses, and trees result in significant marginal root stocks along some river edges. The small Nkupo Stream was not flowing and did not have aquatic macrophytes, but it was filled with filamentous algae and emergent plant root stocks. During the wet season, this stream would be part of the main river. The specimens were collected using seine netting into and underneath root stocks.

**Etymology**

The specific epithet is a noun honoring Professor Dr. Bernd Kramer (1943–) (University of Regensburg, Germany) for his contributions to ichthyology and study of weakly electric fish, southern African Mormyridae in particular.

**Pollimyrus vanneeri sp. nov**.

Zoobank registration: urn:lsid:zoobank.org:act:5925507B‐C01A‐4319‐8221‐146366363783

**FIGURE 14:**
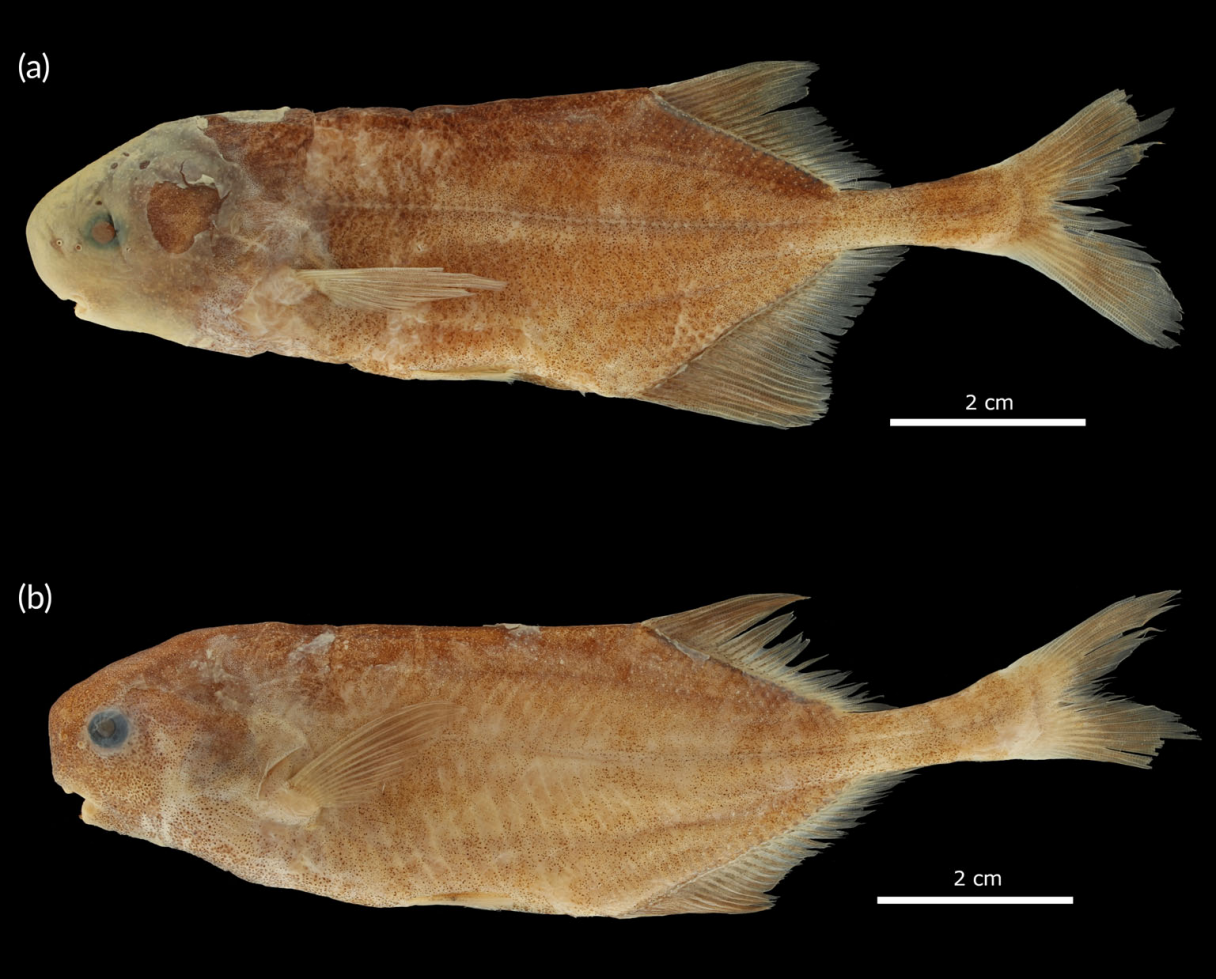
Photographs of preserved specimens of *Pollimyrus vanneeri* sp. nov. (a) Holotype (Royal Museum for Central Africa [RMCA] 1990.057.P.2878: 100.9 mm SL [standard length]); (b) Paratype (RMCA 1990.057.P.2890‐2892: 99.5 mm SL).

Figure 14; Tables 6 and 7

*Pollimyrus* sp. “*kouilou‐niari*”

**Type material**

**Holotype**

**REPUBLIC OF THE CONGO •** Kouilou‐Niari River near Kakamoeka; 4°8′ S, 12°4′ E; October 10, 1990; G. Teugels, L. De Vos and J. Snoeks leg.; 100.9 mm SL; RMCA 1990.057.P.2878.

**Paratypes**

**REPUBLIC OF THE CONGO •** eight paratypes; same data as holotype; 79.9–118.3 mm SL; RMCA 1990.057.P.2879‐2885 **•** one paratype; same data as holotype; 81.3 mm SL; AMNH 281712**•** one paratype; same data as holotype; 85.6 mm SL; BMNH 2024.9.20.1 **•** one paratype; same data as holotype; 83.9 mm SL; MNHN 2024‐0897 **•** one paratype; same data as holotype; 90.6 mm SL; ZSM 49654 **•** two paratypes; same data as holotype; October 12, 1990; 65.6–123.6 mm SL; RMCA 1990.057.P.2893‐2894 **•** three paratypes; confluence of Kissafou and Kouilou‐Niari rivers; 4°7′ S, 12°5′ E; October 11, 1990; G. Teugels, L. De Vos and J. Snoeks; 77.9–99.5 mm SL; RMCA 1990.057.P.2890‐2892.

**Diagnosis**

Distinguished from its congeners by the following unique combination of characters: thick caudal peduncle (20.3%–24.4% BD vs. slender, 12.7%–20.2% BD, in *P. adspersus*, *P. maculipinnis*, *P. osborni*, *P. pedunculatus*, *P. pulverulentus*, *P. schreyeni*, *P. tumifrons*, *P. ibalazambai*, and *C. plagiostoma*); rounded pectoral fins (vs. pointed in *P. adspersus*, *P. fasciaticeps, P. isidori, P. maculipinnis*, *P. nigricans, P. osborni*, *P. pedunculatus*, *P. pulverulentus*, *P. schreyeni*, *P. ibalazambai*, and *C. plagiostoma*); shallow body (22.6%–28.7% SL vs. deep, 29.1%–34.6% SL, in *P. adspersus, P. brevis, P. castelnaui, P. guttatus., P. isidori, P. maculipinnis, P. osborni, P. schreyeni*, and *C. plagiostoma*); short pre‐anal distance (52.4%–58.8% SL vs. long, 59.2%–65.8% SL, in *P. brevis, P. castelnaui, P. guttatus, P. isidori, P. krameri*, *P. nigricans, P. stappersii*, and *P. weyli*); more vertebrae (41–43 vs. fewer, 37–40, in *P. adspersus, P. brevis, P. castelnaui, P. fasciaticeps, P. guttatus, P. isidori, P. maculipinnis, P. marianne, P. nigricans, P. osborni, P. krameri, P. pedunculatus, P. pulverulentus*, *P. stappersii*, and *P. weyli*, and more, 44, in *C. plagiostoma*); 21–24 dorsal‐fin rays (vs. fewer, 14–20, in *P. adspersus, P. brevis, P. castelnaui, P. fasciaticeps, P. guttatus, P. isidori, P. krameri*, *P. maculipinnis, P. marianne, P. osborni, P. pedunculatus, P. schreyeni, P. stappersii*, and *P. weyli*, and more, 32–33, in *C. plagiostoma*); long snout (15.3%–21.0% HL vs. shorter, 10.5%–14.9% HL, in *P. adspersus, P. castelnaui, P. guttatus, P. fasciaticeps, P. krameri*, *P. maculipinnis, P. nigricans, P. osborni, P. pedunculatus, P. petricolus, P. stappersii*, and *P. weyli*); small eye (13.7%–21.6% HL vs. larger, 22.9%–28.7% HL, in *P. adspersus, P. fasciaticeps, P. maculipinnis, P. osborni*, and *P. ibalazambai*); long dorsal fin (22.1%–25.1% SL vs. shorter, 15.2%–20.2% SL, in *P. brevis, P. castelnaui, P. krameri*, *P. maculipinnis, P. marianne, P. nigricans, P. schreyeni, P. stappersii*, and *P. weyli*, and longer, 29.3%–37.9% SL, in *P. tumifrons and C. plagiostoma*). Also see Supplementary file S1 for details and a differential diagnosis with each species.

**Description**

Based on holotype and paratypes. Measurements and meristics are given in Tables 6 and 7. A relatively small species, with a maximum observed size of 123.6 mm SL. Body oblong, somewhat elongated, and dorso‐ventrally compressed. Deepest point of body around anal‐fin origin, and not changing drastically over entire body length. Head blocky and about as deep as body. Mouth small and subterminal, reaching level of eye. No obvious chin. Snout protruding. Anterior nostril positioned higher than posterior one. Posterior nostril close to eye. Nostrils separated by less than half snout–eye distance. Large close‐set, bicuspid teeth in single row, six to eight in upper jaw and five to nine in lower jaw. Arch of teeth filling entire upper and lower jaw. Three most anteriorly teeth positioned at about same level in upper jaw. Anal‐fin origin slightly in front of dorsal‐fin origin. Anal fin longer than dorsal fin. Anterior parts of anal and dorsal fin higher than posterior part with concave diminution. Pectoral fin long, reaching tip of pelvic fin. First three pectoral‐fin rays longer than others with concave diminution. Caudal‐fin lobes somewhat rounded and long. Long and deep caudal peduncle.

**Color in life**

Unknown.

**Color in preserved specimens**

Body generally light brownish or red‐brownish. Head slightly darker. Dorsal side of body darker brown in some specimens. Dorsal and anal fins brown at base and translucent at distal end. Caudal‐fin base white, yellow, or light brown, and distal end translucent. Pectoral and pelvic fins yellow/white and translucent at distal end. Lateral line light brown. Second, darker brown supralateral curved line visible across body (Figure 14).

**Distribution and habitat**

The specimens of *P. vanneeri* were collected near Kakamoeka in the Kouilou‐Niari River and at the confluence of the Kouilou‐Niari River with the Kissafou River, the latter a right bank affluent of the former, Republic of the Congo (Figure 2). The following physicochemical parameters were collected at the type locality: water temperature about 27.7°C in the afternoon; pH 7.8; oxygen level 6.8 mg/L; ammonium concentration 0.1 mg/L; nitrites concentration 0.05 mg/L; silicium concentration 1 mg/L; calcium concentration 18–26 ppm; and phosphate level 0.25 mg/L.

**Etymology**

The specific epithet is a noun honoring Professor Dr. Wim Van Neer (1954–) (Royal Belgian Institute of Natural Sciences, Brussels, and KU Leuven, Leuven, Belgium) for his contributions to ichthyoarchaeology in Europe and northern Africa.

**Pollimyrus weyli sp. nov**.

Zoobank registration: urn:lsid:zoobank.org:act:FB535D7B‐2446‐4DB6‐850A‐E688C2E3F65D

**FIGURE 15:**
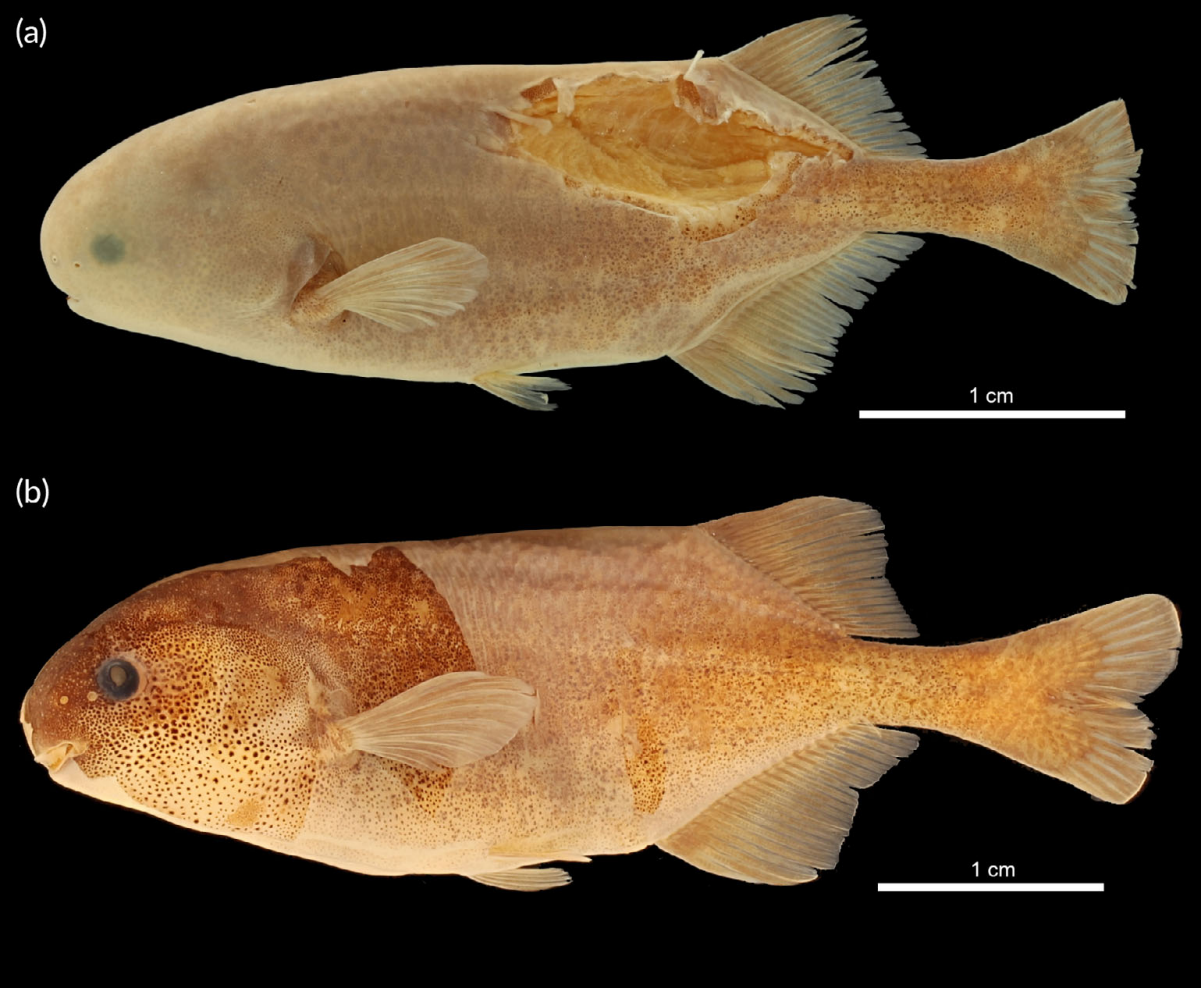
Photographs of preserved specimens of *Pollimyrus weyli* sp. nov. (a) Holotype (South African Institute for Aquatic Biodiversity [SAIAB] 67639: 51.43 mm SL [standard length]). (b) Paratype (SAIAB 67706: 49.1 mm SL).

**FIGURE 16:**
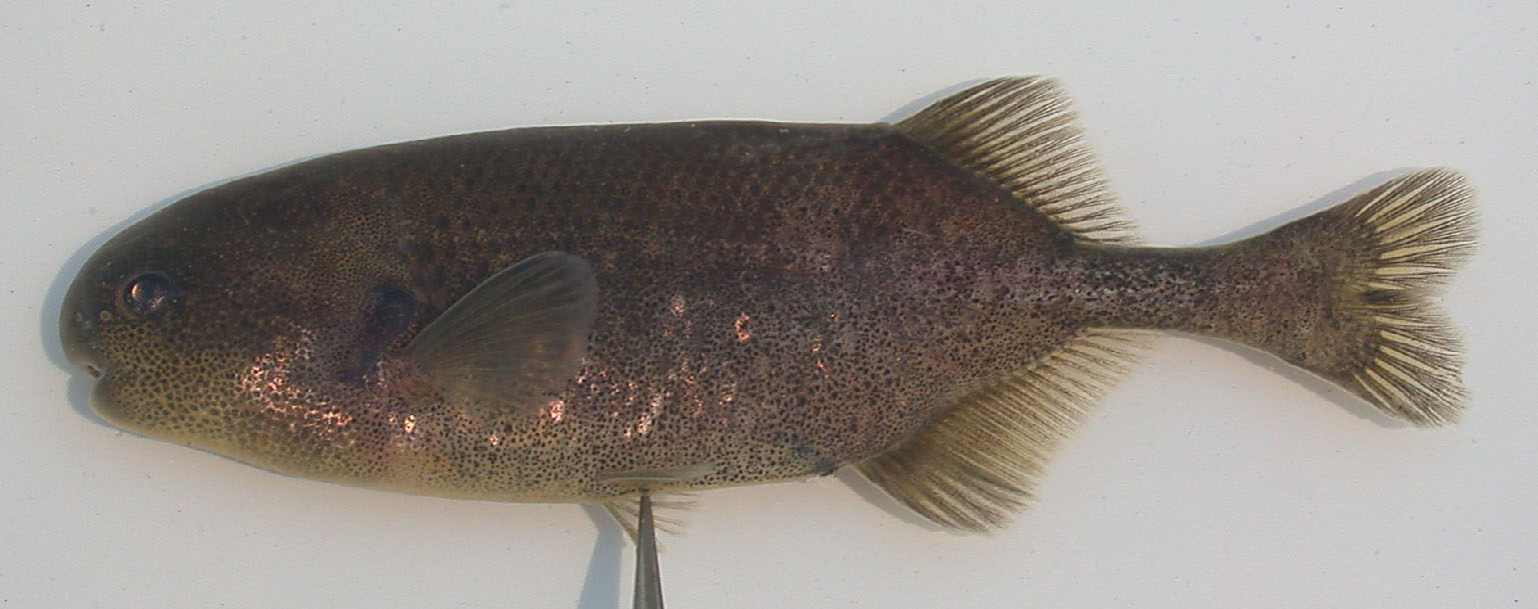
Photograph of dead, but not yet fixed, holotype of *Pollimyrus weyli* sp. nov. (South African Institute for Aquatic Biodiversity [SAIAB] 67639: 51.43 mm SL [standard length]) from the Mussapa River (by R.B., September 27, 2002).

Figures 15 and 16; Tables 6 and 7

*Pollimyrus* sp. “*buzi*”

**Type material**

**Holotype**

**MOZAMBIQUE •** Mussapa River, Krongwa Stream near Dombe; 19°54′44″ S, 33°20′22″ E; September 27, 2002; R. Bills leg.; 51.4 mm SL; SAIAB 67639.

**Paratypes**

**MOZAMBIQUE •** one paratype; Lucite River, Mukombe Stream near Dombe; 19°56′38″ S, 33°29′06″ E; September 29, 2002; R. Bills leg.; 49.1 mm SL; SAIAB 67706.

**Diagnosis**

Distinguished from its congeners by the following unique combination of characters: wide upper jaw (22.6%–24.5% HL vs. slender, 12.7%–21.5% HL, in *P. adspersus, P. castelnaui, P. fasciaticeps, P. guttatus, P. isidori, P. krameri*, *P. maculipinnis, P. marianne, P. nigricans, P. osborni, P. pedunculatus, P. petricolus, P. pulverulentus, P. schreyeni, P. stappersii, P. tumifrons, P. vanneeri*, *P. ibalazambai*, and *C. plagiostoma*); thick caudal peduncle (23.4%–23.8% BD vs. slender, 12.7%–21.9% BD, in *P. adspersus*, *P. maculipinnis*, *P. osborni*, *P. pedunculatus*, *P. pulverulentus*, *P. schreyeni*, *P. stappersii, P. tumifrons*, *P. ibalazambai*, and *C. plagiostoma*, and thicker, 25.4%–28.8% BD, in *P. petricolus*); rounded pectoral fins (vs. pointed in *P. adspersus, P. fasciaticeps, P. isidori, P. maculipinnis, P. nigricans, P. osborni, P. pedunculatus, P. pulverulentus, P. schreyeni, P. ibalazambai*, and *C. plagiostoma*); rounded dorsal and anal fins (vs. pointed in *P. adspersus, P. maculipinnis, P. osborni, P. pedunculatus, P. pulverulentus, P. schreyeni, P. tumifrons, P. ibalazambai*, and *C. plagiostoma*); small chin (vs. no chin in *P. adspersus, P. fasciaticeps, P. isidori, P. maculipinnis, P. nigricans, P. osborni, P. pedunculatus, P. pulverulentus, P. schreyeni, P. vanneeri, P. ibalazambai*, and *C. plagiostoma*); deep body (29.6%–29.9% SL vs. shallow, 22.2%–28.7% SL, in *P. fasciaticeps, P. krameri, P. pedunculatus, P. petricolus*, *P. vanneeri*, and *P. stappersii*, and deeper, 31.9%–34.6% SL, in *P. isidori, P. osborni*, and *C. plagiostoma*); wide head (55.2%–58.7% HL vs. slender, 43.4%–53.8% HL, in *P. adspersus, P. guttatus, P. isidori, P. krameri, P. pedunculatus, P. petricolus*, and *P. tumifrons*); short anal fin (20.2%–22.5% SL vs. longer, 24.0%–35.4% SL, in *P. adspersus, P. brevis, P. castelnaui, P. fasciaticeps, P. guttatus, P. isidori, P. maculipinnus, P. osborni, P. pedunculatus, P. pulverulentus, P. schreyeni, P. tumifrons, P. vanneeri*, *P. ibalazambai*, and *C. plagiostoma*); short pectoral fin (18.1%–19.6% SL vs. longer, 21.1%–27.5% SL, in *P. adspersus, P. fasciaticeps, P. guttatus, P. nigricans, P. osborni, P. pulverulentus, P. schreyeni, P. tumifrons, and P. ibalazambai*); and 17 dorsal‐fin rays (vs. fewer, 15 in *P. krameri*, and more, 19–33, in *P. adspersus, P. isidori, P. pedunculatus, P. petricolus, P. tumifrons, P. vanneeri*, and *C. plagiostoma*). Also see Supplementary file S1 for details and a differential diagnosis with each species.

**Description**

Based on holotype and paratype. Measurements and meristics are given in Tables 6 and 7. A relatively small species, with a maximum observed size of 51.4 mm SL. Body oblong, somewhat elongated, and dorso‐ventrally compressed. Deepest point of body at pelvic‐fin origin, and not changing drastically over entire body length. Head round and as deep as body. Mouth small and subterminal, not reaching level of eye. Slight chin. Snout protruding. Anterior nostril positioned higher than posterior one. Posterior nostril close to eye. Nostrils separated by less than half snout–eye distance. Bicuspid teeth in single row, nine on upper jaw and seven on lower jaw. Arch of teeth filling entire upper and lower jaw. Most anterior tooth on upper jaw clearly positioned anteriorly of second most teeth left and right of it. Anal‐fin origin posterior of dorsal‐fin origin. Anal fin longer than dorsal fin. Anterior parts of anal and dorsal fin slightly higher than posterior part. Anal‐ and dorsal‐fin edges straight. Pectoral fin rounded, not reaching tip of pelvic fin. Caudal‐fin lobes rounded and broad, overlapping in middle. Deep caudal peduncle.

**Color in life**

Body dark brown to reddish. Ventral side of head and belly olive. Dorsal, anal, and caudal fins dark brown or olive at base and slightly translucent at distal end. Pectoral and pelvic fins light gray to light olive, mostly translucent. Lateral line slightly yellowish/red (Figure 16).

**Color in preserved specimens**

Body generally light brownish or yellowish. Small brown spots across body. Dorsal and anal fins brownish or yellowish at base and grayish and slightly translucent at distal end. Caudal fin brownish and slightly translucent. Pectoral and pelvic fins light yellow or light brown, slightly translucent. Lateral line slightly visible as slightly darker brown (Figure 15).

**Distribution and habitat**

**FIGURE 17:**
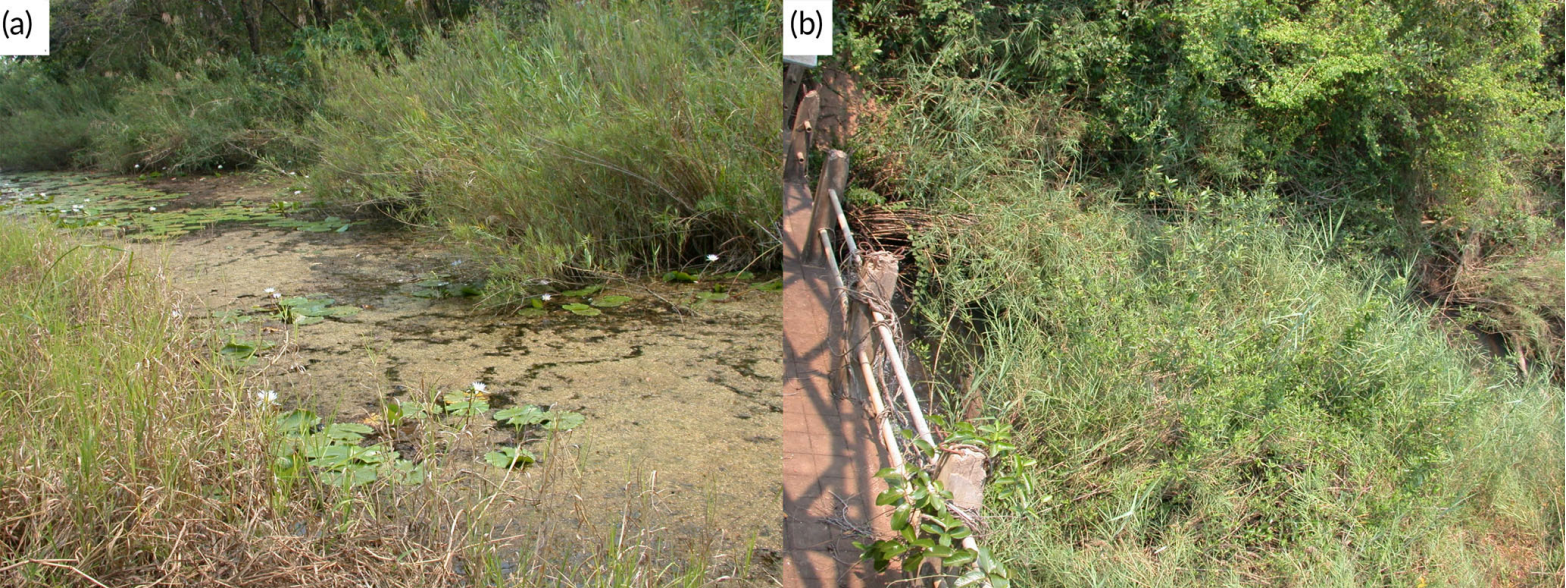
Photographs showing the habitat where the holotype of *Pollimyrus weyli* sp. nov. was caught. (a) Upstream, where the specimens were caught. (b) Downstream (by R.B., September 27, 2002).

The specimens of *P. weyli* were collected in the Mukombe and the Krongwa streams near Dombe of the Mussapa, an affluent to the Lucite River, and Lucite River respectively, a left bank affluent of the Buzi River, Mozambique (Figures 2 and 17). The holotype was caught in a weed‐filled non‐flowing stream close to the main channel of the Buzi River, some 154 m a.s.l. The vegetation consisted mostly of a fine leafed *Potamogeton* (like *Potamogeton pusillus*) and a few water lilies (*Nymphaea*). The vegetation cover was close to 100%, where the specimen was caught by raking a big hard‐framed hand‐net through the weeds. The water depth was less than 1 m with a mixed substrate of sand and rocks. The paratype was caught in a lower‐altitude tributary, at 138 m a.s.l., with a good flow, completely covered over by emergent *Phragmites*. There was no submerged aquatic vegetation. Collected by electrofishing with a *Samus* backpack. Water depth was less than 1 m with a substrate of cobbles and banks undercut with a lot of rootstocks.

**Etymology**

The specific epithet is a noun honoring the late Professor Dr. Olaf L.F. Weyl (1972–†2020) (SAIAB) for his contributions to African ichthyology and his work in the Buzi River system within the framework of his PhD, expanding the collections housed at SAIAB and increasing the understanding of the biodiversity in the region.

### 3.4 Identification key to species, based on the type specimens of the genus *Pollimyrus* Taverne, 1971

A dichotomous identification key using the morphological features (see methods for definitions) of the type specimens of the *Pollimyrus* species (and *C. plagiostoma* for completeness: see Results) is provided below. As this key is based only on type specimens, the morphological variation possible within a species is not reflected here due to the low number of specimens available for some species. Morphological features, especially measurements, should be used cautiously if the differences between species are minimal. Photos of all species can be found in the Supplementary file S1 (Figures S1–S19).

1. Anal fin twice as long as dorsal fin (LA 34.7%–35.4% SL vs. LD 15.2%–16.2% SL); 30–32 anal‐fin rays ***P. schreyeni* Poll, 1972**
  ‐ Anal fin not twice as long as dorsal fin (LA 20.2%–30.8% SL vs. LD 16.0–37.9% SL); 29 or fewer anal‐fin rays .…………………… **2**
2. 32–33 dorsal‐fin rays; dorsal fin slightly longer than anal fin (LD 34.4%–37.9% SL vs. LA 26.5%–27.8% SL) ***C. plagiostoma* (Boulenger, 1898)**
  ‐ 14–28 dorsal‐fin rays; dorsal and anal fin of similar length (LD 16.0%–30.6% SL vs. LA 20.2%–30.8% SL)…………………………..**3**
3. 12 pectoral‐fin rays; anterior nostril positioned lower than the posterior nostril; inferior mouth; posterior part of the mouth reaching level of the eye ***P. tumifrons* (Boulenger, 1902)**
  ‐ No such combination of characteristics ………………………………… **4**
4. Anterior nostril positioned lower than the posterior nostril; very long (CPL 22.8% SL) and slender (CPD 20.6% CPL) caudal peduncle ***P. pedunculatus* (David & Poll, 1937)**
  ‐ Anterior nostril positioned higher or same level as posterior nostril; caudal peduncle not as long (CPL 13.6%–20.8% SL) and slender (CPD 25.0%–52.5% CPL) ………………………………**5**
5. Caudal peduncle depth about five times or more in body depth at pelvic‐fin origin (CPD 12.7%–20.2% BD)**6**
  ‐ Caudal peduncle depth less than five times in body depth at pelvic‐fin origin (CPD 21.5%–31.2% BD) ………………………………**10**
6. Caudal peduncle depth about five times in body depth at pelvic‐fin origin (CPD 19.4%–20.2% BD); 7 teeth in upper jaw; 17 dorsal‐fin rays; 24 anal‐fin rays ***P. maculipinnis* (Nichols & LaMonte, 1934)**
  ‐ No such combination of characteristics ………………………………**7**
7. Long pre‐pectoral distance (PPL 26.6%–30.2% SL); tooth cusps not protruding and small ***P. pulverulentus* (Boulenger, 1899) (including *P. nigripinnis* [Boulenger, 1899])**
  ‐ Short pre‐pectoral distance (PPL 22.5%–25.9% SL); protruding tooth cusps **8**
8. Teeth small and with clear spaces between them **P. ibalazambai sp. nov**.
  ‐ Teeth large and filling the jaws, that is, almost without space between them **9**
9. Head wide (HW 57.5% HL); long pre‐pelvic distance (PVL 42.2% SL); 17 dorsal‐ and 24 anal‐fin rays ***P. osborni* (Nichols & Griscom, 1917)**
  ‐ Head slender (HW 51.1%–51.9% HL); short pre‐pelvic distance (PVL 36.0%–36.7% SL); 19–20 dorsal‐ and 27 anal‐fin rays ***P. adspersus* (Günther, 1866)**
10. Rounded snout; small chin (i.e., enlargement at frontal side of lower jaw) **11**
  ‐ Blocky snout; no chin ………………………………**18**
11. 58–67 lateral‐line scales; 19–22 dorsal‐fin rays; 41–43 vertebrae; long dorsal fin (LD 89.2%–112.3% BD) ***P. petricolus* (Daget, 1954)**
  ‐ 40–54 lateral‐line scales; 15–19 dorsal‐fin rays; 36–41 vertebrae; short dorsal fin (LD 58.6%–81.9% BD) ………………………… **12**
12. Slender caudal peduncle (CPD 5.9%–6.1% SL); long belly (BL 20.8%–21.6% SL) ***P. stappersii* (Boulenger, 1915)**
  ‐ Thick caudal peduncle (CPD 6.6%–8.8% SL); short belly (BL 17.0%–20.9% SL) ………………………………**13**
13. Broad lower jaw (LJW 23.5%–25.7% HL); 18 dorsal‐fin rays; 23–25 anal‐fin rays; 39 vertebrae ***P. brevis* (Boulenger, 1913)**
  ‐ Slender lower jaw (LJW 13.6%–22.1% HL); no such combination of characteristics ………………………………**14**
14. Broad upper jaw (UJW 22.6%–24.4% HL) **P. weyli sp. nov**.
  ‐ Slender upper jaw (UJW 13.3%–20.2% HL) ………………………… **15**
15. Large eye diameter (OD 22.1%–27.3% HL); long pectoral fins (LP 23.2%–26.1% SL); deep body (BD 30.7%–32.1% SL); 18–19 dorsal‐fin rays ***P. guttatus* (Fowler, 1936)**
  ‐ No such combination of characteristics ……………………………… **16**
16. Deep body (BD 29.5%–31.2% SL); wide lower jaw (LJW 21.7%–22.1% HL); pelvic fin less than half the length of belly (LV 9.7% SL vs. BL 19.6%–20.7% SL) ***P. castelnaui* (Boulenger, 1911)**
  ‐ Shallow body (BD 25.2%–29.8% SL); slender lower jaw (LJW 13.6%–21.1% HL); pelvic fin slightly longer than half the belly length (LV 9.5%–11.9% SL vs. BL 17.0%–20.1% SL) ………………**17**
17. Short post‐dorsal distance (pD 36.8%–37.0% SL); slender head (HW 52.7%–53.8% HL); small eye (OD 14.3%–15.5% HL); short snout length (SPE 39.4%–39.7% HL) **P. krameri sp. nov**.
  ‐ No such combination of characteristics ………………… ***P. marianne* Kramer et al., 2013 (including *P. cuandoensis* Kramer et al., 2013)**
18. Rounded pectoral fins; 21–24 dorsal‐fin rays; 59–73 lateral‐line scales; 41–43 vertebrae **P. vanneeri sp. nov**.
  ‐ Pointed pectoral fins; 14–20 dorsal‐fin rays; 48–55 lateral‐line scales; 37–39 vertebrae ………………………………**19**
19. Short dorsal fin (LD 16.0%–19.1% SL); short post‐dorsal distance (pD 35.5%–37.5% SL); 12 scales between pelvic‐fin origin and lateral line ***P. nigricans* (Boulenger, 1906)**
  ‐ Long dorsal fin (LD 22.3%–22.5% SL); long post‐dorsal distance (pD 42.8%–43.5% SL); 14–16 scales between pelvic fin origin and lateral line ………………………………**20**
20. 20 dorsal‐fin rays; deep body (BD 33.3% SL); slender head (HW 50.9% HL) ***P. isidori* (Valenciennes, 1847)**
  ‐ 17 dorsal‐fin rays; shallow body (BD 29.7% SL); wide head (HW 59.7% HL)
***P. fasciaticeps* (Boulenger, 1920)**

### 3.5 Comparative material

All studied specimens of previously described species are listed below, ordered alphabetically, first, by their genus and second, by species name. The nomenclature and taxonomy proposed in this study are followed. Proposed junior synonyms are listed under their senior synonyms.

*Cyphomyrus petherici* (Boulenger, 1898)

**SUDAN •** three syntypes; Upper Nile River, Khartoum; ~ 15°36′ N, 32°32′ E; Petherick leg.; 127.5/175.3/194.2 mm SL; BMNH 1862.6.17.92/BMNH 1862.6.17.22‐23.

*Cyphomyrus plagiostoma* (Boulenger, 1898)

**DEMOCRATIC REPUBLIC OF THE CONGO •** two syntypes; Matadi; ~ 5°49′ S, 13°27′ E; Wilverth leg.; 88.4–100.2 mm SL; BMNH 1898.11.12.13‐14.

*Paramormyrops eburneensis* (Bigorne, 1991)

**IVORY COAST •** one holotype; Agnébi, Agnibilékrou; ~ 7°08′ N, 3°12′ E; April 1964; de Rham leg.; 72.9 mm SL; MNHN IC‐1990.0376 **•** one paratype; same data as holotype; 75.6 mm SL; MNHN IC‐1970.0006 **•** one paratype; same data as holotype; May 1970; 48.1 mm SL; MNHN IC‐1970.0086 **•** one paratype; San Pedro; ~ 4°44′ N, 6°37′ E; February 1977; Lévêque and Paugy leg.; 53.2 mm SL; MNHN IC‐1979.0135 **•** four paratypes; Banco, close to Abidjan, stream flowing from the Ebié Lagune; ~ 5°22′ N, 4°03′ W; 1966; Thys van den Audenaerde leg.; 59.1–75.9 mm SL; RMCA 1973.005.P.0049‐0053.

*Pollimyrus adspersus* (Günther, 1866)

**WEST AFRICA •** two syntypes; Steven and Damon leg.; 57.9–58.9 mm SL; BMNH 1865.5.3.41.

*Pollimyrus brevis* (Boulenger, 1913)

**DEMOCRATIC REPUBLIC OF THE CONGO •** one syntype; Upper Uele River near Dungu; 3°37′ N, 28°33′ E; Hutereau leg.; 54.0 mm SL; RMCA P.1804 **•** one syntype; same data as previous; 50.2 mm SL; BMNH 1912.12.6.1.

*Pollimyrus castelnaui* (Boulenger, 1911)

**BOTSWANA •** two syntypes; Lake Ngami; ~ 20°30′ S, 22°40′ E; Woosman leg.; 58.3–62.5 mm SL; BMNH 1910.5.31.11‐12.

*Pollimyrus fasciaticeps* (Boulenger, 1920)

**DEMOCRATIC REPUBLIC OF THE CONGO •** one holotype; Léopoldville (now Kinshasa); 4°18′ S, 15°18′ E; 1912; Christy leg.;49.3 mm SL; RMCA P.7243.

*Pollimyrus guttatus* (Fowler, 1936)

**CAMEROON •** one holotype; 30 km east of Kribi; 2°57′ N, 9°55′ E; November 26, 1934; Vanderbilt and von Blixen leg.; 39.5 mm SL; ANSP 65504 **•** one paratype; same data as holotype; 36.3 mm SL; ANSP 65505 **•** two paratypes; same data as holotype; November 24–25, 1934; 37.0–39.2 mm SL; ANSP 65506.

*Pollimyrus isidori* (Valenciennes, 1847)

**EGYPT •** one holotype; Nile River; 1799; Geoffroy Saint‐Hilaire leg.; 84.3 mm SL; MNHN IC‐4209.

*Pollimyrus maculipinnis* (Nichols and LaMonte, 1934)

**DEMOCRATIC REPUBLIC OF THE CONGO •** one holotype; Lulua River, near Luluabourg (now Kananga); ~ 5°54′ S, 22°25′ E; August 7, 1932; Callewaert leg.; 52.9 mm SL; AMNH I‐12355 **•** one paratype; same data as holotype; August 2, 1932; 53.9 mm SL; AMNH I‐12400.

*Pollimyrus marianne* Kramer et al., 2003.

**NAMIBIA/ZAMBIA •** 1 holotype; Zambezi River, Lisikili backwater; 17°29′ S, 24°26′ E; April 7, 1996; Kramer and van der Bank leg.; 63.9 mm SL; SAIAB 66943 **•** 10 paratypes; same data as holotype; 57.9–66.6 mm SL; SAIAB 66944 **•** 1 specimen.

*Pollimyrus cuandoensis* Kramer et al., 2013 (now synonym of *P. marianne*)

**NAMIBIA •** 1 holotype; Kwando River at Kongola Bridge; 17°47′33″ S, 23°20′33″ E; August 25, 1999; Kramer, van der Bank and Wink leg.; 44.1 mm SL; ZSM 41805 **•** 11 paratypes; same data as holotype; 38.1–52.7 mm SL; ZSM 39522.

*Pollimyrus nigricans* (Boulenger, 1906)

**UGANDA •** nine syntypes; mouth of Katonga River near Lake Victoria; ~ 0°02′ S, 32°01′ E; November 5, 1905; Degen leg.; 76.9–86.8 mm SL; BMNH 1906.5.30.85‐94.

*Pollimyrus osborni* (Nichols and Griscom, 1917)

**DEMOCRATIC REPUBLIC OF THE CONGO •** one holotype; Uele River; ~ 4°09′ N, 22°26′ E; 1909; Lang and Chapin leg.; 53.0 mm SL; AMNH I‐6934.

*Pollimyrus pedunculatus* (David & Poll, 1937)

**DEMOCRATIC REPUBLIC OF THE CONGO •** one holotype; Congo River, Boma; 5°50′ S, 13°3′ E; Van Delft leg.; 67.7 mm SL; RMCA P.22664.

*Pollimyrus petricolus* (Daget, 1954)

**MALI •** one holotype; Upper Niger, Markala; 13°41′ 15″ N, 6°05′ 13″ W; 1954?; 57.9 mm SL; MNHN IC‐1954.0008 **•** two paratypes; same data as holotype; April 24, 1951; Daget leg.; 50.3–85.1 mm SL; MNHN IC‐1960.0406 **•** one paratype; same data as holotype; May 25, 1952; Daget leg.; 52.7 mm SL; MNHN IC‐1960.0407.

*Pollimyrus pulverulentus* (Boulenger, 1899)

**DEMOCRATIC REPUBLIC OF THE CONGO •** one syntype; Coquilhatville (now Mbandaka); 0°4′ N, 18°16′ E; Delhez leg.; 89.9 mm SL; RMCA P.612 **•** one syntype; same data as previous; 88.8 mm SL; RMCA P.613 **•** two syntypes; same data as previous; 61.6–80.8 mm SL; BMNH 1899.9.26.26‐27.

*Pollimyrus nigripinnis* (Boulenger, 1899)

**DEMOCRATIC REPUBLIC OF THE CONGO •** one syntype; Lake Mai‐Ndombe/Lake Leopold II (now Lake Mai Ndombe), Kutu; 2°44′ S, 18°8′ E; Delhez leg.; 78.8 mm SL; RMCA P.608 **•** one syntype; same data as previous; 88.5 mm SL; RMCA P.609 **•** one syntype; same data as previous; 102.2 mm SL; RMCA P.610 **•** one syntype; same data as previous; 86.9 mm SL; RMCA P.611 **•** two syntypes; same data as previous; 72.5–95.2 mm SL; BMNH 1899.9.26.28–29 **•** one syntype; Uéré River; 4°2′ N, 25°51′ E; Debauw leg.; 61.3 mm SL; RMCA P.344.

**REPUBLIC OF THE CONGO •** five specimens; Lefini River basin, Lac Bleu; 3°19.07′ S, 15°28.74′ E; August 16, 2007; Ibala Zamba leg.; 63.4–114.3 mm SL; RMCA 2007.031.P.0388–0392.

*Pollimyrus schreyeni* Poll, 1972

**DEMOCRATIC REPUBLIC OF THE CONGO •** one holotype; river 15 km from Boende, along route Boende‐Watsi; 0°14′ S, 20°57′ E; May 1970; Brichard leg.; 70.5 mm SL; RMCA P.174708 **•** two paratypes; same data as holotype; 71.3–75.8 mm SL; RMCA P.174709–19 **•** one paratype; Bokuma; 0°6′ S, 18°41′ E; June 1953; Lootens leg.; 73.15 mm SL; RMCA P.88293.

*Pollimyrus stappersii* (Boulenger, 1915)

**DEMOCRATIC REPUBLIC OF THE CONGO •** one holotype; Lukinda River basin; 8°31′ S, 28°58′ E; February 26, 1912; Stappers leg.; 59.3 mm SL; RMCA P.12689 **•** one holotype of *P. kapangae* (David, 1935); Katanga, Kapanga; 11°30′ S, 26°45′ E; 1933; Overlaet leg.; 61.6 mm SL; RMCA P.39492.

*Pollimyrus tumifrons* (Boulenger, 1902)

**DEMOCRATIC REPUBLIC OF THE CONGO •** one holotype; Ubangi River, Banzyville (now Mobayi‐Mbongo); 4°18′ N, 21°10′ E; 1901; Royaux leg.; 88.6 mm SL; RMCA P.1160 **•** one holotype of *P. aequipinnis* (Pellegrin, 1924); Kasaï River, N'Gombe; 6°35′ S, 20°43′ E; Schoutenden leg.; 90.1 mm SL; RMCA P.15188 **•** one holotype of *P. anterodorsalis* (David & Poll, 1937); Aruwimi River, Panga; 1°52′ N, 26°23′ E; May 26, 1926; Bock leg.; 44.8 mm SL; RMCA P.22167.

## 4 DISCUSSION

### 4.1 An updated diagnosis of the genus *Pollimyrus*

This study provides a long‐overdue morphometric alpha‐taxonomic summary of the genus *Pollimyrus*. To date, this genus contained 18 valid species (see Table 1), which has now been revised to 20. Since its original description by Taverne (1971a, 1971b), this genus has been dealing with quite numerous reallocations of species to different genera (Table 1). Nevertheless, this seems to have resulted in some apparently incorrect or undecisive generic attributions. By morphologically analysing most available type specimens of these species (Table 2), their generic attribution status has been clarified or, otherwise, discussed.

A closer look at the type specimens of “*Pollimyrus*” *eburneensis* reveals that these do not belong to *Pollimyrus*, as suggested by Rich et al. (2017) as a taxonomic note with no further explanation, but rather *Paramormyrops* Taverne, Thys van den Audenaerde, and Heymer, 1977. Several features, especially the fusion of ventral hypurals and body depth, do not match the characteristics of *Pollimyrus* spp. This confirms the need to reallocate this species to another genus. Following the identification key to the mormyrid genera, as provided by Sullivan et al. (2016), “*P”. eburneensis* fits in the genus *Paramormyrops*. *Paramormyrops eburneensis* (Bigorne, 1991) was originally described as a subspecies of *Paramormyrops kingsleyae* (Günther, 1896), which at the time was assigned to the genus *Pollimyrus*. *Pollimyrus kingsleyae* has been reassigned to *Brienomyrus* by Teugels and Hopkins (1998) and later to *Paramormyrops* by Hopkins et al. (2007) due to this species having the two ventral hypurals unfused, resulting in a total of five hypurals, whereas in *Pollimyrus* these are fused, resulting in a total number of four hypurals only. In addition, its elongated body shape (body depth 4.7 times in SL vs. 2.7–4.2 times in species of the genus *Pollimyrus* [Teugels & Hopkins, 1998]) confirms its identification as a member of that genus. Regarding the status of *Paramormyrops eburneensis* as a potential subspecies of *Paramormyrops kingsleyae*, no further remarks are made here, as this falls outside the scope of this study.

As already mentioned by Lavoué et al. (2010), “*Petrocephalus*” *guttatus* does not show some of the diagnostic characteristics of that genus, such as the closely positioned nostrils and the position of the mouth at the level of the eyes. It does, however, resemble *P. isidori* and other *Pollimyrus* spp. for those characteristics (Lavoué et al., 2010; see Results). In addition, all meristics fall within the ranges known for *Pollimyrus* species (see Tables 6 and 7). Because there is no further resemblance to *Petrocephalus*, the species is here reassigned to *Pollimyrus*.

*C. plagiostoma* shares diagnostic characteristics not only with *Pollimyrus* but also with *Cyphomyrus*. The number of teeth and the caudal peduncle depth would indicate that this species can indeed be assigned to *Pollimyrus*, whereas the fin implantation and dorsal‐fin length contradict this, making *Cyphomyrus* a better generic assignment. Therefore, Myers (1960) placed this species in *Cyphomyrus* when erecting the genus, after which Taverne (1971a, 1971b) reassigned the species to the genus *Pollimyrus* based on it having only fused instead of unfused ventral hypurals, which he considered diagnostic for the genus *Pollimyrus*. The usefulness of the fusion of the ventral hypurals to distinguish *Pollimyrus* from *Cyphomyrus* is, however, uncertain. The type specimens of *C. psittacus*, the type species of the genus *Cyphomyrus*, have unfused ventral hypurals (Taverne, 1971a, 1971b), whereas the three syntype specimens of *C. petherici* have only fused ventral hypurals (see X‐rays in Supplementary Information S1), explaining its assignment to *Pollimyrus* by Taverne (1971a, 1971b). This, however, shows that this osteological feature is not sufficient to differentiate both genera. Recently *C. plagiostoma* was reassigned to *Cyphomyrus* based on morphological similarities to other *Cyphomyrus* species and unpublished genetic data (Stiassny et al., 2021). A more recent, whole‐genome analysis, however, placed this species within *Pollimyrus* (Peterson et al., 2022). Although the results of Peterson et al. (2022) are not in discussion, due to the absence of published photographs, it was not possible to assess morphological similarities of these genetically analysed specimens (one being CUMV 96188 from Boyoma Falls), with the type specimens from Matadi. Therefore, the present generic assignment of *C. plagiostoma* is here retained as *Cyphomyrus* for reasons of nomenclatorial stability. A thorough morphological, osteological, and genetic comparison of both genera *Pollimyrus* and *Cyphomyrus* is needed to further clarify the issue. At this point, only the dorsal‐fin length and the number of dorsal‐fin rays make it possible to unmistakably differentiate *Cyphomyrus* from *Pollimyrus*. *Cyphomyrus plagiostoma* is therefore easily distinguished from all *Pollimyrus* species (see Results and Tables 6 and 7 for details).

*Pollimyrus tumifrons* was retained within the genus for nomenclatorial stability, although several characteristics seem atypical for the genus (see Results). Following Sullivan et al. (2016)'s identification key, *Pollimyrus* remains the best fit for this species, despite there being some confusion regarding the relative length of dorsal and anal fins and the number of fin rays as diagnostic features between *Pollimyrus* and *Hippopotamyrus* Pappenheim, 1906. Recently, *Hippopotamyrus macroterops* (Boulenger, 1920) was reassigned to *Pollimyrus* by Sullivan et al. (2022) due to morphological similarities with *P. tumifrons* and *C. plagiostoma*. We agree that *P. macroterops* and *P. tumifrons* are morphologically very similar based on photographs of *P. macroterops* (Sullivan & Lavoué, 2024) and have a similar hydro‐geographical distribution in the Ubangi basin. As *P. macroterops* was not assigned to *Pollimyrus* at the start of this study, it was overlooked as a potential *Pollimyrus* species and not included in the comparative material. This recent reassignment and the uncertainty of *P. tumifrons* being correctly assigned to the genus *Pollimyrus* show that the diagnosis between *Pollimyrus* and *Hippopotamyrus* should be re‐examined.

These examples illustrate that the generic placement of some species within the Mormyridae is still in need of further taxonomic attention. Further, due to these generic misplacements, it also illustrates the difficulties one might be confronted with when trying to attribute specimens to an existing or potentially new species for science. Nevertheless, this study has allowed for an update of the morphological diagnosis of the genus *Pollimyrus*, sensu Taverne (1971a, 1971b, 1972), Bigorne (2003), and Hopkins et al. (2007), after the inclusion and exclusion of species since its original description. The species presently attributed to the genus *Pollimyrus* have a small lateral ethmoid bone, a large and curved mesethmoid bone, six circumorbital bones, with the antorbital and first infraorbital not fused, and fused or potentially unfused ventral hypural bones. Further, they have a short or rather elongated body. Their snout is shorter than the postorbital part of the head. The mouth is terminal, inferior, or subinferior. The caudal peduncle depth fits two to five times in the peduncle length. Meristically, they have 14–28 dorsal‐fin rays, 20–32 anal‐fin rays, 9–12 pectoral‐fin rays, 35–73 lateral‐line scales, 7–21/8–23 scales in transverse line, 8–18/6–20 scales in transverse line between the dorsal‐ and anal‐fin origins, 11–23 scales around the caudal peduncle, 5–10/5–11 bicuspid teeth on the oral jaws, and 37–45 vertebrae (Taverne, 1971a, 1971b, 1972; this study). Besides the aforementioned characters, these species can further be recognized by newly added characteristics following later authors: rounded or blocky head; no or very small mental lobe; nostrils that are well separated with posterior one lying close to anterior rim of eye; and dorsal‐ and anal‐fin origins at the same level (Bigorne, 2003; Hopkins et al., 2007; this study).

### 4.2 Intrageneric taxonomic changes

The morphological groups identified based on the caudal peduncle depth and several qualitative characteristics are confirmed by whole‐genome evidence, albeit using non‐type specimens (see Peterson et al., 2022; Figure 2). These authors found that the 12 analysed taxa, including *C. plagiostoma*, clearly form two well‐supported clades, one with thick‐tailed and one with slender‐tailed *Pollimyrus*. If confirmed with further genetic evidence, this distinction between the morphological groups could potentially be formalized.

Several of the studied nominal species were found to show little‐to‐no morphological differentiation and therefore confirmed to be synonyms or, for the first time here, identified as synonyms.

*Pollimyrus pulverulentus* and *P. nigripinnis* cannot be distinguished from each other based on the studied meristic and morphometric characteristics (Table 5). Nevertheless, they can be separated using a PCA analysis of both log‐transformed measurements and meristics when considering only these taxa (Figure 6; Supplementary file S1). The measurements and meristics with the most important loadings found in the PCA, however, show a strong overlap. Both species occur relatively close hydro‐geographically, that is, Mbandaka (formerly Coquilhatville), situated on the Middle Congo River itself, for the former, and Kutu at Lake Mai‐Ndombe, which is part of the Middle Congo River for the latter (Figure 2). The minor morphometric differences between the two species detected using PCA could be linked to their distinct hydro‐geographical occurrences or local environmental influences. Therefore, both these species are here regarded as synonyms. Both species seem to be phylogenetically closely related, as shown using non‐type specimens, although a specimen identified as *P. osborni* falls in the same clade (Peterson et al., 2022; Figure 2). Because both species were described in the same publication (Boulenger, 1899), there is no seniority of either of these names (ICZN, 1999: Art. 23). Both species are reported to have black spots on the body (Boulenger, 1899). As such, the name “pulverulentus,” which could refer to black freckles (Scharpf, 2024), still applies to all type specimens of both the nominal species. *Pollimyrus nigripinnis* is here identified as a junior synonym of *P. pulverulentus*.

Nevertheless, one of the syntypes of *P. nigripinnis* (RMCA P.344), now a junior synonym of *P. pulverulentus*, was collected from the Uéré River, a right bank affluent of the Uele River, itself a tributary of the Ubangi River (Middle Congo basin) in the north of the DRC near the village of Ango, whereas all other syntypes of the species originate from Lake Mai‐Ndombe, a right bank affluent of the Kasai basin (Cuvette Centrale/Middle Congo basin) in the west of the DRC near the town of Kutu. The type specimen from Uéré clearly differs from the other syntypes in its morphology, that is, by having a deeper caudal peduncle, wider and longer head, shorter dorsal fin, wider lower jaw, smaller interorbital distance, subterminal mouth, fewer dorsal‐fin rays and vertebrae, and more upper jaw teeth (Table 5 and Results). Because this is the only type specimen from that locality and given that it is not very well preserved, it remains difficult to assess its conspecificity with the other type specimens of the species. Therefore, one should be cautious to make any interpretations regarding *P. nigripinni*s based on this specimen.

No morphometric differences were found between *P. cuandoensis* and *P. marianne* (Figure 4), both species that occur in the Upper Zambezi basin (Figure 2). They only separate on PC 1 in the PCA, which is a proxy for size (see Methodology). Kramer et al. (2013) described morphological differences in the mean and medians of measurements and meristics between both *P. cuandoensis* and *P. marianne*, such as the number of circumpeduncular scales, lateral‐line scales, and dorsal‐fin rays, caudal peduncle depth, length of anal fin, body depth, eye diameter, snout–posterior side of the eye, and pectoral–pelvic fin distance. However, these differences were not found in the present study. When considering the full ranges of measurements and meristics, these species cannot be morphologically distinguished on any of the studied characteristics (see Tables 6 and 7). In the original species description of *P. cuandoensis*, genetic ISSR profiling did show some differentiation between both species (Kramer et al., 2013; Table 2). However, the suggested diagnostic black electrogel band did not show consistently within *P. cuandoensis*. Further, a molecular phylogram based on cytochrome b did not show a clear clustering of *P. marianne* versus *P. cuandoensis* either (Kramer et al., 2013; Figure 7). Therefore, we do not consider the genetic differences to be sufficient to validate *P. cuandoensis* as a separate species. These two species are most easily distinguished by their EOD patterns (Kramer et al., 2013). We are, however, hesitant to only use EOD signals and their characteristics to define species, due to their largely unknown intra‐/interspecific variation and differentiation, which is dependent on, for example, life‐history traits and the local environments (e.g., Bass & Hopkins, 1985; Carlson et al., 2000; Nguyen et al., 2020; Terleph & Moller, 2003; Westby & Kirschbaum, 1978). The EOD differences reported could thus also be due to size differences, as the *P. cuandoensis* specimens are of smaller size (38.11–52.7 mm SL) compared to the larger‐sized specimens (57.93–66.6 mm SL) identified as *P. marianne*. Therefore, those EOD differences do not necessarily support the identification of distinct species (Nguyen et al., 2020; Westby & Kirschbaum, 1978). The difference in size, and thus likely in age, could also explain the small difference in dentition observed. The teeth on the frontal part of the jaws seem slightly larger than those on the posterior part of the jaw in *P. marianne*; however, this was not true for *P. cuandoensis*. No information on ontogenetic changes in dentition of Mormyridae in literature, however, could be found. Therefore, *P. cuandoensis* is here identified as a junior synonym of *P. marianne*, as there is no clear argument that they are, in fact, separate parapatric species occurring in adjacent rivers of the Upper Zambezi basin.

Possible synonymy of *P. stappersii* and *P. castelnaui* was already noticed by Bell‐Cross (1976). Their close phylogenetic relationship has also recently been confirmed using whole‐genome analysis of non‐type specimens (Peterson et al., 2022; Figure 2). The only morphological difference reported by Bell‐Cross (1976) was the higher number of lateral‐line scales of *P. castelnaui* (46–53) compared with *P. stappersii* (no numbers reported). This difference was, however, not observed in the present study. In fact, the opposite seems to be true (47 lateral‐line scales in *P. castelnaui* and 48–53 in *P. stappersii*). Only one difference in meristics has been found in this study: the number of scales between the lateral line and the anal‐fin origin (Table 7). Further, a single difference in measurements was found during this study that allows for their diagnosis, that is, the width of the lower jaw. As a result, two independent characteristics, one meristic and one measurement, seem to support their distinct species status. Because they occur in different river systems, with *P. castelnaui* originally described from Lake Ngami basin of the Okavango basin in Botswana (Zambezi IP) (Boulenger, 1911), whereas *P. stappersii* was described from the Lukinda River basin in the Upper Congo (Congo IP) (Boulenger, 1915), they are considered valid species here due to their clear geographical isolation.

Although *P. fasciaticeps* has been regarded as a synonym of *P. isidori* or *P. osborni* in the past, in this study, we have found sufficient morphological evidence in the length between snout and eye, head width, and caudal peduncle depth to revalidate *P. fasciaticeps* as a species, distinct from *P. isidori* and *P. osborni*. There remain, however, several uncertainties regarding the identification of non‐type specimens of the complex of *P. fasciaticeps* and *P. osborni*. Both species are regarded as valid species in this study. No morphological differences were found between the examined non‐type specimens previously identified as *P. fasciaticeps* collected from the Lower Congo and the Léfini River in the Middle Congo, the non‐type specimens of *P. osborni* collected from the Lower and Middle Congo, and the holotype of *P. osborni* originating from Uele (Ubanghi) in the Middle Congo. As such, all the specimens from the Congo basin can be identified as *P. osborni*, as also previously reported by Konan et al. (2013). As a result, the holotype of *P. fasciaticeps* remains the only known specimen of this species, as no morphologically similar specimens, to our knowledge, have been found. The holotype represents a well‐distinct species as it differs from both *P. isidori* and *P. osborni*, two species to which it has been placed as a synonym and/or as subspecies in the past (Fricke et al., 2024), in several morphological characteristics (see Results for details).

### 4.3 Species complexes in need of further alpha‐taxonomic attention

Several nominal (sub)species of *Pollimyrus* have been synonymized with other species throughout the taxonomic history of the genus (Table 1). For a few of these, the holotypes of these synonymized species were available for study. This resulted in some new insights into their morphology and taxonomic validity.

Within *P. tumifrons*, there appears to be some morphological differentiation between the three previously synonymized species, with *P. tumifrons* and *P. anterodorsalis* resembling each other more than they each resemble *P. aequipinnis* (see Results). Unfortunately, Poll (1976) did not provide an explanation for the synonymization of *P. aequipinnis* with *P. tumifrons*. Non‐types from near the type locality of the latter species also resemble *P. tumifrons* and *P. anterodorsalis* more. It is therefore possible that both these identified morphotypes have large and, somehow, overlapping geographical distributions. However, to avoid more taxonomic confusion and possible nomenclatorial instability, *P. aequipinnis* is kept as a doubtful junior synonym of *P. tumifrons* until a further in‐depth comparative study, encompassing morphological, biogeographical, and, if possible, genetic data, clarifies this morphological group.

The present morphological and distribution data for *P. stappersii* and its subspecies *P. kapangae* seem to suggest that both might be valid species: *P. stappersii* itself and *P. kapangae*. The former was described from the Lukinda River basin, which is part of the Upper Congo basin. The latter subspecies was originally described as such by David (1935) from Shaba (now Katanga) in the Upper Congo basin based on three morphological differences in the following characteristics: number of anal‐fin rays (found here as well), number of lateral‐line scales (found here as well, but uncertainty remains), and the colouration (could not be verified due to discolouration in preservation). In addition to these, we also found a difference in the shape of the teeth. There were only a handful of specimens assigned to this species complex available for the study. However, none of these non‐type specimens originate from the same localities or habitat as the holotypes of both these current subspecies. Three specimens in the collections at RMCA were found near Lukonzolwa at Lake Mweru, which is potentially a different habitat close to the Lukinda River, the type locality of *P. s. stappersii*. No non‐type specimens of *P. s. kapangae* were known at the time of the study. Although two clear morphological differences were found, these preliminary results should be handled with care, considering the small amount of currently available specimens. Therefore, both species are here considered conspecific until more specimens become available for a more in‐depth integrative, comparative study, encompassing morphological, biogeographical, and genetic data.

The two syntypes of *P. adspersus* are reported to have been collected from West Africa (Günther, 1866). Unfortunately, no precise location is known for these specimens. *Pollimyrus petricolus* has also been reported from this part of the continent, that is, the upper and middle Niger (Bigorne, 2003), and many specimens found from the Senegal to the Niger and Chad basins have been assigned to the species *P. isidori* (Bigorne, 2003). Both these two species differ from *P. adspersus* morphologically and even belong to different morphological groups (see Results). As a result of the imprecise type locality of *P. adspersus* and the lack of a detailed morphological comparison of *Pollimyrus* species available till now, the species has been used as a waste‐basket taxon. Museum specimens caught from the Nilo‐Sudan IP (including the Volta River in Ghana), the Lower Guinea IP (e.g., Cross River and Wouri River in Cameroon) (Hopkins et al., 2007), and even the Congo IP (e.g., in Tshopo, Yangole, and Lindi rivers of the Middle Congo near Kisangani, and the towns of Boma and Matadi on the Lower Congo; see RMCA fish collection) have been identified as *P. adspersus*. A simple analysis of both syntypes and two non‐type specimens from the Zio River near the town of Lomé in Togo (RMCA fish collections), which were previously identified as *P. adspersus*, was performed. The two analysed specimens from the Zio River slightly differed morphologically from both the syntypes in body depth, head width, eye diameter, snout length, and caudal peduncle depth (see Supplementary file S2). This indicates that the taxonomy of the West African *Pollimyrus* requires further attention. However, due to a lack of specimens available during the study and as it fell outside the scope of this study, it was not possible to include an in‐depth comparison of West African *Pollimyrus* here. Other non‐type museum specimens were not included, as they first have to be reidentified to a valid or new species, potentially using the identification key for the type specimens provided (see Results). Those two non‐type specimens that were available during the time of the study were not further included, as there was no indication that they might represent novel distinct morphotypes, and thus potentially new species for science, although further study is required.

### 4.4 Limitations

Working only with the available type specimens in this case resulted in only having data of one or a few specimens for some of the taxa. Most species included in this study were described well over a century ago, often on one or a few type specimens only, and in several cases no material of the same locality is available. The resulting small sizes of the groups complicated, making definitive conclusions based solely on measurement data, as the observed ranges might not reflect the complete variation present in a taxon. The only remedy to this issue is to include more non‐type specimens in follow‐up studies once their species identification has been confirmed using the data presented here. As all species recognized here were differentiated from others by at least two morphological characteristics here, the limited sample sizes should not have impacted the results in a significant way. For those taxa, where there are only a few diagnostic or somewhat overlapping measurement ranges available, the remaining uncertainties were highlighted.

As this study only focused on available type specimens and did not include a molecular approach, the need for further consolidation of the present decisions and conclusions remains. Previous studies have shown that genetic analyses are highly needed to resolve species delineations, and cryptic species occur in this family (e.g., Rich et al., 2017; Sullivan et al., 2016). However, due to the complexity exactly, it remains pertinent to not lose track of morphological and geographical data either to validate a genetic specimen's taxon or to use all information available to identify and confirm identifications. In this study, several changes in the taxonomic status of species and some remaining issues and questions have been uncovered. Although these await confirmation and further study, respectively, with genetic analyses, the morphological synthesis provided here should be taken into consideration when working with *Pollimyrus* specimens.

With the present study mostly focusing on type specimens to delineate species and describe new species for science, more alpha‐taxonomical issues might be identified and in need to be solved when dealing with non‐type specimens. It is therefore recommended that any specimen currently available in collections and newly collected specimens should be (re‐)examined using the identification key. Only then will it be possible to better evaluate the geographical distribution of the 20 valid *Pollimyrus* species identified in the present paper. This will then also provide the much‐needed data to better evaluate the IUCN Red List conservation status and open the way to extend insights into their evolution and ecology.

### 4.5 Biogeography of the *Pollimyrus* species diversity

Based on the current study of most available type specimens, a first overview of the biogeography of the known *Pollimyrus* species has been summarized (Table 8). This overview is only preliminary and needs more verified non‐type specimen distribution data. Nevertheless, it shows that, with 11 species, the highest species diversity in *Pollimyrus* can be found in the Congo IP, whereas only a few species are found in the Nilo‐Sudan (two or three), Lower Guinea (two or three), Zambezi (three; but see Schedel et al., 2024 for a potential new species), East Coast (one), and Great Lakes (one) IPs. Except for their absence from the Angolan and Ethiopian Rift valley IPs, this known distribution for *Pollimyrus* coincides with that of the family Mormyridae as a whole (Lévêque & Paugy, 2017). Noticeable is also the dominance of slender‐tailed species in the Congolese IP, with seven(eight) slender‐tailed versus two(three) thick‐tailed species. In addition, slender‐tailed species only occur in the Congo IP, except for *P. adspersus*, whose original collecting locality remains unclear (Günther, 1866). The precise type locality of *P. adspersus* is unknown and therefore placed, tentatively, in three different IPs in western Africa. On the contrary, thick‐tailed species occur in most IPs from which *Pollimyrus* species have been reported, but only in low numbers.

**TABLE 8:** Distribution of the 20 valid *Pollimyrus* species and *Cyphomyrus plagiostoma* per ichthyogeographical province (IP) (sensu Lévêque & Paugy, 2017) based on the localities of type specimens only.

IP	Thick‐tailed morphological group	Slender‐tailed morphological group	Other morphological groups	Total
Nilo‐Sudan	*Pollimyrus petricolus*	*Pollimyrus adspersus?*	*Pollimyrus isidori* (thick tail)	2 or 3
Upper Guinea		*P. adspersus?*		0 or 1
Lower Guinea	*Pollimyrus guttatus*	*P. adspersus?*	*Pollimyrus vanneeri* (thick tail)	2 or 3
Congo	*Pollimyrus brevis* *Pollimyrus stappersii*	*Pollimyrus ibalazambai* *Pollimyrus maculipinnis* *Pollimyrus osborni* *Pollimyrus pedunculatus* *(Cyphomyrus plagiostoma)* *Pollimyrus pulverulentus* *Pollimyrus schreyeni*	*Pollimyrus fasciaticeps* (thick tail) *Pollimyrus tumifrons* (slender tail)	11
Zambezi	*Pollimyrus castelnaui* *Pollimyrus marianne* *Pollimyrus weyli*			3
East Coast	*Pollimyrus krameri*			1
Great Lakes			*Pollimyrus nigricans* (thick tail)	1

*Note*: Due to the unknown type locality of *P. adspersus* in “West‐Africa,” this species is tentatively placed in three IPs from this region.

In some species complexes and morphological groups, the lack of a clear morphological distinction between type specimens of nominal species complicated the species delineations. However, in some cases, multivariate statistical analysis (PCAs) detected subtle differences in morphology between (hydro)geographically distinctly occurring specimens. This was observed, for instance, in the *P. pulverulentus*, the *P. tumifrons*, and the *P. stappersii/P. castelnaui* species complexes (see Results). These examples show that the *Pollimyrus* alpha‐taxonomy might be strongly linked to allopatry, although, at present, the distribution ranges of several species in the Congo basin seem to overlap, such as those of *P. tumifrons*, *P. pulverulentus*, and *P. schreyeni* (Figure 2).

As this summary was compiled using mostly data from type specimens only and, as a result, does not take into account the full geographical distribution of the species identified, it could be possible that more species co‐occur in some of these IPs. So far, this is unknown, and each species seems to be restricted to one IP due to the low number of specimens available. Remarkably, *P. krameri* is the first *Pollimyrus* species described from the East Coast IP. So far, only *P. nigricans* is known to occur in one of the great lake basins in Africa, namely the mouth of the Katonga River at Lake Victoria (Great Lakes IP). As the type specimens from *P. isidori* and *P. nigricans* are morphologically similar and were found in the Nile River and Lake Victoria basin, respectively, two systems that are hydrologically connected, although the Ripon Falls and Owen Falls separate the Lake from the Nile basin (with presently the Nalubaale Dam), both these species could be phylogenetically closely related. Junior synonyms of *P. isidori* have been described from several river basins, *P. isidori gaillardi* from Lake Chad (Nilo‐Sudan IP), *P. isidori rudebeckii* from the Gambia River (Nilo‐Sudan IP), and *P. isidori vanderbilti* from the Central African Republic (Nilo‐Sudan/Congo IP). However, these junior synonyms were not included in the present morphological study, as the main scope of this study was to work with the valid species. Therefore, it remains unclear whether these junior synonyms are indeed conspecific with *P. isidori*. If correct, that would mean that the geographic range of *P. isidori* is the largest known range for a *Pollimyrus* species so far, and that one species can occur in multiple river basins and IPs. However, further study is needed to assess the conspecificity and re‐evaluate the distribution of these nominal species. It is to be noted that other nominal species once thought to be junior synonyms of *P. isidori* occurring in different IPs are now recognized as valid species, that is, *P. fasciaticeps* and *P. osborni* from the Congo IP.

The little amount of data on the distribution of species, combined with the small number of specimens known per species, which often come from the same river(basin), might indicate that *Pollimyrus* species, in general, are not very abundant. Due to their restricted distribution, they might be more vulnerable than more common and widespread species. However, for most species (15), the IUCN Red List (IUCN, 2024) categorized them as Least Concern (LC) (*P. adspersus, P. brevis, P. castelnaui, P. isidori* [including *P. osborni* and *P. fasciaticeps* as subspecies], *P. marianne, P. nigricans, P. nigripinnis, P. petricolus, P. pulverulentus, P. schreyeni, P. stappersii, P. tumifrons*, and *C. plagiostoma*), as there is little indication for any threats or population declines (Diouf, 2020; FishBase team RMCA and Geelhand, 2016; Moelants, 2010a, 2010b, 2010c, 2010d, 2010e, 2010f, 2010g, 2010h; Olaosebikan & Lalèyè, 2020; Olaosebikan & Moelants, 2020; Tweddle & Marshall, 2007; Tweddle et al., 2019), whereas three others remain Data Deficient (DD) (*P. pedunculatus, P. guttatus*, and *P. maculipinnis*: see Moelants, 2010i, 2010j, 2010k). The four new species were not assessed yet, but there are indications for severe environmental impacts on the type localities of *P. krameri* and *P. weyli* (R.B. personal communication). The available assessments, however, consider the range of the species based on identifications of non‐types (e.g., see presence of *P. adspersus* in the Congo basin), which are, most likely, considering our presented results, not using the correct species identifications (also see Hopkins et al., 2007). As such, the current assessments might not be reflective of the species vulnerability (see also Palacio et al., 2023). Similarly, the distribution details provided for each species on FishBase (Froese & Pauly, 2024) have been compiled from various existing publications. However, considering the numerous identification issues identified, the former distribution data should be handled with care. A revision of their current conservation status is certainly needed, which will only be possible after reidentifying museum specimens and summarizing the new resulting distribution for each of these.

### 4.6 Precarious mormyrid taxonomic knowledge: *Pollimyrus* as an illustrative case study

The Mormyridae, endemic to Africa and with 227 valid species known today (Fricke et al., 2024), is one of its important fish diversity components. Nevertheless, even during the past decade, new mormyrid species have been described on a regular basis, for example, *Petrocephalus petersi* (Kramer et al., 2012), *Pollimyrus cuandoensis* (Kramer et al., 2013), *Marcusenius kaninginii* (Kisekelwa et al., 2016), *Paramormyrops ntotom* (Rich et al., 2017), *Marcusenius wamuinii* (Decru et al., 2019), *Marcusenius verheyenorum* (Mambo et al., 2019), and *Cyphomyrus lufirae* (Mukweze Mulelenu et al., 2020). This, however, contrasts with our current overall alpha‐taxonomical, ecological, and other knowledge about mormyrid species diversity. For many taxa, both genera and species, a taxonomic revision is still lacking, genetic sampling has been limited, and ecological and behavioral, including EOD, information is often unavailable. The genus *Pollimyrus* and its species are no exception to this observation.

Taxonomic studies form the basis for many other kinds of knowledge, whether genetic, behavioral, evolutionary, ecological, etc., which could be combined in integrative studies. This makes these kinds of alpha‐taxonomic syntheses, even if based on type specimens alone, crucial for any further and future research on Mormyridae. The lack of alpha‐taxonomic knowledge and insights in the species distribution and ecology also makes it more difficult to effectively protect these species. Indeed, conservation actions are only possible based on sound knowledge of a species and its distribution. As this synthesis of *Pollimyrus* has also shown, the issue of generic delineations can still be problematic in Mormyridae, with several species having been the topic of reallocations in the past and present study. Furthermore, many unresolved issues, due to a lack of available non‐type specimens from certain type localities and regions, hinder the precise species delineations and the (re)evaluation of the status of junior synonyms and (sub)species. Therefore, *Pollimyrus*, with its now 20 recognized valid species, can serve as an example for the alpha‐taxonomic and generic issues in several mormyrid genera occurring across the African continent. Thus, research on much more type specimens is needed to come to a better overview of the alpha‐taxonomy of the more than 227 valid species, currently distributed across 22 genera (Fricke et al., 2024). As illustrated by the present revision of species within the genus *Pollimyrus*, many more hidden new species are probably to be discovered as well.

## 5 CONCLUSIONS

*Pollimyrus* is a species‐rich mormyrid genus with a known distribution across six ichthyogeographical provinces. A morphometric synthesis of all species in this genus resulted in (i) the confirmation of *C. plagiostoma* to *Cyphomyrus* and reallocation of *P. eburneensis* to *Paramormyrops*, (ii) the confirmation of *P. guttatus*, previously assigned to *Petrocephalus*, within *Pollimyrus*, (iii) a re‐evaluation of the generic characters and species status of all nominal species of the genus *Pollimyrus*, and (iv) the description of four new species for science, resulting in the identification of a total of 20 valid species in this genus. As this study allowed for a better understanding of the generic and morphometric species delineations, additional follow‐up studies will be able to provide a more profound understanding of the genetic diversity, geographic distribution, ecology, and behavior of these species. Furthermore, this synthesis has highlighted that certain (sub)basins, such as those in West Africa, situated within the known distribution area of the genus, remain highly understudied. If considering this case study on *Pollimyrus* as an illustrative example with broader implications for taxonomic diversity patterns within Mormyridae, it suggests that the species richness within this family is most likely still underestimated despite the significant taxonomic work conducted in certain genera and geographical regions, as several other mormyrid genera are still awaiting a similar, comprehensive synthesis.

## AUTHOR CONTRIBUTIONS

Katrien Dierickx: conceptualization, formal analysis, investigation, writing—original draft, writing—review and editing, visualization. Soleil Wamuini Lunkayilakio: resources, writing—review and editing. Roger Bills: resources, writing—review and editing. Emmanuel Vreven: conceptualization, writing—review and editing, supervision.

## Supporting information


**File S1.** Figures and additional results.

**File S2.** Raw data tables.

## Data Availability

Photographs of type specimens of the nominal species (Figures S1–S19), hydro‐geographic information of localities, additional results, and detailed and individual comparison of the species new for science with other species can be found in the Supplementary file S1. All raw data, containing the original measurements and meristics of each specimen, are available in the Supplementary file S2. X‐ray photographs of preserved specimens can be found on Zenodo following this link: 10.5281/zenodo.8135457.
