# Supplementary material for: Morphometric synthesis of Pollimyrus (Teleostei, Mormyridae) with the description of four new species
Source: J Fish Biol. 2024 Nov 24;106(3):846–92. doi: 10.1111/jfb.15983 (PMC11949749; doi:10.1111/jfb.15983)
Supplement: Supplementary file 1 — File S1. Figures and additional results. [file JFB-106-846-s002.docx]

**Supplementary Information - S1**

Morphometric synthesis of *Pollimyrus* (Teleostei, Mormyridae) with the description of four new species

**Authors:** Katrien DIERICKX^1,*^; Soleil WAMUINI LUNKAYILAKIO^2^; Roger BILLS^3^; Emmanuel VREVEN^4^

^1^Department of Archaeology and Cultural History, NTNU University Museum, Norwegian University of Science and Technology (NTNU), Trondheim, Norway

^1,4^Ichthyology, Section Vertebrates, Department Biology, Royal Museum for Central Africa (RMCA), Tervuren, Belgium

^2^Institut Supérieur Pédagogique (ISP) de Mbanza-Ngungu, Kongo Central, République Démocratique du Congo

^3,4^South African Institute for Aquatic Biodiversity (SAIAB), Makhanda, South Africa

*Corresponding author: [katrien.dierickx.icht@gmail.com](mailto:katrien.dierickx.icht@gmail.com)

^2^Email: [s.wamuini@gmail.com](mailto:s.wamuini@gmail.com)

^3^Email: [ir.bills@saiab.nrf.ac.za](mailto:IR.Bills@saiab.nrf.ac.za)

^4^Email: [emmanuel.vreven@africamuseum.be](mailto:emmanuel.vreven@africamuseum.be)

ORCID: KD 0000-0002-9028-7652; SW 0000-0003-1448-3595; RB 0000-0001-6034-4196; EV 0000-0002-2503-4712

This paper and the nomenclatural act(s) it contains have been registered in ZooBank (www.zoobank.org), the official register of the International Commission on Zoological Nomenclature. The LSID (Life Science Identifier) number of the publication is: urn:lsid:zoobank.org:pub:CF9E11C3-BDC3-4F3F-A9F8-5BD25607CD54.

##

## Photos of preserved specimens

In order of appearance in the result section of the main text.


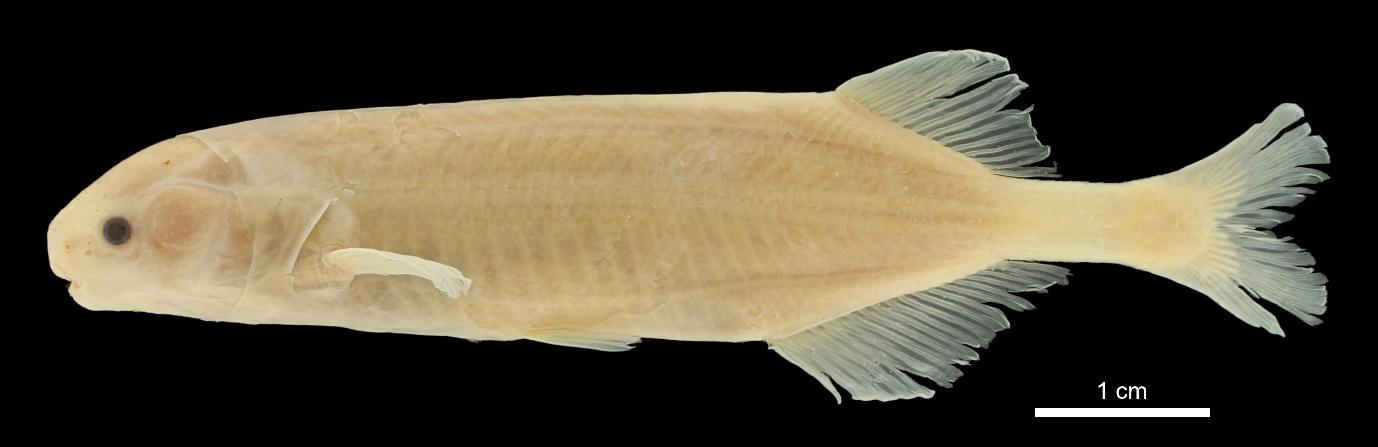

**Figure S1.** Photograph of preserved holotype of *P. eburneensis* (MNHN 1990.0376: 72.94 mm SL). Scale bar is 1 cm.


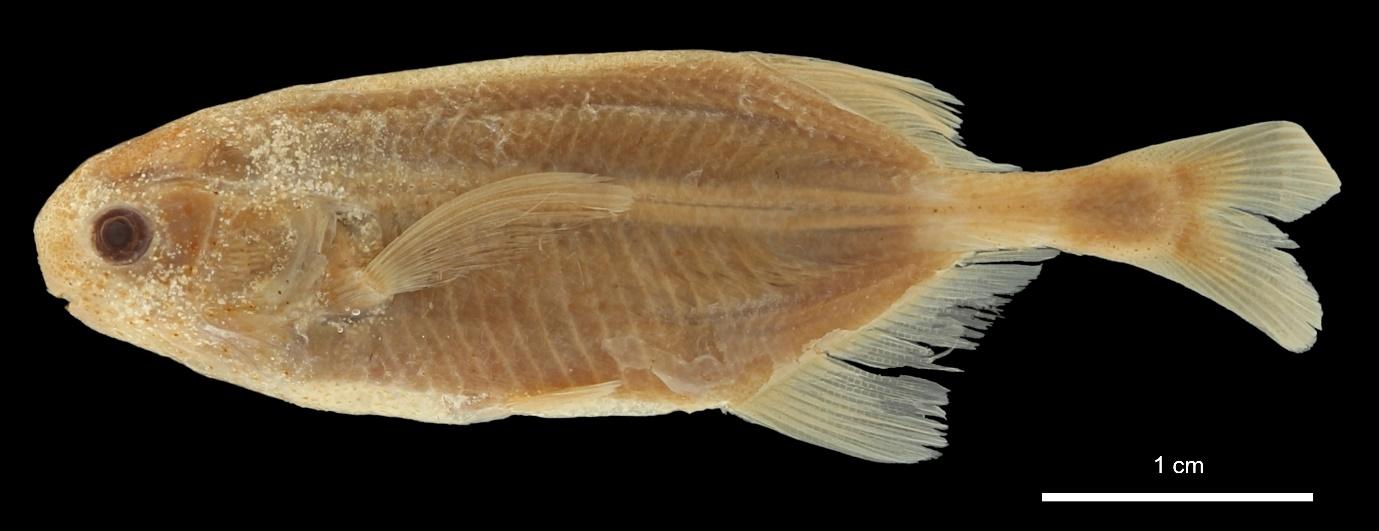

**Figure S2.** Photograph of preserved holotype of *P. guttatus* (ANSP 65504: 39.54 mm SL). Scale bar is 1 cm.


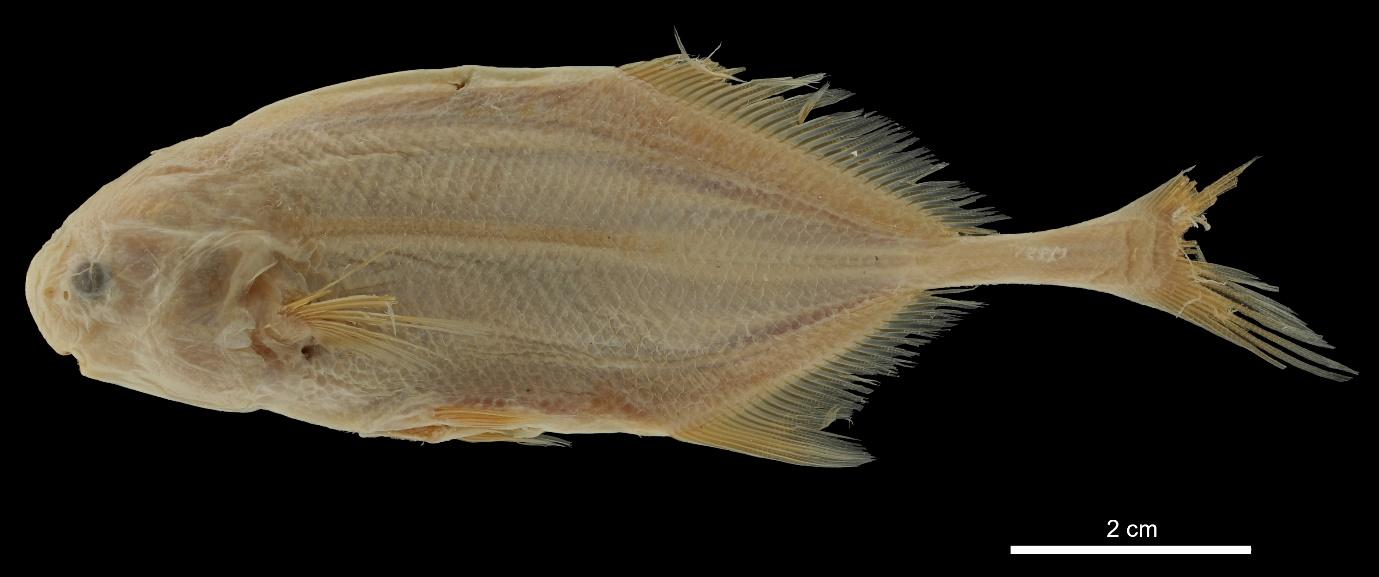

**Figure S3.** Photograph of preserved syntype of *C. plagiostoma* (BMNH 1898.11.12.13-14: 88.43 mm SL). Scale bar is 2 cm.


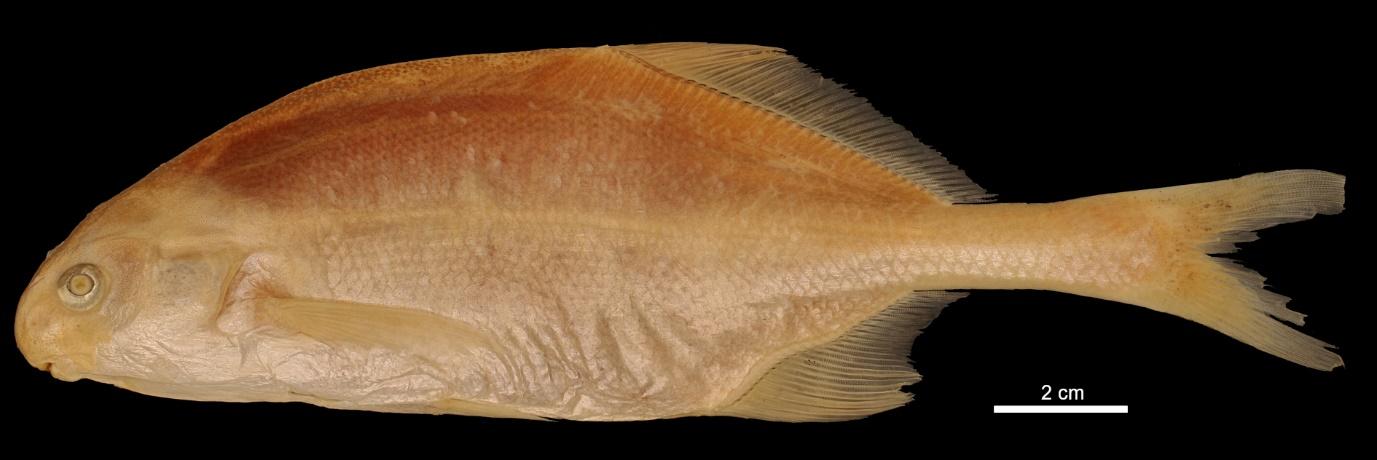

**Figure S4.** Photograph of preserved syntype of *C. petherici* (BMHN 1862.6.17.92: 175.34 mm SL). Scale bar is 2 cm.


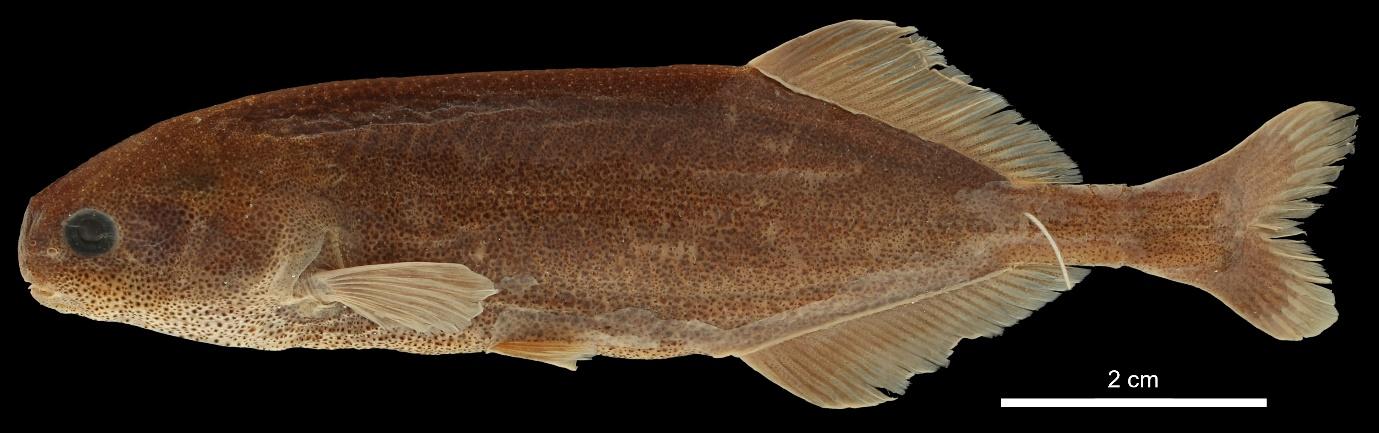

**Figure S5.** Photograph of preserved holotype of *P. petricolus* (MNHN 1954.0008: 87.85 mm SL). Scale bar is 2 cm. A piece of string from the label is still visible at the tail.


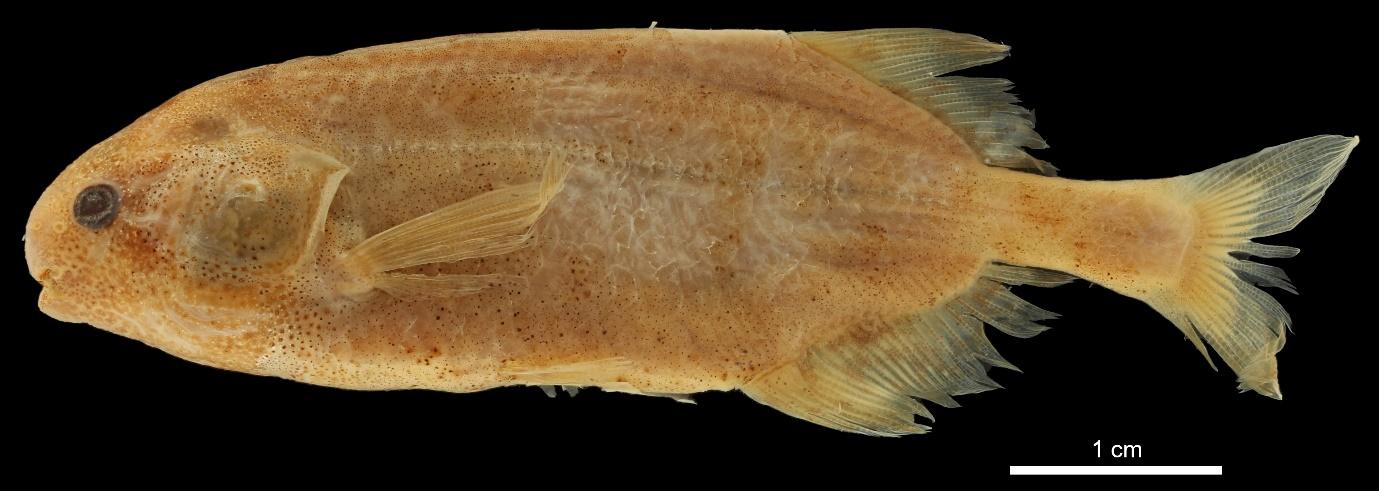

**Figure S6.** Photograph of preserved syntype of *P. brevis* (RMCA P.1804: 53.96 mm SL). Scale bar is 1 cm.


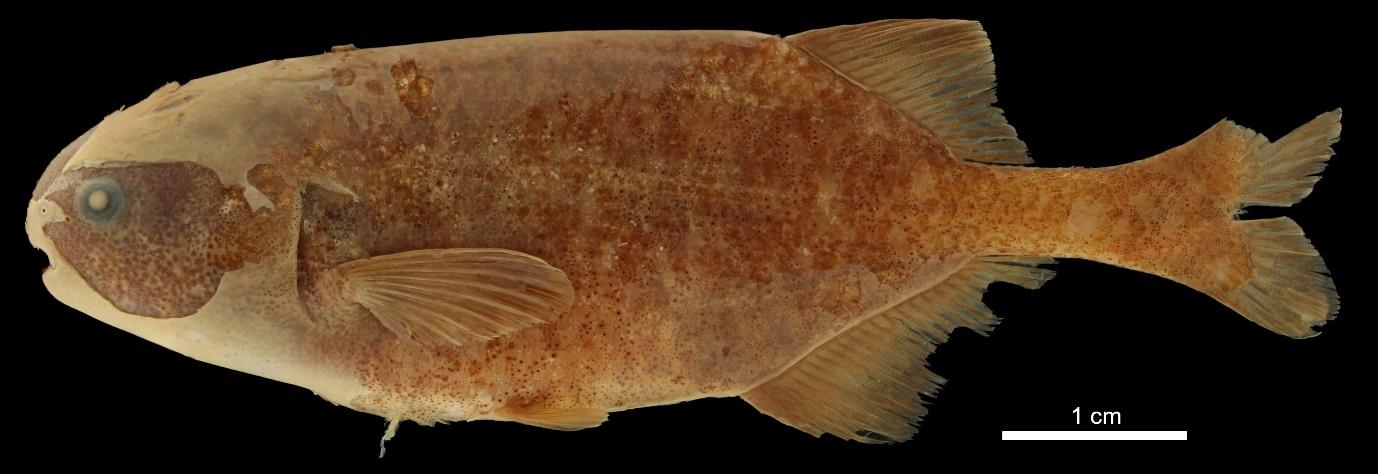

**Figure S7.** Photograph of preserved syntype of *P. castelnaui* (BMNH 1910.5.31.11-12 ID=2: 58.32 mm SL). Scale bar is 1 cm.


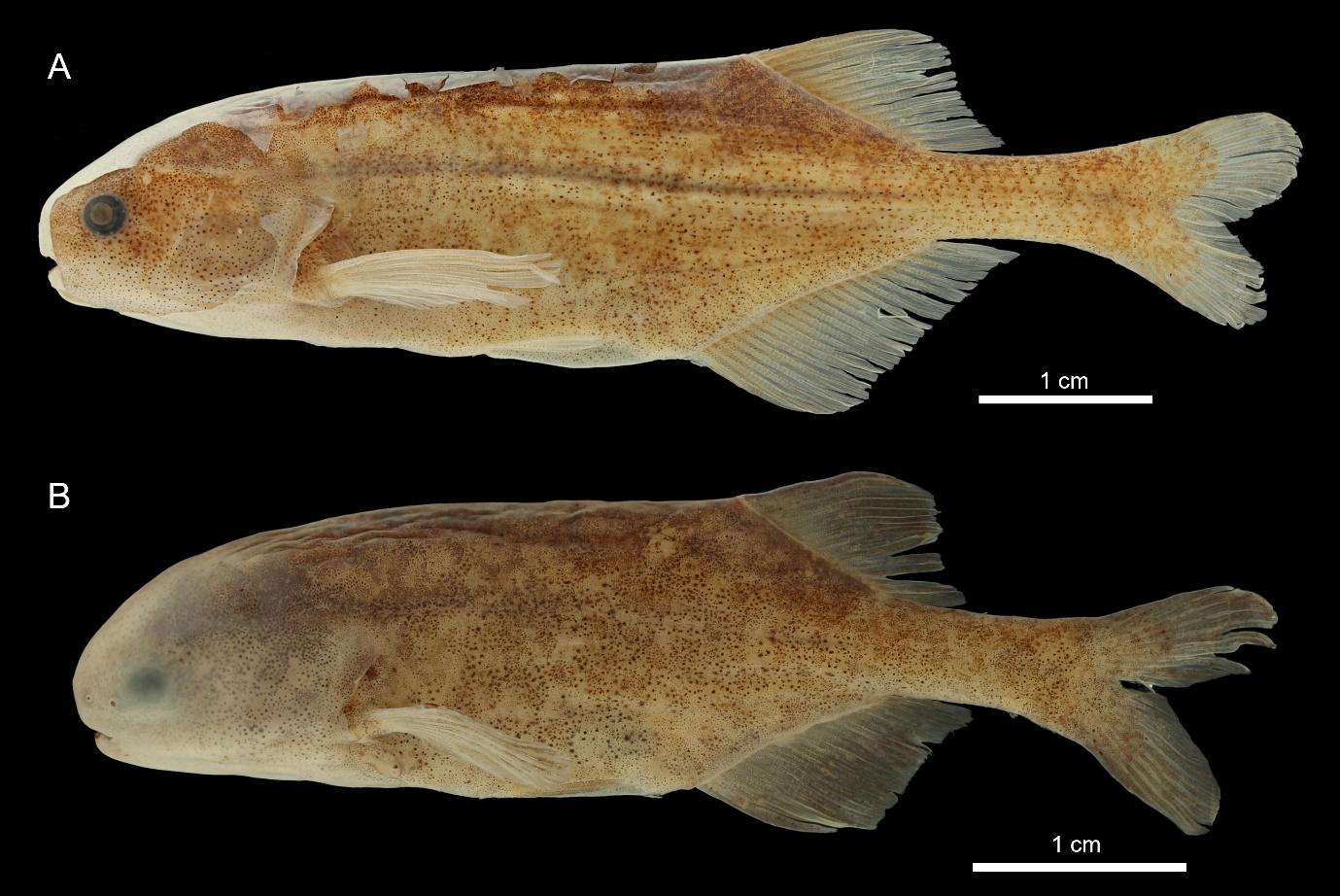

**Figure S8.** Photograph of preserved specimens of *P. marianne* and *P. cuandoensis*. A. Holotype of *P. marianne* (SAIAB 66943: 63.92 mm SL). B. Holotype of *P. cuandoensis* (ZSM 41805: 44.11 mm SL). Scale bars are 1 cm.


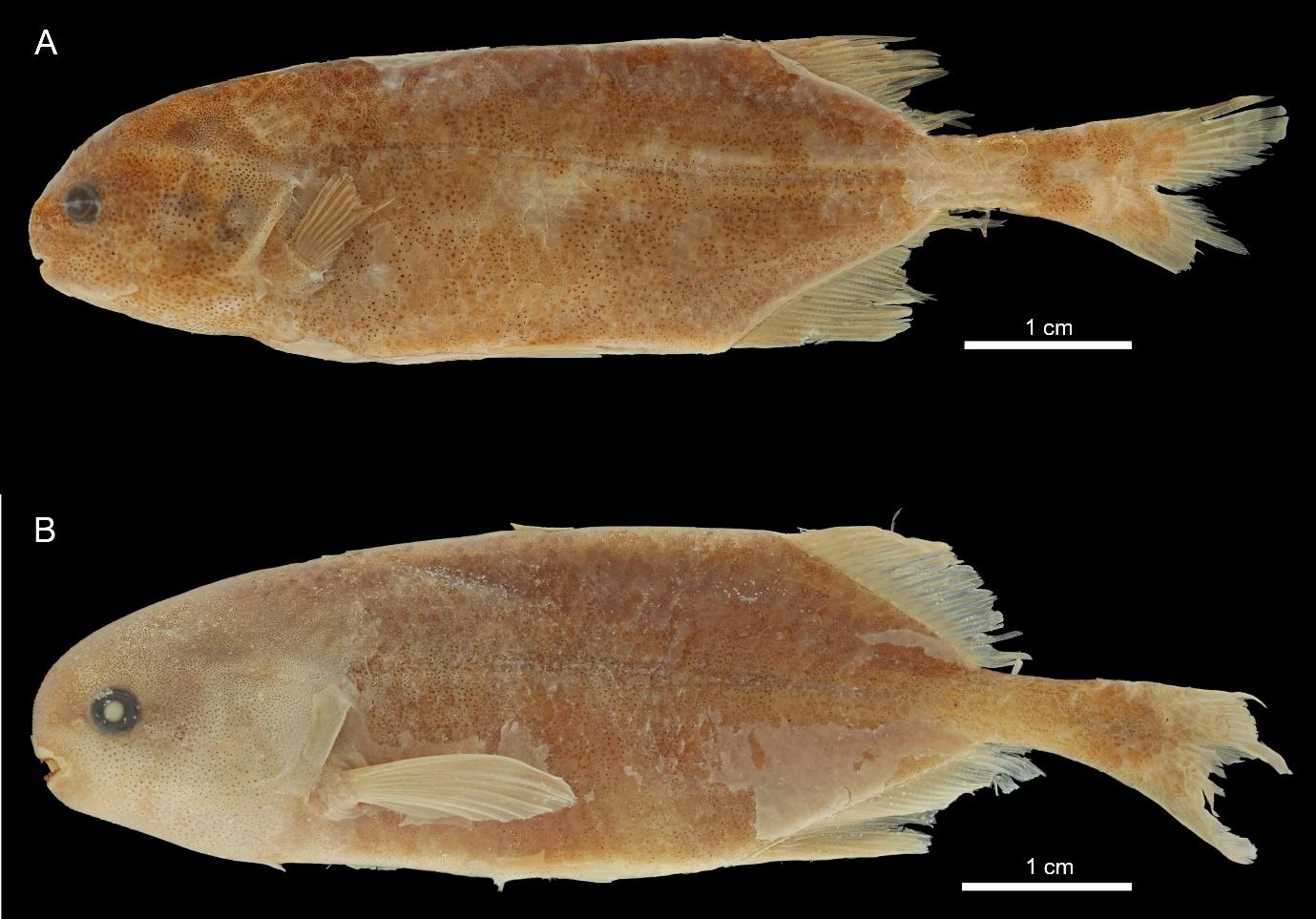

**Figure S9.** Photograph of preserved specimens of *P. stappersii.* A. Holotype of *P. stappersii* (RMCA P.12689: 59.28 mm SL). B. Holotype of the subspecies *P. s. kapangae* (RMCA P.39492: 61.61 mm SL). Scale bars are 1 cm.


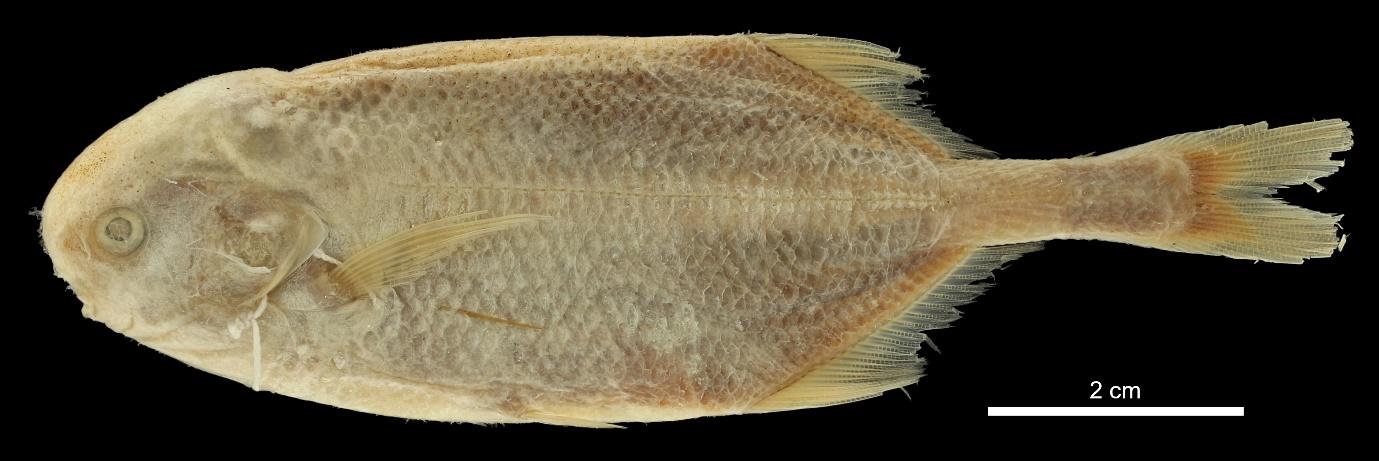

**Figure S10.** Photograph of preserved holotype of *P. isidori* (MNHN IC-4209: 84.33 mm SL). Scale bar is 2 cm.


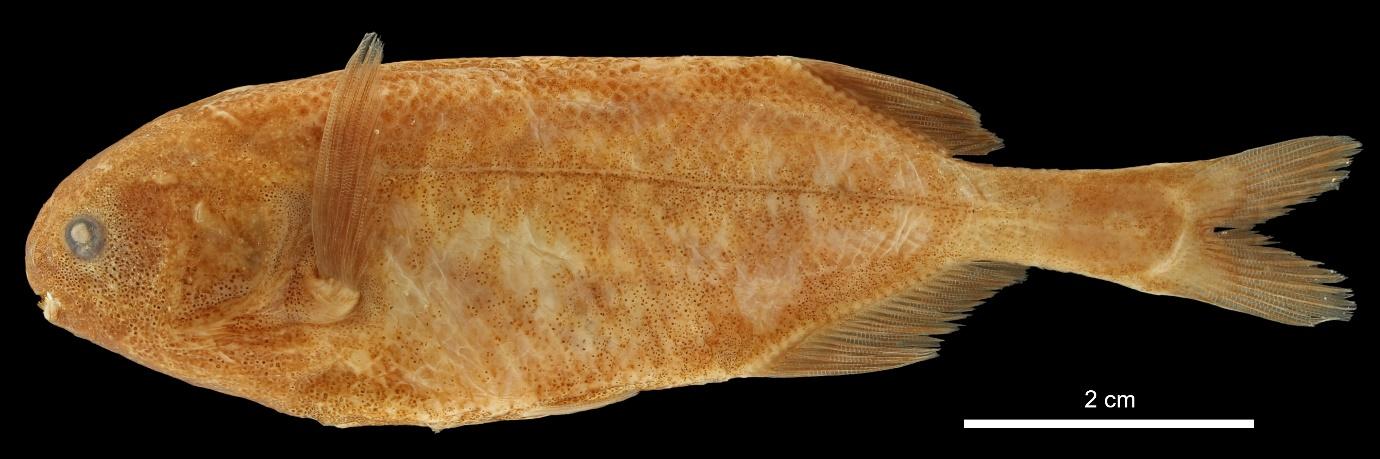

**Figure S11.** Photograph of preserved syntype of *P. nigricans* (BMNH 1906.5.30.85-94: 76.88 mm SL). Scale bar is 2 cm.


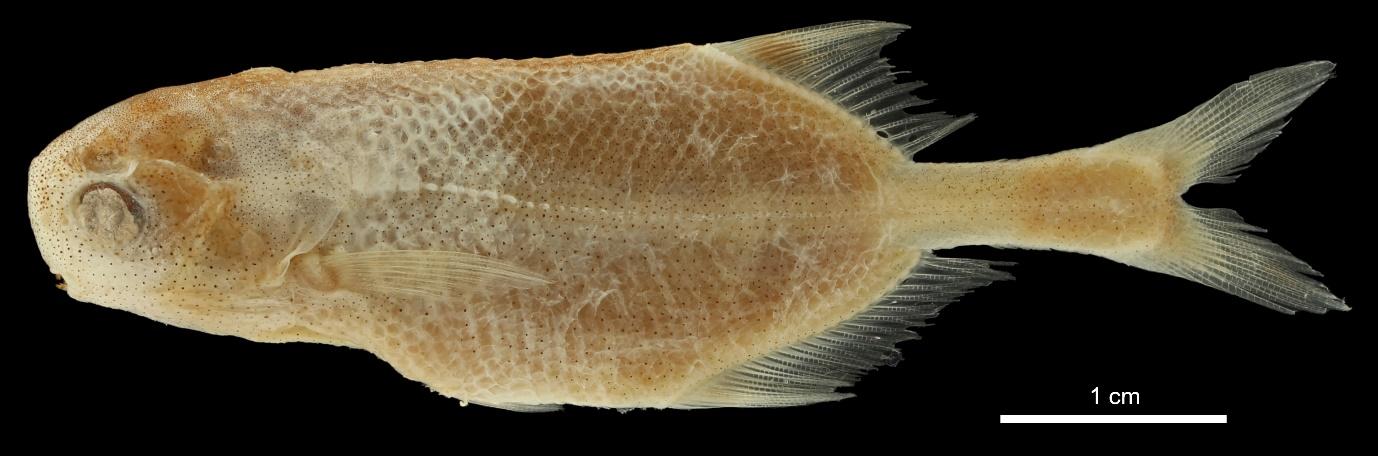

**Figure S12.** Photograph of preserved holotype of *P. fasciaticeps* (RMCA P.7243: 49.29 mm SL). Scale bar is 1 cm.


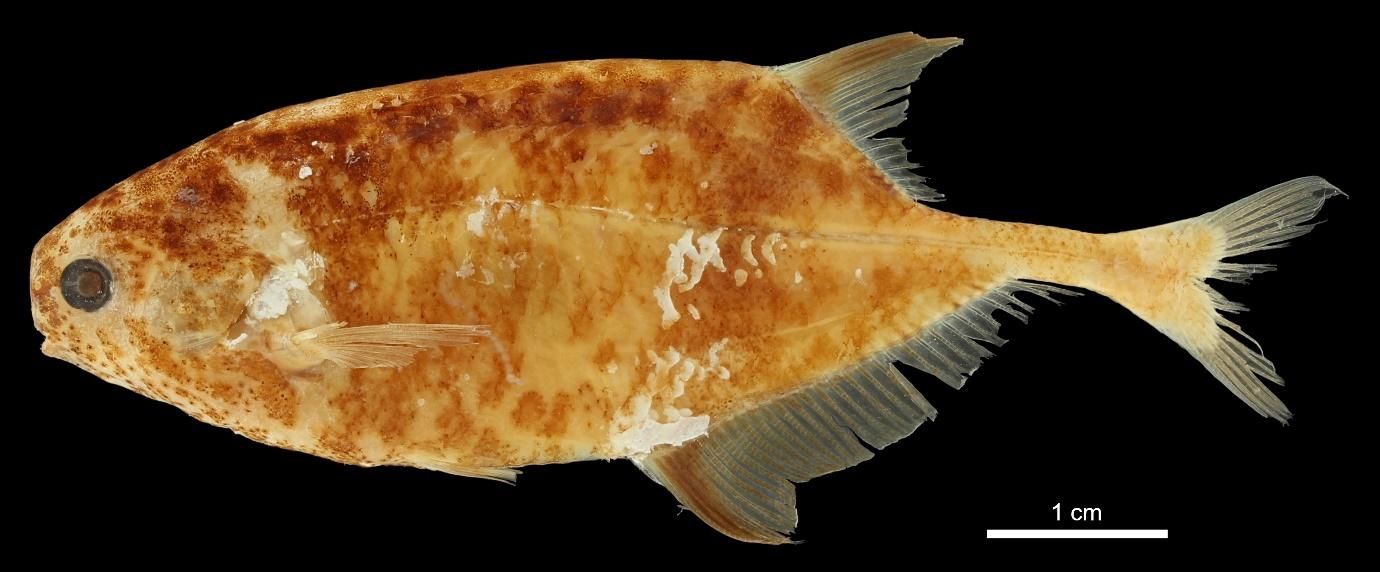

**Figure S13.** Photograph of preserved holotype of *P. schreyeni* (RMCA P.174708: 70.49 mm SL). Scale bar is 1 cm.


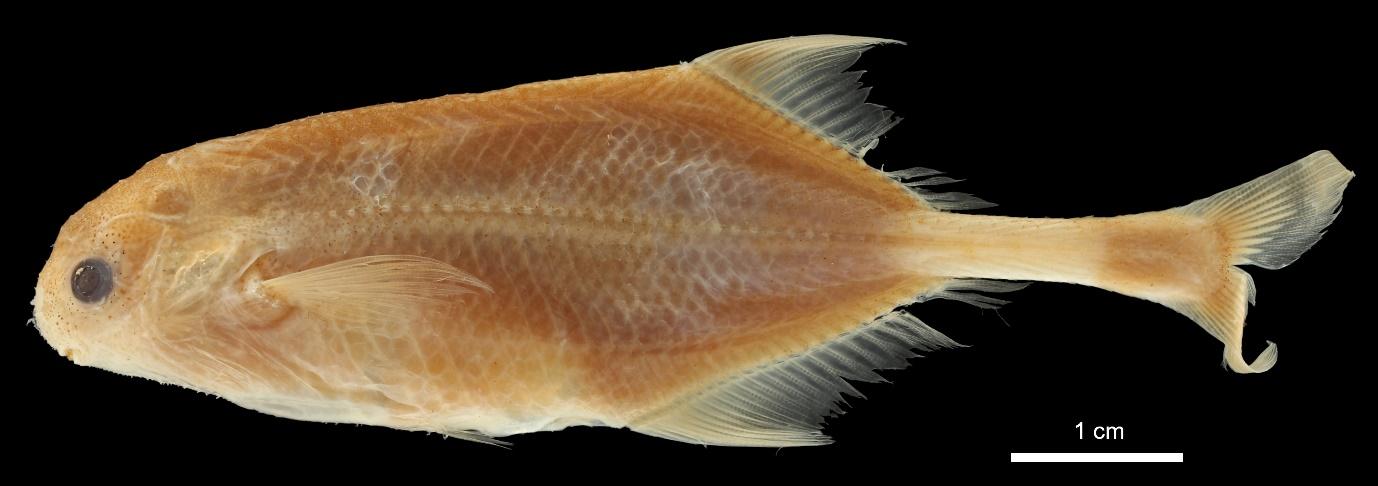

**Figure S14.** Photograph of preserved holotype of *P. pedunculatus* (RMCA P.22664: 67.69 mm SL). Scale bar is 1 cm.


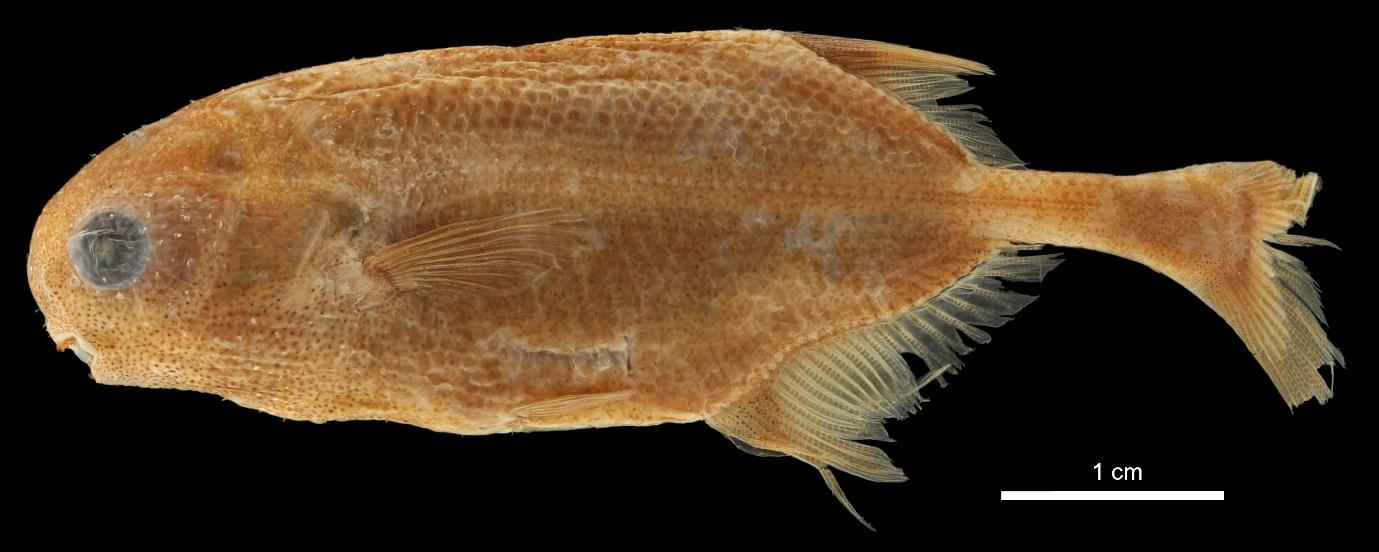

**Figure S15.** Photograph of preserved holotype of *P. maculipinnis* (AMNH I-12355: 52.92 mm SL). Scale bar is 1 cm.


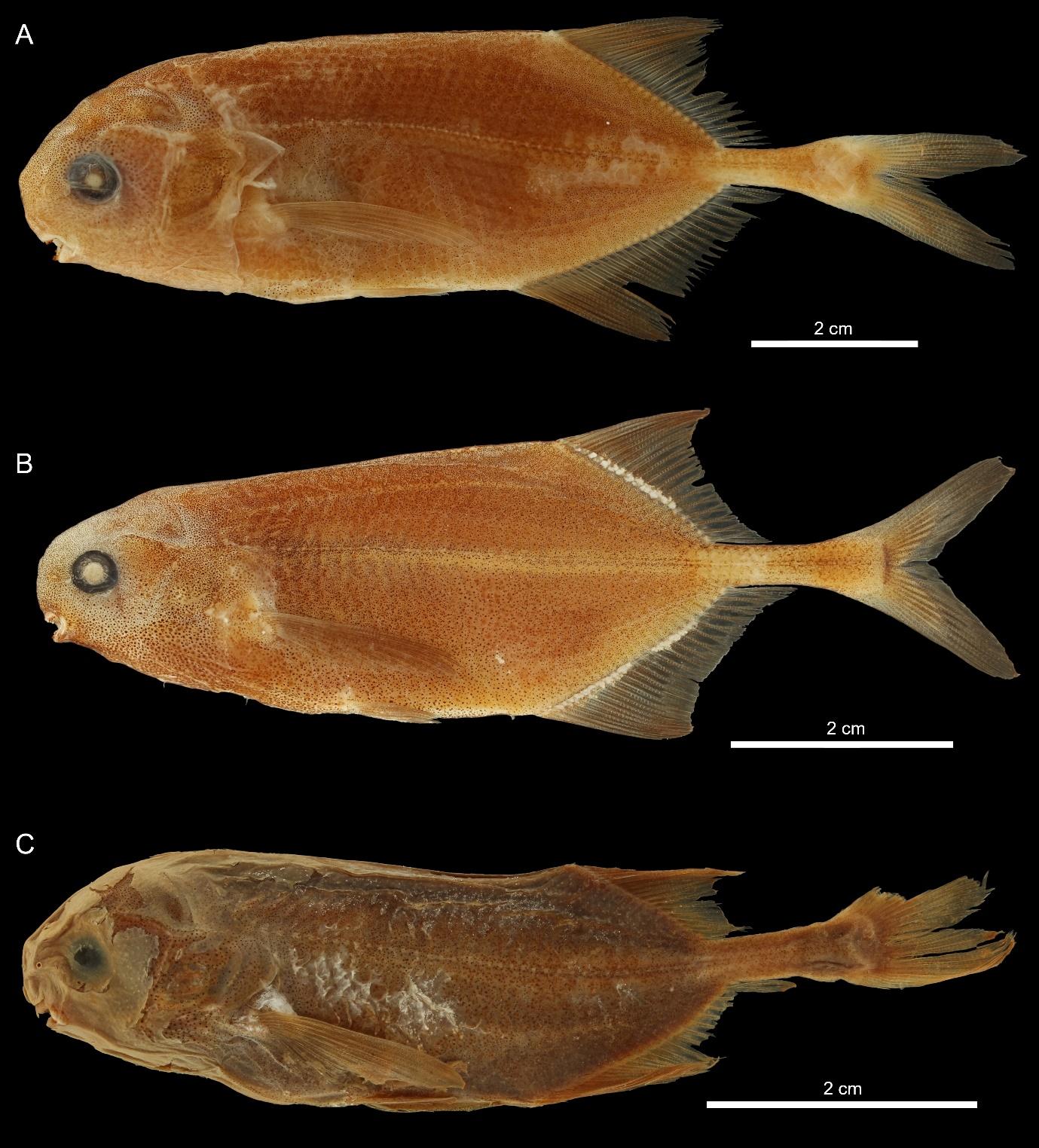

**Figure S16.** Photographs of preserved specimens of *P. pulverulentus* and *P. nigripinnis*. A. Syntype of *P. pulverulentus* (BMNH 1899.9.26.26-27: 95.21 mm SL)*.* B. Syntype of *P. nigripinnis* from Kutu (BMNH 1899.9.26.28-29: 72.47 mm SL)*.* C. Syntype of *P. nigripinnis* from Uéré (RMCA P.344: 61.28 mm SL). Scale bars are 2 cm.


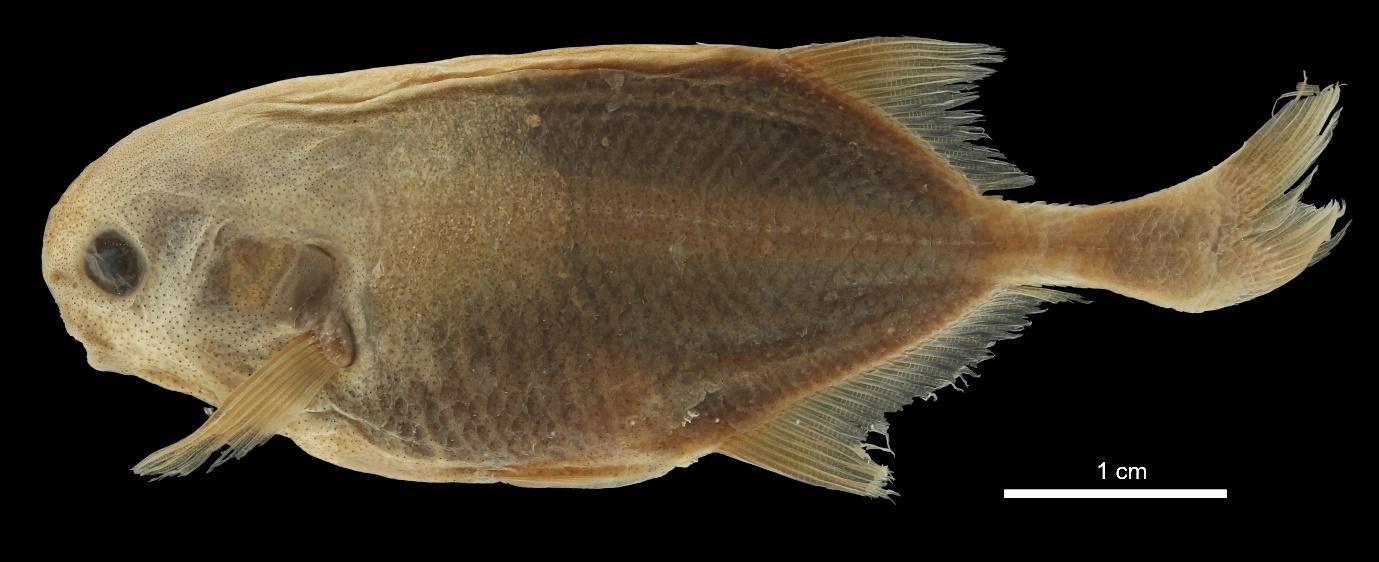

**Figure S17.** Photograph of preserved holotype of *P. osborni* (AMNH I-6934: 53.00 mm SL). Scale bar is 1 cm.


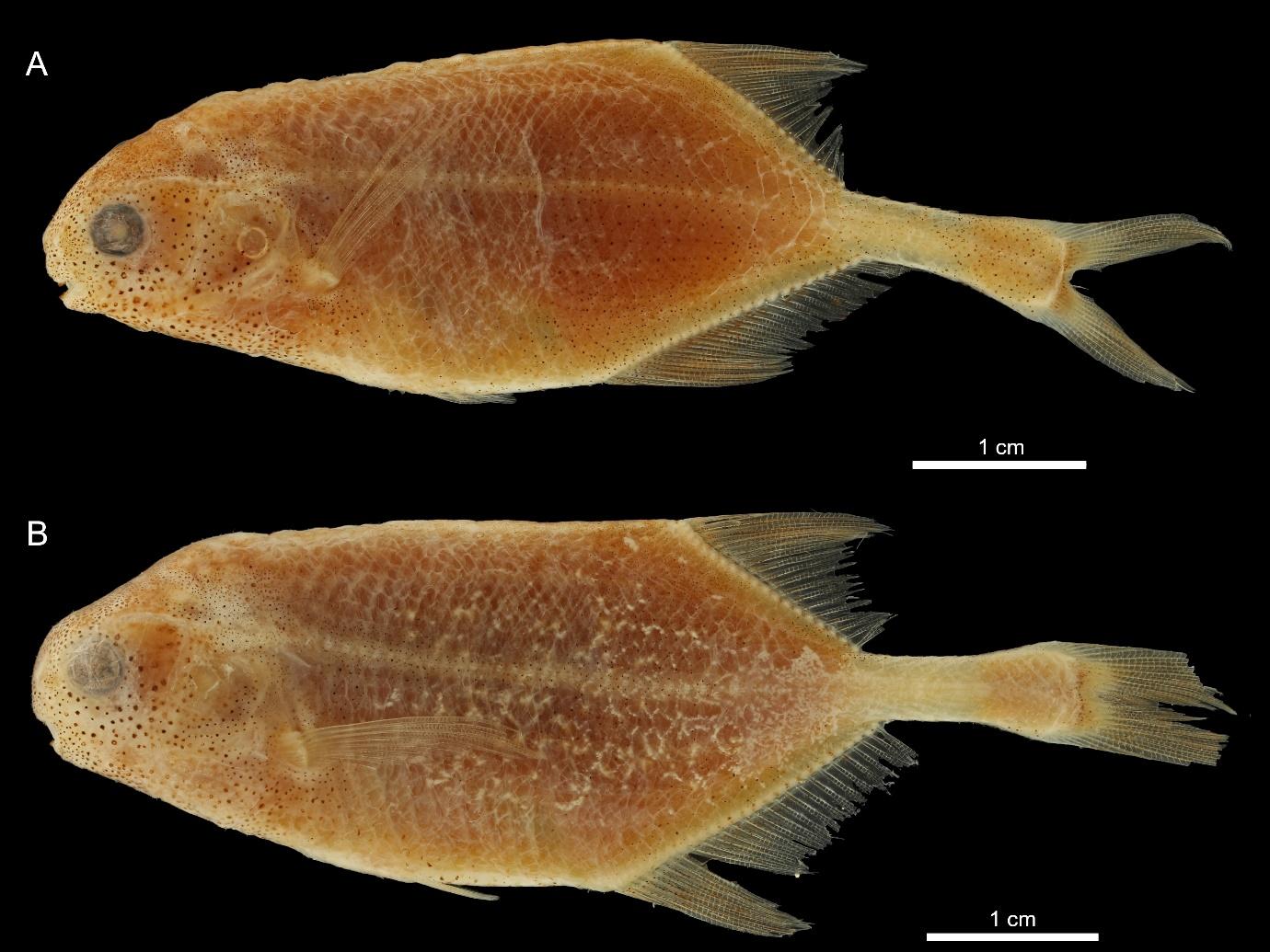

**Figure S18.** Photograph of preserved syntypes of *P. adspersus.* A. BMNH 1865.5.3.41: 57.94 mm SL; B. BMNH 1865.5.3.41: 58.88 mm SL. Scale bars are 1 cm.


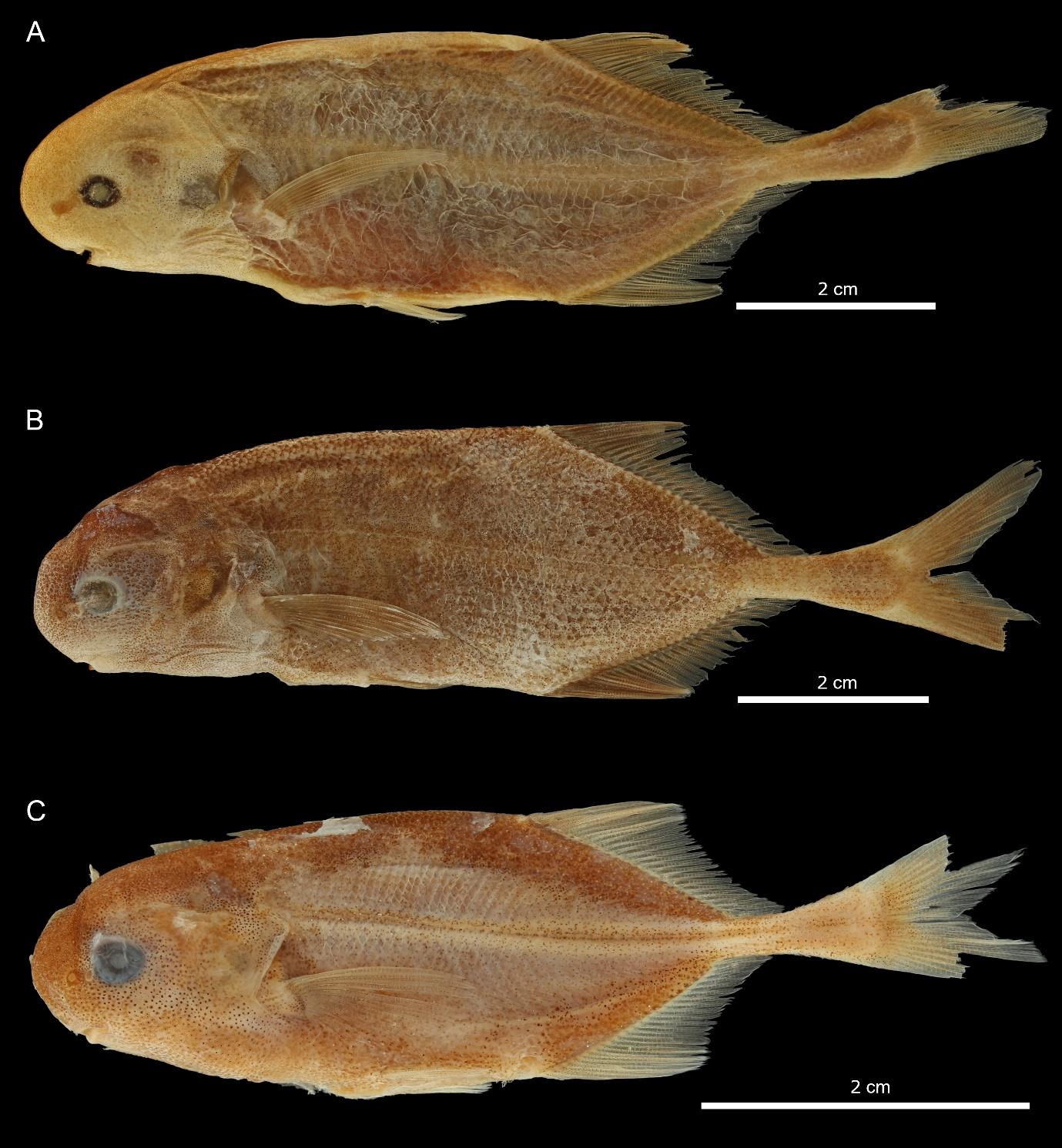

**Figure S19.** Photograph of preserved specimens of *P. tumifrons.* A. Holotype of *P. tumifrons* (RMCA P.1160: 88.55 mm SL) B. Holotype of the subspecies *P. aequipinnis* (RMCA P.15188: 90.07 mm SL)*.* C. Holotype of the subspecies *P. anterodorsalis* (RMCA P.22167: 44.76 mm SL). Scale bars are 2 cm.

##

## River Basin information

The hydro-geographical location of each river, lake or basin mentioned in the text can be found below. The hydro-geographical systems are listed alphabetically. For each system, the ichthyological province following Lévêque & Paugy (2017) and the mouth or the river it flows into is mentioned.

Agnébi River: Nilo-Sudan IP; affluent of the Ebrié Lagoon.

Aruwimi River: Congo IP; right bank affluent of the Middle Congo.

Bomokandi: Congo IP; left bank affluent of the Uelé River.

Buzi River: Zambezi IP; mouth at Mozambique Channel.

Congo River: main river in Congo IP; Upper Congo from source to Boyoma Falls, Middle Congo from Boyoma Falls to Kinshasa, Lower Congo from Kinshasa to river mouth at Atlantic coast.

Cross River: Lower Guinea IP; mouth at Atlantic coast.

Fimi River: Congo IP; right bank affluent of the Kwa River

Gambia River: Nilo-Sudan IP; mouth at Atlantic coast.

Kasaï River: Congo IP; left bank affluent of the Middle Congo.

Katonga River: Great Lakes IP; between Lake George and Lake Victoria, drains into the latter.

Kineke River: Lower-Guinea IP; mouth at Atlantic Ocean.

Kissafou River: Lower-Guinea IP; right bank affluent of the Kouilou-Niari River.

Kouilou-Niari River: Lower Guinea IP; mouth at Atlantic coast.

Krongwa Stream: Zambezi IP; small stream flowing into the Mussapa River.

Kwa River: Congo IP; left bank affluent of the Middle Congo River.

Kwando River (Cuando River): Zambezi IP; right bank affluent of the Zambezi River; known as Chobe and Linyanti Rivers.

Kwanza River: Angolan IP; mouth at Atlantic Ocean.

Lac Bleu: Congo IP; Léfini Basin.

Lagos Lagoon: Nilo-Sudan IP; mouth at Atlantic Ocean.

Lake Chad: Nilo-Sudan IP: endorheic lake.

Lake Mai Ndombe: Congo IP; drains into the Fimi River.

Lake Ngami: Zambezi IP; endorheic lake.

Léfini River: Congo IP; right bank affluent of the Middle Congo.

Lindi River: Congo IP; right bank affluent of the Middle Congo.

Luachimo River: Congo IP; left bank affluent of the Kasaï River.

Lualaba River: Congo IP; Upper Congo.

Lucite River: Zambezi IP; left bank affluent of the Buzi River.

Lugenda River: East Coast IP; right bank affluent of the Rovuma River.

Lukinda River: Congo IP; affluent of Mweru Lake.

Lufira River: Congo IP; right bank affluent of the Lualaba River.

Luki River: Congo IP; left bank affluent of Lukunga River.

Lukunga River: Congo IP; right bank affluent of the Lower Congo.

Lulua River: Congo IP; right bank affluent of the Kasaï River.

Mukombe Stream: Zambezi IP; small stream flowing into the Lucite River.

Mussapa River: Zambezi IP; left bank affluent of the Lucite River.

Niger River: Nilo-Sudan IP; mouth at Atlantic Ocean.

Nile River: Nilo-Sudan IP; mouth at Mediterranean Sea.

Nkupo Stream: East Coast IP; small stream flowing into Lugenda River.

Ogooué River: Lower Guinea IP; mouth at Atlantic Ocean.

Okavango River: Zambezi IP; drains into the Okavango Delta.

Pool Malebo: Congo IP; border between Middle and Lower Congo.

Rovuma River: East Coast IP; mouth at Indian Ocean.

Senegal River: Nilo-Sudan IP; mouth at Atlantic Ocean.

Tshopo River: Congo IP; left bank affluent of the Lindi River.

Tshuapa River: Congo IP; confluence with Lomela River to Busira River, and later Ruki River, a left affluent of the Middle Congo.

Ubangi River: Congo IP; right bank affluent of Middle Congo.

Uelé River: Congo IP; affluent of the Ubangi River.

Uéré River: Congo IP; right bank affluent of the Uele River.

Volta River: Lower-Guinea IP; mouth at Atlantic Ocean.

Wouri River: Lower-Guinea IP; mouth at Atlantic Ocean.

Zambezi River: main river in Zambezi IP; mouth at Indian Ocean.

Zio River: Nilo-Sudan IP; mouth at Atlantic Ocean.

## Extended diagnoses of species new to science

In this section the species newly described to science are compared individually with all other *Pollimyrus* species regarded as valid in this study. A general diagnosis for each newly described species can be found in the main text.

***Pollimyrus ibalazambai* sp. nov.**Figures 8 and 9, Tables 6 and 7
urn:lsid:zoobank.org:act:EA58D11A-D2BB-43E4-982B-4A461985C857

*Pollimyrus ibalazambai* sp. nov. differs from *P. brevis*, *P. castelnaui*, *P. cuandoensis*, *P. fasciaticeps*, *P. guttatus*, *P. isidori*, *P. marianne,* *P. nigricans*, *P. petricolus*, *P. stappersii*, *P. vanneeri* sp. nov., *P. weyli* sp. nov. , and *P. krameri* sp. nov., by having a slenderer tail (15.3-19.2% BD vs. 20.3-31.2% BD). It further differs from *P. brevis*, *P. castelnaui*, *P. cuandoensis*, *P. guttatus*, *P. marianne,* *P. petricolus*, *P. stappersii*, *P. weyli* sp. nov. , and *P. krameri* sp. nov., by having a blocky snout (vs. rounded snout), pointed pectoral fin (vs. rounded), concave anal and dorsal fins (vs. rounded), and by lacking a chin (vs. obvious chin visible).

In addition, it further differs from *P. fasciaticeps* alone by having a longer pectoral fin (23.1-27.5% SL vs. 21.3% SL), more anal fin rays (25-28 vs. 24), and fewer caudal peduncle scales (12-15 vs. 18).

It differs from *P. isidori* alone by a shorter preanal length (55.5-59.1% SL vs. 62.7% SL), a shorter prepelvic length (37.0-40.7% SL vs. 44.2% SL), a longer pectoral fin (23.1-27.5% SL vs. 20.7% SL), a shorter pectoral-pelvic distance (16.1-18.6% SL vs. 21.4% SL), and more anal fin rays (25-28 vs. 24).

It differs from *P. nigricans* alone by having a longer anal fin (28.1-32.1% SL vs. 21.0-24.6% SL), a longer posterodorsal length (40.3-45.9% SL vs. 35.5-38.7% SL), a larger eye (23.8-27.3% HL vs. 15.3-19.0% HL), a shorter postorbital length (54.5-58.2% HL vs. 62.4-67.3% HL), more anal fin rays (25-28 vs. 22-24), and fewer caudal peduncle scales (12-15 vs. 17-20).

It differs from *P. vanneeri* sp. nov. alone by having a slenderer tail (15.3-19.2% BD vs. 20.3-24.2% BD), a wider interorbital width (36.2-46.8% HL vs. 26.9-34.4% HL), a pointed and longer pectoral fin (vs. rounded; 23.1-27.5% SL vs. 17.7-22.7% SL), fewer lateral line scales (50-59 vs. 59-73), and fewer caudal peduncle scales (12-15 vs. 17-23).

*Pollimyrus ibalazambai* sp. nov. differs from *P. adspersus* by having small teeth and by having more embedded and symmetrical teeth on the lower jaw, compared with large, asymmetrical teeth. In *P. ibalazambai* sp. nov. the first tooth on the upper jaw is positioned more anteriorly than the other teeth, while in *P. adspersus* the first tooth is positioned at the same level or has an extra tooth right behind the first one.

*Pollimyrus ibalazambai* sp. nov. differs from *P. maculipinnis* by having a longer dorsal fin (20.4-23.9% SL vs. 19.0-19.5% SL), a longer pectoral fin (23.1-27.5% SL vs. 19.8-22.0% SL), a shorter head (20.9-22.8% SL vs. 25.3% SL), a longer internasal length (8.1-10.1% HL vs. 6.8-7.7% HL), and more anal fin rays (25-28 vs. 24).

*Pollimyrus ibalazambai* sp. nov. differs from the *P. nigripinnis*-complex by having a shorter prepectoral distance (22.5-25.6% SL vs. 26.6-30.2% SL), a shorter head (20.9-22.8% SL vs. 23.5-27.7% SL). The teeth of *P. ibalazambai* sp. nov. have large and obvious cusps, whereas those of the *P. nigripinnis*-complex have very small and hard to distinguish cusps.

*Pollimyrus ibalazambai* sp. nov. differs from *P. osborni* by having a shorter prepelvic distance (37.0-40.7% SL vs. 42.2% SL), a shorter head (20.9-22.8% SL vs. 23.7% SL), a slenderer lower jaw (13.9-16.8% HL vs. 18.3% HL), a broader upper jaw (15.7-19.9% HL vs. 14.4% HL), more dorsal fin rays (17-21 vs. 16), more anal fin rays (25-28 vs 24), and fewer caudal peduncle scales (12-15 vs. 17). Its teeth are small and spaced apart from each other, whereas in *P. osborni* they are large and long, and closely packed together.

*Pollimyrus ibalazambai* sp. nov. differs from *P. pedunculatus* by having a longer predorsal length (60.3-5.3% SL vs. 58.6% SL), a longer preanal length (55.5-59.1% SL vs. 52.1% SL), a longer prepelvic length (37.0-40.7% SL vs. 34.3% SL), a shorter dorsal fin (20.4-23.9% SL vs. 25.0% SL), a longer pectoral fin (23.1-27.5% SL vs. 20.9% SL), a shorter caudal peduncle (16.6-19.5% SL vs. 22.8% SL), and a longer belly (17.3-19.9% SL vs. 15.4% SL), a larger eye (23.8-27.3% HL vs. 19.3% HL). The anterior nostril is positioned higher than the posterior one in *P. ibalazambai* sp. nov., whereas vice versa in *P. pedunculatus*.

*Pollimyrus ibalazambai* sp. nov. differs from *C. plagiostoma* by having a longer predorsal length (60.3-65.3% SL vs. 54.7-58.5% SL), a shorter dorsal fin (20.4-23.9% SL vs. 34.4-37.9% SL), a shorter pelvic fin (8.4-11.1% SL vs. 12.5-13.4% SL), a shorter posterodorsal length (40.3-45.9% SL vs. 51.4-52.8% SL), a longer internasal distance (8.1-10.1% HL vs. 7.2-7.8% SL), a larger eye (23.8-27.3% HL vs. 19.1-19.3% HL), a shorter snout (11.4-16.7% HL vs. 18.7-22.0% HL), fewer dorsal fin rays (17-21 vs. 32-33), fewer pectoral fin rays (10 vs. 12), fewer vertebra (38-41 vs. 44), fewer upper jaw teeth (6-8 vs. 9), fewer lateral line scales (50-59 vs. 69-73), and fewer scales between the lateral line and the pelvic fin origin (11-16 vs. 17-18).

*Pollimyrus ibalazambai* sp. nov. differs from *P. schreyeni* by having a dorsal fin origin slightly behind the level of the anal fin origin, whereas in *P. schreyeni* the dorsal fin origin lies clearly posterior of the anal fin origin. It further differs from *P. schreyeni* by having a longer dorsal fin (20.4-23.9% SL vs. 15.2-16.2% SL), a shorter anal fin (28.1-32.1% SL vs. 34.7-35.4% SL), a longer pectoral-pelvic distance (16.1-18.6% SL vs. 13.4-14.9% SL), and fewer anal fin rays (25-28 vs. 30-32).

*Pollimyrus ibalazambai* sp. nov. differs from *P. tumifrons* by having a shorter dorsal fin (20.4-23.9% SL vs. 29.3-30.6% SL), a longer anal fin (28.1-32.1% SL vs. 26.4-28.2% SL), a shorter head (20.9-22.8% SL vs. 24.0-26.6% SL), fewer dorsal fins (17-21 vs. 24-28), fewer pectoral fins (10 vs. 12), fewer lateral line scales (50-59 vs. 62-67), and fewer scales between the lateral line and the dorsal fin origin (11-14 vs. 16). In *P. ibalazambai* sp. nov. the mouth is positioned subterminal and the anterior nostril is positioned higher than the posterior one while in *P. tumifrons* it is below the eye and the nostrils are placed vice versa.

***Pollimyrus krameri* sp. nov.**
Figures 11 and 12, Tables 6 and 7
urn:lsid:zoobank.org:act:48E2A84F-625C-4891-A7E8-F6368805AE1E

*Pollimyrus krameri* sp. nov. *krameri* sp. nov. differs from *P. pedunculatus, P. ibalazambai* sp. nov*., P. adspersus, P. maculipinnis, P. nigripinnis-*complex*, P. osborni, C. plagiostoma,* and *P. schreyeni, P. tumifrons* by having a thicker caudal peduncle (23.4-23.4% BD vs. 12.7-20.2% BD), rounded (vs. pointed) pectoral fins, rounded (similar height of rays) anterior part of the dorsal and anal fins (vs. pointed) and by having a small chin (vs. no chin).

*Pollimyrus krameri* sp. nov. differs from *P. weyli* sp. nov. by having a shallower body (25.326.7% SL vs. 29.6-29.9% SL), a slenderer head (52.7-53.8% HL vs. 55.2-58.7% HL), a smaller eye (14.3-15.5% HL vs. 17.1-18.9% HL), a slenderer lower jaw (14.7-16.2% HL vs. 17.1-19.1% HL), a slenderer upper jaw (19.7-19.5% HL vs. 22.6-24.5% HL), a slenderer interorbital distance (31.7-33.1% HL vs. 37.2-40.9% HL), and fewer dorsal fin rays (15 vs. 17).

*Pollimyrus krameri* sp. nov. differs from *P. vanneeri* sp. nov. by having a longer predorsal length (66.8-67.9% SL vs. 56.4-65.0% SL), a shorter dorsal fin (17.3-17.5% SL vs. 22.1-25.1% SL), a shorter posterodorsal distance (36.8-37.0% SL vs. 39.9-45.3% SL), a deeper caudal peduncle (7.9% SL vs. 5.2-6.5% SL), a wider upper jaw (19.5-19.7% HL vs. 12.7-17.0% HL), a shorter snout (13.5% SL vs. 15.3-21.0% SL), fewer dorsal fin rays (15 vs. 21-24), fewer pectoral fin rays (9 vs. 10-12), fewer vertebrae (37-38 vs. 41-43), fewer lateral line scales (46-53 vs. 59-73), fewer caudal peduncle scales (13-14 vs 17-23), fewer scales between the lateral line and the dorsal fin origin (9 vs. 13-20), and fewer scales between the lateral line and the pelvic fin origin (11 vs. 14-20). *Pollimyrus krameri* sp. nov. has a rounded snout, rounded dorsal and anal fins, and a small chin, whereas *P. vanneeri* sp. nov. has a blocky snout, pointed dorsal and anal fins, and no obvious chin.

*Pollimyrus krameri* sp. nov. differs from *P. brevis* by having a shallower body (25.3-26.7% SL vs. 29.1-32.1% SL), a shorter dorsal fin (17.3-17.5% SL vs. 19.1-20.4% SL), a slenderer head (52.7-53.8% HL vs. 58.5-60.2% HL), a shorter snout-posterior side of eye distance (39.4-39.7% HL vs. 44.3-44.7% HL), a smaller eye (14.3-15.5% HL vs. 18.8-19.0% HL), a slenderer lower jaw (14.7-16.2% HL vs. 23.5-25.7% HL), a slenderer interorbital width (31.7-33.1% HL vs. 39.2-44.6% HL), fewer dorsal fin rays (15 vs. 18), fewer pectoral fin rays (9 vs. 10), and fewer vertebrae (37-38 vs. 39).

*Pollimyrus krameri* sp. nov. differs from *P. castelnaui* by having a shallower body (25.3-26.7% SL vs. 29.5-31.2% SL), a shorter snout-posterior side of eye distance (39.4-39.7% HL vs.42.3-46.1% HL), a shorter internasal distance (8.2-9.1% HL vs. 10.1% HL), a smaller eye (14.3-15.5% HL vs. 18.6-19.9% HL), a slenderer lower jaw (14.7-16.2% HL vs. 21.7-22.1% HL), a slenderer interorbital width (31.7-33.1% HL vs. 38.1-41.5% HL), and fewer dorsal fin rays (15 vs. 16-17).

*Pollimyrus krameri* sp. nov. differs from *P. cuandoensis* by having a shorter snout-posterior side of eye distance (39.4-39.7% HL vs. 40.5-45.3% HL), a smaller eye (14.3-15.5% HL vs. 15.5-21.9% HL).

*Pollimyrus krameri* sp. nov. differs from *P. fasciaticeps* by having shallower body (25.3-26.7% SL vs. 28.3% SL), a longer predorsal length (66.8-67.9% SL vs. 61.6% SL), a longer preanal length (60.6-62.2% SL vs. 58.9% SL), a longer dorsal fin (17.3-17.5% vs. 22.3% SL), a longer anal fin (23.1-24.5% SL vs. 27.7% SL), a shorter posterodorsal distance (36.8-37.0% SL vs. 43.5% SL), a shorter caudal peduncle (16.8-18.0% SL vs. 20.7% SL), a longer belly (19.5-19.7% SL vs. 17.4% SL), a slenderer head (52.7-53.8% HL vs. 59.7% HL), a shorter snout-posterior side of eye distance (39.4-39.7% HL vs. 50.0% HL), a shorter snout-centre of eye distance (31.8-32.2% HL vs. 37.0% HL), a smaller eye (14.3-15.5% HL vs. 26.8% HL), a wider upper jaw (19.5-19.7% HL vs. 14.2% HL), a slenderer interorbital width (31.7-33.1% HL vs. 41.4% HL), a shorter postorbital length (60.9-64.2% HL vs. 52.2% HL), fewer dorsal fin rays (15 vs. 17), fewer pectoral fins (9 vs. 10), fewer caudal peduncle scales (13-14 vs. 18), fewer scales between the lateral line and the anal fin origin (9-12 vs. 14), fewer scales between the lateral line and the dorsal fin origin (9 vs. 16), and fewer scales between the lateral line and the pectoral fin origin (11 vs. 14). *Pollimyrus krameri* sp. nov. has a rounded snout, rounded dorsal and anal fins, rounded pectoral fins, and a small chin, whereas *P. fasciaticeps* has a blocky snout, pointed dorsal and anal fins, pointed pectoral fin, and no obvious chin.

*Pollimyrus krameri* sp. nov. differs from *P. nigricans* by having a slenderer lower jaw (14.7-16.2% HL vs. 17.4-20.9% SL), fewer pectoral fin rays (9 vs. 10), and fewer caudal peduncle scales (13-14 vs. 17-20). *Pollimyrus krameri* sp. nov. has a rounded snout, rounded pectoral fins, and a small chin, whereas *P. nigricans* has a blocky snout, pointed pectoral fin, and no obvious chin.

*Pollimyrus krameri* sp. nov. differs from *P. isidori* by having a shallower body (25.3-26.7% SL vs. 31.9% SL), a longer predorsal length (66.8-67.9% SL vs. 63.8% SL), a shorter dorsal fin (17.3-17.5% SL vs. 22.5% SL), a shorter posterodorsal distance (36.8-37.0% SL vs. 42.8% SL), a shorter pectoral-pelvic distance (16.7-18.5% SL vs. 21.4% SL), a slenderer head (52.7-53.8% HL vs. 50.9% HL), a shorter snout-posterior side of eye distance (39.4-39.7% HL vs. 41.4% HL), a smaller eye (14.3-15.5% HL vs. 22.9% HL), a wider upper jaw (19.5-19.7% HL vs. 16.3% HL), a shorter snout (13.5% HL vs. 16.7% HL), a slenderer interorbital width (31.7-33.1% HL vs. 36.6% HL), a shorter postorbital length (60.9-64.2% HL vs. 57.3% HL), fewer dorsal fin rays (15 vs. 20), fewer pectoral fin rays (9 vs. 10), fewer caudal peduncle scales (13-14 vs. 16), fewer scales between the lateral line and the dorsal fin origin (9 vs. 14-15), and fewer scales between the lateral line and the pelvic fin origin (11 vs. 15-16). *Pollimyrus krameri* sp. nov. has a rounded snout, rounded pectoral fins, and a small chin, whereas *P. isidori* has a blocky snout, pointed pectoral fin, and no obvious chin.

*Pollimyrus krameri* sp. nov. differs from *P. petricolus* by having a longer predorsal length (66.8-67.9% SL vs. 60.3-64.0% SL), a shorter dorsal fin (17.3-17.5% SL vs. 21.4-24.9% SL), a shorter posterodorsal distance (36.8-37.0% SL vs. 38.7-41.8% SL), a slenderer caudal peduncle (23.4% BD vs. 25.4-28.8% BD), a wider head (52.7-53.8% HL vs. 45.3-47.6% HL), a shorter snout-posterior side of eye distance (39.4-39.7% HL vs. 42.4-45.9% HL), a smaller eye (14.3-15.5% HL vs. 21.6-23.7% HL), fewer dorsal fin rays (15 vs. 19-22), fewer pectoral fin rays (9 vs. 10), fewer vertebrae (37-38 vs. 41-43), fewer caudal peduncle scales (46-53 vs. 58-67), fewer scales between the lateral line and the dorsal fin origin (9 vs. 13-14), and fewer scales between the lateral line and the pelvic fin origin (11 vs. 14-15).

*Pollimyrus krameri* sp. nov. differs from *P. stappersii* by having a deeper caudal peduncle (7.9% SL vs. 5.9-6.1% SL), a shorter belly (19.5-19.7% SL vs. 20.8-21.6% SL), a longer head (24.8-25.2% SL vs. 23.7-23.8% SL), a shorter snout-posterior side of eye distance (39.4-39.7% HL vs. 44.3-44.5% HL), a shorter snout-centre of eye distance (31.8-32.2% HL vs. 34.3-35.1% HL), a smaller eye (14.3-15.5% HL vs. 16.9-18.0% HL), a slenderer lower jaw (14.7-16.2% HL vs. 17.9-18.3% HL), a slenderer upper jaw (19.5-19.7% HL vs. 16.6-18.2% HL), and a slenderer interorbital width (31.7-33.1% HL vs. 37.6-40.2% HL).

*Pollimyrus krameri* sp. nov. differs from *P. guttatus* by having a shallower body (25.3-26.7% SL vs. 30.7-32.1% SL), a longer predorsal length (66.8-67.9% SL vs. 65.0-66.4% SL), a shorter prepectoral distance (26.6% SL vs. 28.0-29.8% SL), a shorter dorsal fin (17.3-17.5% SL vs. 19.8-22.1% SL), a shorter pectoral fin (19.2-20.5% SL vs. 23.2-26.1% SL), a shorter posterodorsal length (36.8-37.0% SL vs. 38.9-42.0% SL), a shorter snout-posterior side of eye distance (39.4-39.7% HL vs.42.1-48.5% HL), a smaller eye (14.3-15.5% HL vs.22.1-27.3% HL), a wider upper jaw (19.5-19.7% HL vs. 13.9-15.6% HL), a longer postorbital length (60.9-64.2% HL vs. 55.8-58.7% HL), fewer dorsal fin rays (15 vs. 18-19), and fewer pectoral fin rays (9 vs. 10).

*Pollimyrus krameri* sp. nov. differs from *P. marianne* by having a longer dorsal fin (17.3-17.5% SL vs. 18.3-20.1% SL), a shorter posterodorsal length (36.8-37.0% SL vs. 39.8-43.7% SL), a longer head (24.8-25.2% SL vs. 21.5-23.3% SL), a slenderer head (52.7-53.8% HL vs. 58.4-61.4% HL), and a smaller eye (14.3-15.5% HL vs. 17.7-21.9% HL).

***Pollimyrus vanneeri* sp. nov.**Figure 14, Tables 6 and 7
urn:lsid:zoobank.org:act:5925507B-C01A-4319-8221-146366363783

*Pollimyrus vanneeri* sp. nov. differs from *P. pedunculatus, P. ibalazambai* sp. nov*., P. adspersus, P. nigripinnis-*complex*, P. osborni, C. plagiostoma,* and *P. schreyeni, P. tumifrons* by having a thicker caudal peduncle (20.3-24.4% BD vs. 12.7-20.2% BD) and rounded (vs. pointed) pectoral fins.

*Pollimyrus vanneeri* sp. nov. differs from *P. brevis* by having a shorter predorsal length (56.4-65.0% SL vs. 67.4-67.5% SL), a shorter preanal length (52.4-58.8% SL vs. 62.3-64.5% SL), a shorter prepelvic length (33.7-40.2% SL vs. 42.6-45.1% SL), a longer dorsal fin (22.1-25.1% SL vs. 19.1-20.4% SL), a thinner caudal peduncle (5.2-6.5% SL vs. 7.1-8.0% SL), a slenderer head (40.0-54.1% HL vs. 58.5-60.2% HL), a slenderer lower jaw (12.2-15.7% HL vs. 23.5-25.7% HL), a slenderer upper jaw (12.7-17.0% HL vs. 19.1-22.2% HL), a slenderer interorbital width (26.9-34.4% HL vs. 39.2-44.6% HL), a shorter postorbital length (52.7-59.9% HL vs. 61.3-63.5% HL), more dorsal fin rays (21-24 vs. 18), more vertebrae (41-43 vs. 39), more lateral line scales (59-73 vs. 40-46), more caudal peduncle scales (17-23 vs. 11-13), more scales between the lateral line and the dorsal fin origin (13-20 vs. 9), and more scales between the lateral line and the pelvic fin origin (14-20 vs. 10).

*Pollimyrus vanneeri* sp. nov. differs from *P. castelnaui* by having a shorter preanal length (52.4-58.8% SL vs. 60.1-62.4% SL), a longer dorsal fin (22.1-25.1% SL vs. 18.3-19.5% SL), a slenderer lower jaw (12.2-15.7% HL vs. 21.7-22.1% HL), a longer snout (15.3-21.0% HL vs. 11.9-13.4% HL), a slenderer interorbital width (26.9-34.4% HL vs. 38.1-41.5-% HL), more dorsal fin rays (21-24 vs. 16-17), more anal fin rays (24-27 vs. 22), more vertebrae (41-43 vs 38), more lateral line scales (17-23 vs. 12-13), and more scales between the lateral lines and the dorsal fin origin (13-20 vs. 10).

*Pollimyrus vanneeri* sp. nov. differs from *P. cuandoensis* by having a longer dorsal fin (22.1-25.1% SL vs. 16.0-18.9% SL), more dorsal fin rays (21-24 vs. 15-16), more anal fin rays (24-27 vs. 20-22), more vertebrae (41-43 vs. 37-38), more lateral line scales (59-73 vs. 46-51), more caudal peduncle scales (17-23 vs. 14-16), more scales between the lateral lines and the dorsal fin origin (13-20 vs. 9-12), and more scales between the lateral lines and the pectoral fin origin (14-20 vs. 11-13).

*Pollimyrus vanneeri* sp. nov. differs from *P. fasciaticeps* by having a slenderer head (40.0-54.1% HL vs. 59.7% HL), a shorter distance between the snout and the posterior side of the eye (40.4-45.4% HL vs. 50.0% HL), a smaller eye (13.7-21.6% HL vs. 26.8% HL), a longer snout length (15.3-21.0% HL vs. 13.2% HL), a slenderer interorbital width (26.9-34.4% HL vs. 41.4% HL), more dorsal fin rays (21-24 vs. 17), more vertebrae (41-43 vs. 37), and more lateral lines scales (59-73 vs 52).

*Pollimyrus vanneeri* sp. nov. differs from *P.* *guttatus* by having a shallower body (22.6-28.7% SL vs. 30.7-32.1% SL), a shorter preanal distance (52.4-58.8% SL vs. 62.2-65.8% SL), a shorter prepectoral distance (22.5-26.8% SL vs. 28.0-29.8% SL), a shorter prepelvic distance (33.7-40.2% SL vs. 43.7-45.7% SL), a shorter pectoral fin (17.7-22.7% SL vs. 23.2-26.1% SL), a smaller eye (13.7-21.6% HL vs. 22.1-27.3% HL), a longer snout (15.3-21.0% HL vs. 12.1-13.7% HL), more dorsal fin rays (21-24 vs. 18-19), more anal fin rays (24-27 vs. 22-23), more vertrebrae (41-43 vs. 37-38), more lateral line scales (59-73 vs. 43-52), more caudal peduncle scales (17-23 vs. 15-17), and more scales between the lateral line and dorsal fin origin (13-20 vs. 10-12).

*Pollimyrus vanneeri* sp. nov. differs from *P. isidori* by having a shallower body (22.6-28.7% SL vs. 31.9% SL), a shorter preanal length (52.4-58.8% SL vs. 62.7% SL), a shorter prepelvic length (33.7-40.2% SL vs. 44.2% SL), a shorter pectoral-pelvic distance (14.2-17.6% SL vs. 21.4% SL), a slenderer lower jaw (12.2-15.7% HL vs. 16.9% HL), a slenderer interorbital width (26.9-34.4% HL vs. 36.6% HL), more vertebrae (41-43 vs. 38), more lateral line scales (59-73 vs. 51), and more caudal peduncle scales (17-23 vs. 16).

*Pollimyrus vanneeri* sp. nov. differs from *P. maculipinnis* by having a shallower body (22.6-28.7% SL vs. 30.6-31.5% SL), a longer dorsal fin (22.1-25.1% SL vs. 19.0-19.5% SL), a smaller eye (13.7-21.6% HL vs. 27.1-27.9% HL), a slenderer lower jaw (12.2-15.7% HL vs. 17.3-18.2% HL), a slenderer interorbital width (26.9-34.4% HL vs. 39.0-41.2% HL), more dorsal fin rays (21-24 vs. 17); more vertebrae (41-43 vs. 38), and more caudal peduncle scales (17-23 vs. 15-16).

*Pollimyrus vanneeri* sp. nov. differs from *P. marianne* by having a longer dorsal fin (22.1-25.1% SL vs. 18.3-20.1% SL), a slenderer head (40.0-54.1% HL vs. 58.4-61.4% HL), a shallower caudal peduncle (20.3-24.2% BD vs. 24.4-29.3% BD), more dorsal fin rays (21-24 vs. 15-17), more anal fin rays (24-27 vs. 22-23), more vertebrae (41-43 vs. 38-39), more lateral line scales (59-73 vs. 48-54), more scales between the lateral line and the dorsal fin origin (13-20 vs. 9-11), and more scales between the lateral line and the pelvic fin origin (14-20 vs. 10-13).

*Pollimyrus vanneeri* sp. nov. differs from *P. nigricans* by having a shorter preanal length (52.4-58.8% SL vs. 59.2-62.5% SL), a longer dorsal fin (22.1-25.1% SL vs. 16.0-19.1% SL), a longer posterodorsal distance (39.9-45.3% SL vs. 35.5-38.7% SL), a slenderer lower jaw (12.2-15.7% HL vs. 17.4-20.9% HL), a shorter postorbital length (52.7-59.9% HL vs. 62.4-67.3% HL), more dorsal fin rays (21-24 vs. 14-17), more vertebrae (41-43 vs. 37-39), and more lateral line scales (59-73 vs. 48-55).

*Pollimyrus vanneeri* sp. nov. differs from *P. petricolus* by having a longer anal fin (24.0-30.2% SL vs. 21.3-24.3% SL), a smaller eye (13.7-21.6% HL vs. 22.4-23.7% HL), a longer snout (15.3-21.0% HL vs. 11.9-13.9% HL), and a shallower caudal peduncle (20.3-24.2% BD vs. 25.4-28.8% BD).

*Pollimyrus vanneeri* sp. nov. differs from *P stappersii* by having a shorter predorsal length (56.4-65.0% SL vs. 66.9-68.8% SL), a shorter preanal length (52.4-58.8% SL vs. 61.6-63.5% SL), a shorter prepelvic distance (33.7-40.2% SL vs. 41.8-43.5% SL), a longer dorsal fin (22.1-25.1% SL vs. 17.3-20.2% SL), a slenderer lower jaw (12.2-15.7% HL vs. 17.9-18.3% HL), a longer snout (15.3-21.0% HL vs. 14.0-12.3% HL), a slenderer interorbital width (26.9-34.4% HL vs. 37.6-40.2% HL), a shorter postorbital length (52.7-59.9% HL vs. 61.5-62.3% HL), more dorsal fin rays (21-24 vs. 16-18), more vertebrae (41-43 vs. 38-39), more lateral line scales (59-73 vs. 48-53), more caudal peduncle scales (17-23 vs. 13), and more scales between the lateral line and dorsal fin origin (13-20 vs. 10).

*Pollimyrus vanneeri* sp. nov. differs from *P. weyli* sp. nov. by having a shorter prepelvic distance (33.7-40.2% SL vs. 42.0-45.1% SL), a longer dorsal fin (22.1-25.1% SL vs. 18.1-18.8% SL), a shorter anal fin (24.0-30.2% SL vs. 20.2-22.5% SL), a longer posterodorsal distance (39.9-45.3% SL vs. 37.6-37.6% SL), a slenderer head (40.0-54.1% HL vs. 55.2-58.7% HL), a slenderer lower jaw width (12.2-15.7% HL vs. 17.7-19.1% HL), a slenderer upper jaw width (12.7-17.0% HL vs. 22.6-24.5% HL), a longer snout (15.3-21.0% HL vs. 13.7-14.7% HL), a slenderer interorbital width (26.9-34.4% HL vs. 37.2-40.9% HL), more dorsal fin rays (21-24 vs. 17), more anal fin rays (24-27 vs. 21-23), pectoral fin rays (10-12 vs. 9), and more vertebrae (41-43 vs. 38-39).

*Pollimyrus vanneeri* sp. nov. differs from *P. krameri* sp. nov. by having a longer dorsal fin (22.1-25.1% SL vs. 17.3-17.5% SL), a longer posterodorsal distance (39.9-45.3% SL vs. 36.8-37.0% SL), a slenderer upper jaw width (12.7-17.0% HL vs. 19.5-19.7% HL), a longer snout (17.9-15.3% HL vs. 13.5-13.5% HL), more dorsal fin rays (21-24 vs. 15), more pectoral fin rays (10-12 vs. 9), more vertebrae (41-43 vs. 37-38), more lateral line scales (59-73 vs. 46-53), more caudal peduncle scales (17-23 vs. 13-14), more scales between the lateral line and the dorsal fin origin (13-20 vs. 9), and more scales between the lateral line and the pelvic fin origin (14-20 vs. 11).

***Pollimyrus weyli* sp. nov.**Figures 15 and 16, Tables 6 and 7
urn:lsid:zoobank.org:act:FB535D7B-2446-4DB6-850A-E688C2E3F65D

*Pollimyrus weyli* sp. nov. differs from all other *Pollimyrus* species by having a wider upper jaw (22.6-24.5% HL vs. 12.7-22.2% HL).

*Pollimyrus weyli* sp. nov. further differs from *P. pedunculatus, P. ibalazambai* sp. nov*., P. adspersus, P. maculipinnis, P. nigripinnis-*complex*, P. osborni, C. plagiostoma,* and *P. schreyeni, P. tumifrons* by having a thicker caudal peduncle (23.4-23.8% BD vs. 12.7-20.2% BD), rounded (vs. pointed) pectoral fins, rounded (similar height of rays) anterior part of the dorsal and anal fins (vs. pointed) and by having a small chin (vs. no chin).

*Pollimyrus weyli* sp. nov. further differs from *P. krameri* sp. nov. by having a deeper body (29.6-29.9% SL vs. 25.3-26.7% SL), a wider head (55.2-58.7% HL vs. 52.7-53.8% HL), a larger eye (17.1-18.9% HL vs. 14.3-15.5% HL), a wider lower jaw (17.1-19.1% HL vs. 14.7-16.2% HL), a wider interorbital distance (37.2-40.9% HL vs. 31.7-33.1% HL), and more dorsal fin rays (17 vs. 15).

*Pollimyrus weyli* sp. nov. further differs from *P. vanneeri* sp. nov. by having a longer prepelvic distance (42.0-45.1% SL vs. 33.7-40.2% SL), a shorter dorsal fin (18.1-18.8% SL vs. 22.1-25.1% SL), a shorter anal fin (20.2-22.5% SL vs. 24.0-26.7% SL), a shorter posterodorsal distance (37.6% SL vs. 39.9-45.3% SL), a wider head (55.2-58.7% HL vs. 40.0-54.1% HL), a wider lower haw (17.1-19.1% HL vs. 12.2-15.7% HL), a wider interorbital distance (37.2-40.9% HL vs. 26.9-34.4% HL), fewer dorsal fin rays (17 vs. 21-24), fewer anal fin rays (21-23 vs. 24-27), fewer vertebrae (38-39 vs. 41-43), and fewer caudal peduncles scales (12-14 vs. 17-23). *Pollimyrus weyli* sp. nov. has a rounded snout, rounded dorsal and anal fins, and a small chin, whereas *P. vanneeri* sp. nov. has a blocky snout, pointed dorsal and anal fins, and no obvious chin.

*Pollimyrus weyli* sp. nov. further differs from *P. brevis* by having a shorter anal fin (20.2-22.5% SL vs. 24.3-24.4% SL), a shorter pectoral fin (18.1-19.6% SL vs. 20.5-22.9% SL), a longer caudal peduncle (16.7-18.0% SL vs. 15.2% SL), a slenderer lower jaw (17.1-19.1% HL vs. 23.5-25.7% HL), and fewer pectoral fin rays (9 vs. 10).

*Pollimyrus weyli* sp. nov. further differs from *P. castelnaui* by having a shorter anal fin (20.2-22.5% SL vs. 23.4-25.3% SL), a shorter pectoral fin (18.1-19.6% SL vs. 20.7-21.0% SL), a shorter belly (17.5-18.2% SL vs. 19.6-20.7% SL), a slenderer lower jaw (17.1-19.1% HL vs. 21.7-22.1% HL), and fewer pectoral fin rays (9 vs. 10).

*Pollimyrus weyli* sp. nov. further differs from *P. cuandoensis* by having a shorter pectoral-pelvic distance (18.5-20.3% SL vs. 16.1-17.9% SL), a longer snout (13.7-14.7% HL vs. 12.4-16.0% HL), and more dorsal fin rays (17 vs. 15-16).

*Pollimyrus weyli* sp. nov. further differs from *P. fasciaticeps* by having a longer predorsal distance (66.8-70.4% SL vs. 61.6% SL), a longer prepelvic distance (42.0-45.1% SL vs. 40.9% SL), a shorter dorsal fin (18.1-18.8% SL vs. 22.3% SL), a shorter anal fin (20.2-22.5% SL vs. 27.7% SL), a shorter pectoral fin (18.1-19.6% SL vs. 21.3% SL), a shorter posterodorsal distance (37.6% SL vs. 43.5% SL), a longer pectoral-pelvic distance (18.5-20.3% SL vs. 16.9% SL), a shorter caudal peduncle (16.7-18.0% SL vs. 20.7% SL), a shorter snout-posterior side of eye distance (39.6-43.5% HL vs. 50.0% HL), a smaller eye (17.1-18.9% HL vs. 26.8% HL), a longer postorbital length (60.5-62.5% HL vs. 52.2% HL), fewer pectoral rays (9 vs. 10), and fewer caudal peduncle scales (12-14 vs. 18). *Pollimyrus weyli* sp. nov. has a rounded snout, rounded dorsal and anal fins, rounded pectoral fins, and a small chin, whereas *P. fasciaticeps* has a blocky snout, pointed dorsal and anal fins, pointed pectoral fin, and no obvious chin.

*Pollimyrus weyli* sp. nov. further differs from *P. nigricans* by having a shorter pectoral fin (18.1-19.6% SL vs. 21.1-22.5% SL), fewer pectoral fins (9 vs. 10), and fewer caudal peduncle scales (12-14 vs. 17-20). *Pollimyrus weyli* sp. nov. has a rounded snout, rounded pectoral fins, and a small chin, whereas *P. nigricans* has a blocky snout, pointed pectoral fin, and no obvious chin.

*Pollimyrus bweyli* sp. nov. further differs from *P. isidori* by having a longer predorsal distance (66.8-70.4% SL vs. 63.8% SL), a shorter dorsal fin (18.1-18.8% SL vs. 22.5% SL), a longer anal fin (20.2-22.5% SL vs/ 26.8% SL), a shorter posterdorsal distance (37.6% SL vs. 42.8% SL), a wider head (55.2-58.7% HL vs. 50.9% HL), a smaller eye (17.1-18.9% HL vs. 22.9% HL), a shorter postorbital length (60.5-62.5% SL vs. 57.3% SL), fewer dorsal fin rays (17 vs. 20), fewer pectoral fins (9 vs. 10), and fewer caudal peduncle scales (12-14 vs. 16). *Pollimyrus weyli* sp. nov. has a rounded snout, rounded pectoral fins, and a small chin, whereas *P. isidori* has a blocky snout, pointed pectoral fin, and no obvious chin.

*Pollimyrus weyli* sp. nov. further differs from *P. petricolus* by having a deeper body (29.6-29.9% SL vs. 22.5-25.7% SL), a longer predorsal length (66.8-70.4% SL vs. 60.3-64.0% SL), a shorter dorsal fin (18.1-18.8% SL vs. 21.4-24.9% SL), a wider head (55.2-58.7% HL vs. 45.3-47.6% HL), a longer internasal distance (9.6-11.0% HL vs. 7.4-8.1% HL), a smaller eye (17.1-18.9% HL vs. 21.6-23.7% HL), a wider lower jaw (17.1-19.1% HL vs. 13.8-16.1% HL), a wider interorbital width (37.2-40.9% HL vs. 25.3-33.1% HL), a longer postorbital length (60.5-62.5% HL vs. 55.3-58.5% HL), fewer dorsal fin rays (17 vs. 19-22), fewer pectoral fin rays (9 vs. 10), fewer vertebrae (38-39 vs. 41-43), and fewer caudal peduncle scales (12-14 vs. 16-20).

*Pollimyrus weyli* sp. nov. further differs from *P. stappersii* by having a deeper body (29.6-29.9% SL vs. 27.1-27.9% SL), and a shorter belly (17.5-18.2% SL vs. 20.8-21.6% SL).

*Pollimyrus weyli* sp. nov. further differs from *P. guttatus* by having a shorter prepectoral distance (26.4-27.3% SL vs. 28.0-29.8% SL), a shorter anal fin (20.2-22.5% SL vs. 25.0-27.1% SL), a shorter pectoral fin (18.1-19.6% SL vs. 23.2-26.1% SL), a wider head (55.2-58.7% HL vs. 43.4-52.4% HL), a smaller eye (17.1-18.9% HL vs. 22.1-27.3% HL), a longre postorbital distance (60.5-62.5% HL vs. 55.8-58.7% HL), fewer dorsal fin rays (17 vs. 18-19), and fewer pectoral fin rays (9 vs. 10).

*Pollimyrus weyli* sp. nov. further differs from *P. marianne* by having a deeper body (29.6-29.9% SL vs. 23.7-27.0% SL), a longer predorsal distance (66.8-70.4% SL vs. 61.2-65.5% SL), shorter anal fin (20.2-22.5% SL vs. 23.6-26.5% SL), and a shorter posterodorsal distance (37.6% SL vs. 39.8-43.7% SL).

## PCA Loading tables and additional PCA plots

**Table S1.** Factor loadings of PC 1, PC 2 and PC 3 of PCA of 26 log-transformed measurements of the all analysed *Pollimyrus* specimens (n= 140).

|  | **PC 1** | **PC 2** | **PC 3** |
| --- | --- | --- | --- |
| **Standard length** | 0.20000 | -0.020463 | 0.176260 |
| **Predorsal length** | 0.18790 | 0.057879 | 0.088173 |
| **Preanal length** | 0.19179 | 0.059278 | 0.089492 |
| **Prepectoral length** | 0.18982 | 0.070934 | -0.100770 |
| **Prepelvic length** | 0.17976 | 0.094052 | 0.071862 |
| **Length of dorsal fin** | 0.25936 | -0.295380 | 0.082068 |
| **Length of anal fin** | 0.23525 | -0.356820 | 0.127820 |
| **Length of pectoral fin** | 0.19297 | -0.190190 | 0.056963 |
| **Length of pelvic fin** | 0.19706 | 0.025660 | -0.116800 |
| **Posterodorsal distance** | 0.21442 | -0.179860 | 0.251810 |
| **Pectoral-pelvic distance** | 0.18441 | 0.077551 | 0.259600 |
| **Caudal peduncle length** | 0.18754 | -0.044158 | 0.387190 |
| **Caudal peduncle depth** | 0.12575 | 0.437510 | 0.465660 |
| **Belly length** | 0.22299 | 0.013466 | 0.059546 |
| **Head width** | 0.17205 | 0.165370 | -0.020931 |
| **Head length** | 0.19565 | 0.093322 | -0.082077 |
| **Snout-posterior side of eye** | 0.20092 | -0.060832 | -0.219350 |
| **Snout centre of eye** | 0.20636 | 0.004193 | -0.226820 |
| **Internasal length** | 0.14668 | 0.146570 | -0.068421 |
| **Eye diameter** | 0.18461 | -0.383190 | -0.134900 |
| **Lower jaw width** | 0.18442 | 0.343620 | -0.262140 |
| **Upper jaw width** | 0.19846 | 0.274210 | -0.156300 |
| **Body depth** | 0.20449 | -0.162650 | -0.017357 |
| **Snout length** | 0.25679 | 0.039182 | -0.319840 |
| **Interorbital width** | 0.14287 | -0.053528 | -0.267220 |
| **Postorbital length** | 0.17943 | 0.251620 | 0.001580 |


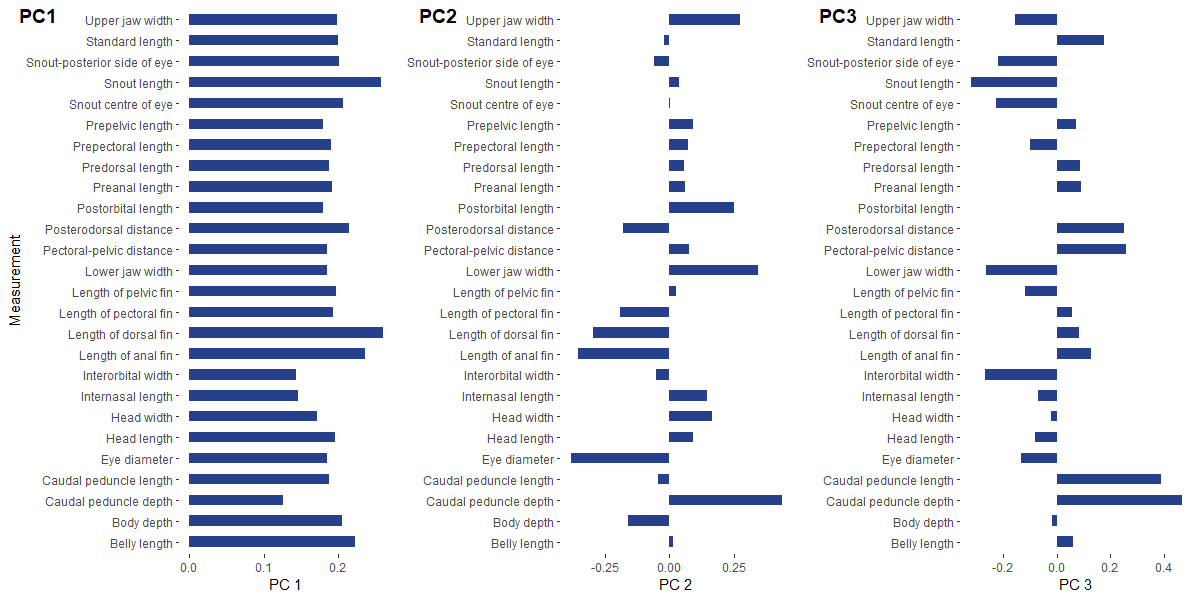


**Figure S20.** Factor loadings of PC 1, PC 2 and PC 3 of PCA of 26 log-transformed measurements of the all analysed *Pollimyrus* specimens (n= 140).


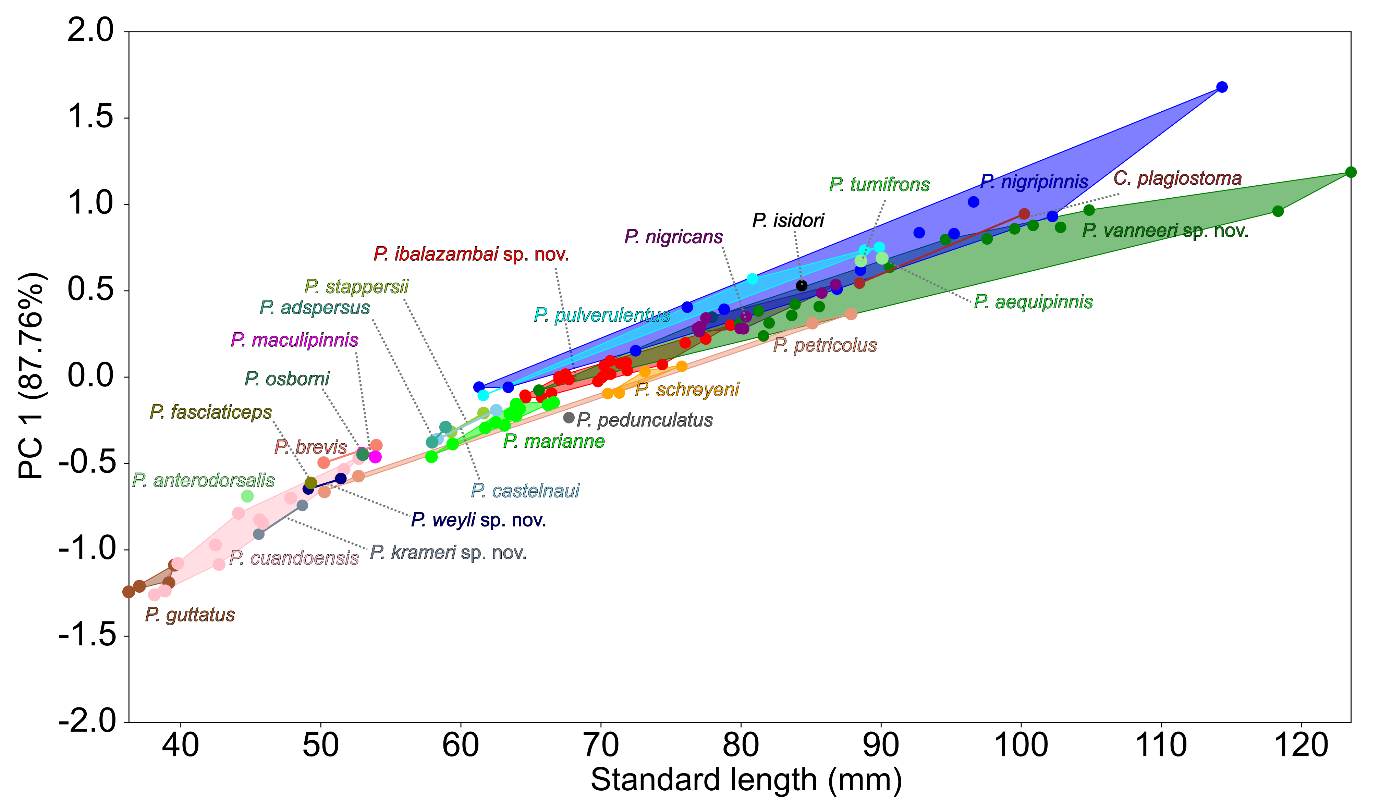


**Figure S21.** Differentiation of morphological groups with PC 1 against standard length (mm) for all *Pollimyrus* Taverne, 1971, specimens examined (n=140) (see Table 4), with explained variance between brackets for PC 1, on 26 log-transformed measurements for all *Pollimyrus* specimens examined (n=140) (see Table S1 for the loadings).

**Table S2.** Factor loadings of PC 1, PC 2 and PC 3 of PCA of 26 log-transformed measurements of the thick-tailed group (n= 41).

|  | **PC 1** | **PC 2** | **PC 3** |
| --- | --- | --- | --- |
| **Standard length** | 0.22229 | -0.084851 | -0.088951 |
| **Predorsal length** | 0.20572 | 0.012360 | -0.063470 |
| **Preanal length** | 0.20319 | -0.065513 | 0.038469 |
| **Prepectoral length** | 0.17560 | -0.024973 | -0.004427 |
| **Prepelvic length** | 0.19349 | -0.031945 | 0.040407 |
| **Length of dorsal fin** | 0.26169 | -0.366950 | 0.243420 |
| **Length of anal fin** | 0.23607 | -0.205700 | -0.035812 |
| **Length of pectoral fin** | 0.17315 | -0.130310 | -0.128510 |
| **Length of pelvic fin** | 0.18989 | -0.000583 | -0.225220 |
| **Posterodorsal distance** | 0.23655 | -0.204050 | -0.150070 |
| **Pectoral-pelvic distance** | 0.20509 | 0.007030 | 0.186030 |
| **Caudal peduncle length** | 0.20204 | -0.071766 | -0.438620 |
| **Caudal peduncle depth** | 0.17609 | 0.128680 | -0.223620 |
| **Belly length** | 0.23995 | -0.076356 | 0.065360 |
| **Head width** | 0.18428 | 0.156130 | -0.328060 |
| **Head length** | 0.17406 | -0.008506 | -0.002459 |
| **Snout-posterior side of eye** | 0.17125 | -0.060358 | 0.138210 |
| **Snout centre of eye** | 0.18110 | 0.080397 | 0.014218 |
| **Internasal length** | 0.14203 | 0.245810 | 0.020988 |
| **Eye diameter** | 0.16489 | -0.406200 | 0.405770 |
| **Lower jaw width** | 0.21119 | 0.460260 | 0.392730 |
| **Upper jaw width** | 0.23948 | 0.319850 | 0.031141 |
| **Body depth** | 0.16967 | 0.072923 | 0.180010 |
| **Snout length** | 0.16924 | 0.070439 | -0.155600 |
| **Interorbital width** | 0.12588 | 0.350720 | 0.199680 |
| **Postorbital length** | 0.17909 | 0.127050 | -0.098189 |


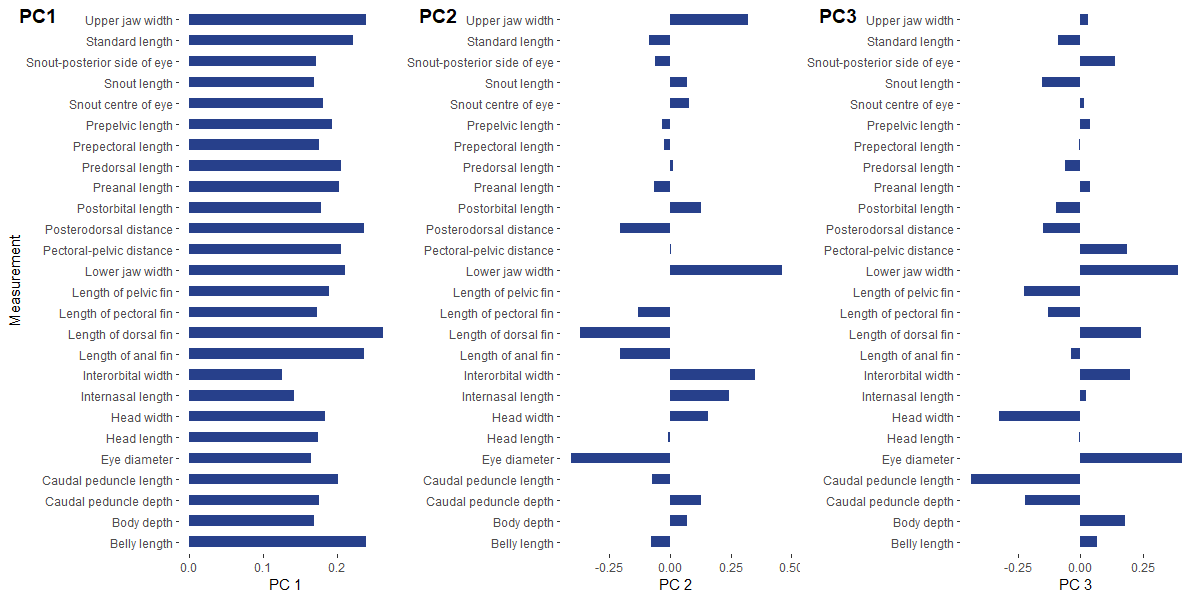


**Figure S22.** Factor loadings of PC 1, PC 2 and PC 3 of PCA of 26 log-transformed measurements of the thick-tailed group (n= 41).

**Table S3.** Factor loadings of PC 1, PC 2 and PC 3 of PCA of 12 meristics of the thick-tailed group (n= 41).

|  | **PC 1** | **PC 2** | **PC 3** |
| --- | --- | --- | --- |
| **Dorsal fin rays** | 0.384450 | 0.277760 | -0.100000 |
| **Anal fin rays** | 0.238170 | 0.537050 | -0.117290 |
| **Pectoral fin rays** | 0.260050 | 0.359160 | -0.287200 |
| **Pelvic fin rays** | -0.013266 | -0.168000 | -0.639700 |
| **Vertebra** | 0.363480 | 0.018219 | -0.030303 |
| **Teeth in upper jaw** | 0.025881 | 0.428450 | 0.389360 |
| **Teeth in lower jaw** | -0.033429 | -0.089584 | 0.459400 |
| **Lateral line scales** | 0.389400 | -0.149660 | 0.003644 |
| **Caudal peduncle scales** | 0.268310 | -0.329090 | -0.138700 |
| **Scales lateral line - anal fin origin** | 0.341050 | -0.024809 | 0.250110 |
| **Scales lateral line - dorsal fin origin** | 0.362220 | -0.315290 | 0.153110 |
| **Scales lateral line - pelvic fin origin** | 0.350170 | -0.233570 | 0.125390 |


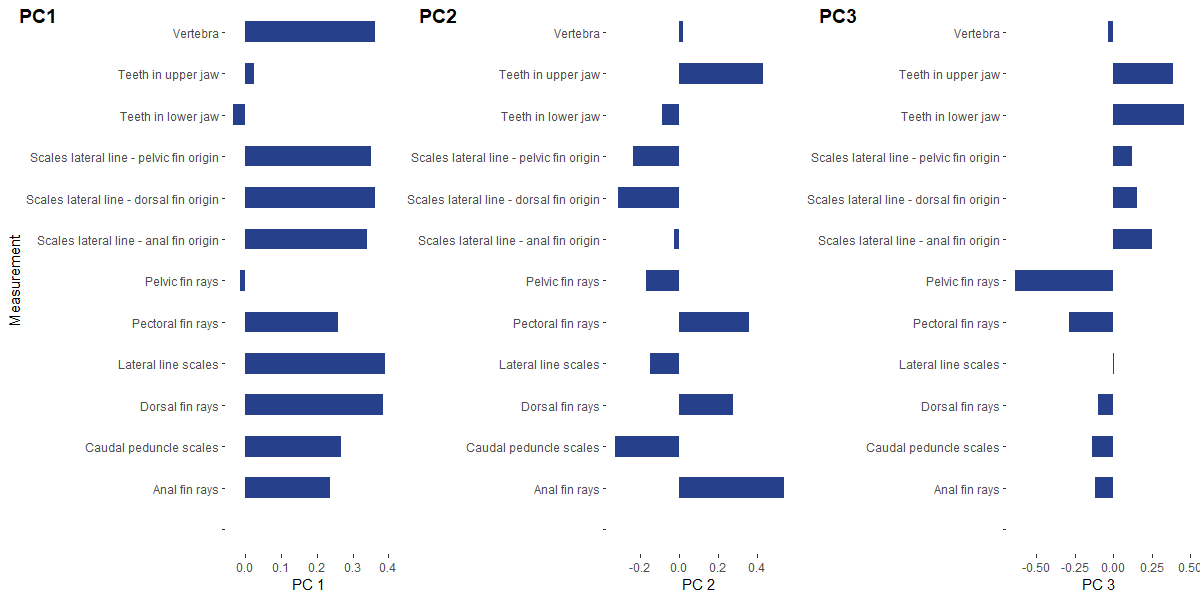


**Figure S23.** Factor loadings of PC 1, PC 2 and PC 3 of PCA of 12 meristics of the thick-tailed group (n= 41).

**Table S4.** Factor loadings of PC 1, PC 2 and PC 3 of PCA of 26 log-transformed measurements of the slender-tailed group (n= 49).

|  | **PC 1** | **PC 2** | **PC 3** |
| --- | --- | --- | --- |
| **Standard length** | 0.16960 | 0.142650 | 0.072053 |
| **Predorsal length** | 0.17556 | 0.009955 | 0.143800 |
| **Preanal length** | 0.19487 | 0.071097 | 0.112160 |
| **Prepectoral length** | 0.21533 | -0.156150 | 0.065381 |
| **Prepelvic length** | 0.18179 | 0.039653 | 0.097429 |
| **Length of dorsal fin** | 0.23563 | 0.381300 | -0.461780 |
| **Length of anal fin** | 0.15345 | 0.131420 | 0.159550 |
| **Length of pectoral fin** | 0.17469 | 0.153370 | 0.216590 |
| **Length of pelvic fin** | 0.22929 | -0.098611 | -0.136630 |
| **Posterodorsal distance** | 0.16181 | 0.323860 | -0.118970 |
| **Pectoral-pelvic distance** | 0.15043 | 0.306490 | 0.075317 |
| **Caudal peduncle length** | 0.13099 | 0.412150 | -0.017290 |
| **Caudal peduncle depth** | 0.12026 | 0.246760 | 0.250850 |
| **Belly length** | 0.23410 | 0.058256 | 0.226810 |
| **Head width** | 0.19707 | -0.078134 | 0.034688 |
| **Head length** | 0.20707 | -0.141020 | 0.018658 |
| **Snout-posterior side of eye** | 0.21230 | -0.218850 | 0.066145 |
| **Snout centre of eye** | 0.22780 | -0.202010 | 0.006879 |
| **Internasal length** | 0.17967 | -0.041102 | -0.022725 |
| **Eye diameter** | 0.13623 | -0.148730 | 0.336920 |
| **Lower jaw width** | 0.23299 | -0.263070 | 0.102610 |
| **Upper jaw width** | 0.24865 | -0.115290 | -0.023116 |
| **Body depth** | 0.18215 | 0.165650 | 0.003273 |
| **Snout length** | 0.29330 | -0.235560 | -0.604640 |
| **Interorbital width** | 0.14607 | -0.044314 | -0.078699 |
| **Postorbital length** | 0.19866 | -0.117330 | 0.078443 |


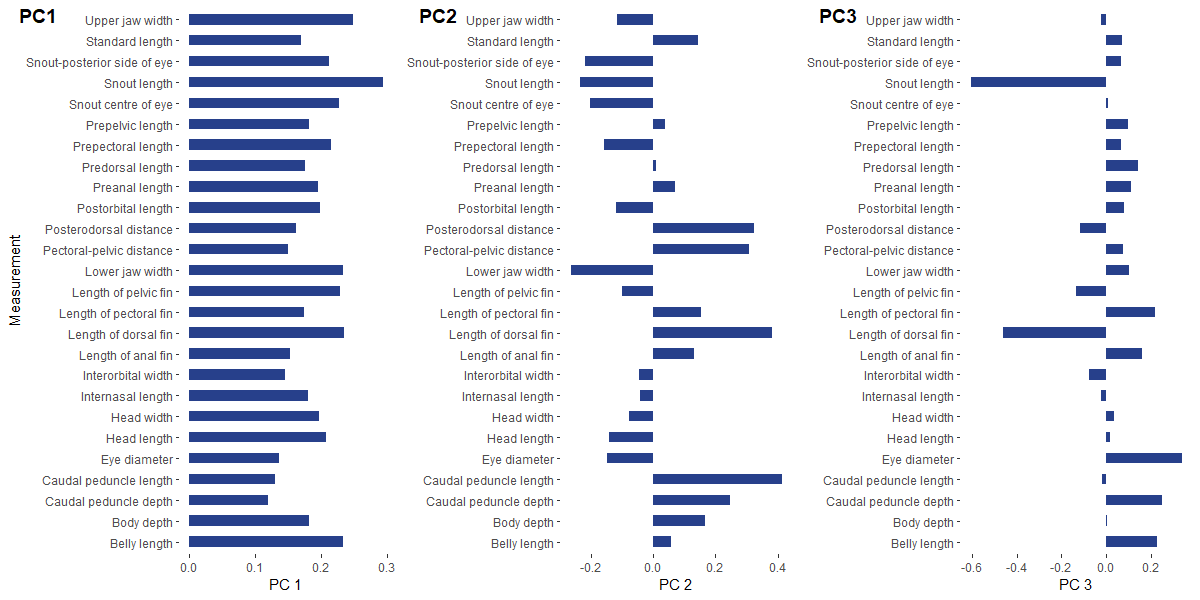


**Figure S24.** Factor loadings of PC 1, PC 2 and PC 3 of PCA of 26 log-transformed measurements of the slender-tailed group (n= 49).

**Table S5.** Factor loadings of PC 1, PC 2 and PC 3 of PCA of 12 meristics of the slender-tailed group (n= 49).

|  | **PC 1** | **PC 2** | **PC 3** |
| --- | --- | --- | --- |
| **Dorsal fin rays** | 0.421580 | 0.072447 | 0.323270 |
| **Anal fin rays** | 0.009798 | -0.328970 | 0.504330 |
| **Pectoral fin rays** | 0.421100 | -0.122610 | 0.120560 |
| **Vertebra** | 0.320980 | -0.379480 | 0.087766 |
| **Teeth in upper jaw** | 0.146660 | 0.483740 | 0.360760 |
| **Teeth in lower jaw** | 0.111840 | 0.488060 | 0.137000 |
| **Lateral line scales** | 0.411460 | -0.255980 | -0.051809 |
| **Caudal peduncle scales** | 0.121110 | 0.094512 | -0.542200 |
| **Scales lateral line - anal fin origin** | 0.345520 | -0.046519 | -0.184070 |
| **Scales lateral line - dorsal fin origin** | 0.346540 | 0.022948 | -0.372170 |
| **Scales lateral line - pelvic fin origin** | 0.290450 | 0.422060 | -0.030431 |


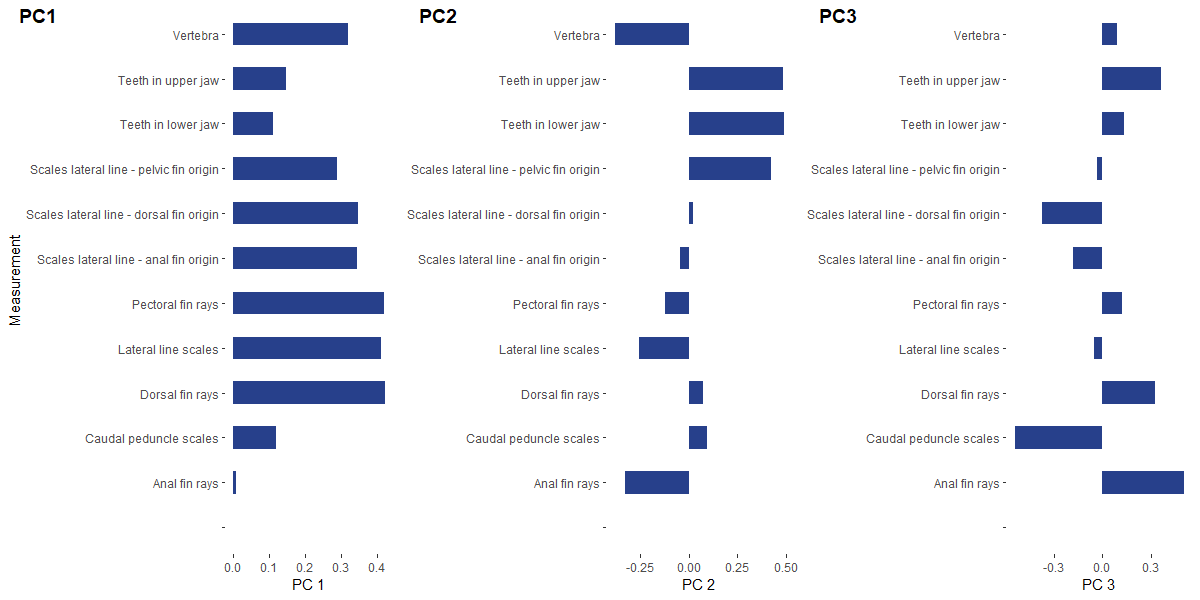


**Figure S25.** Factor loadings of PC 1, PC 2 and PC 3 of PCA of 12 meristics of the slender-tailed group (n= 49).


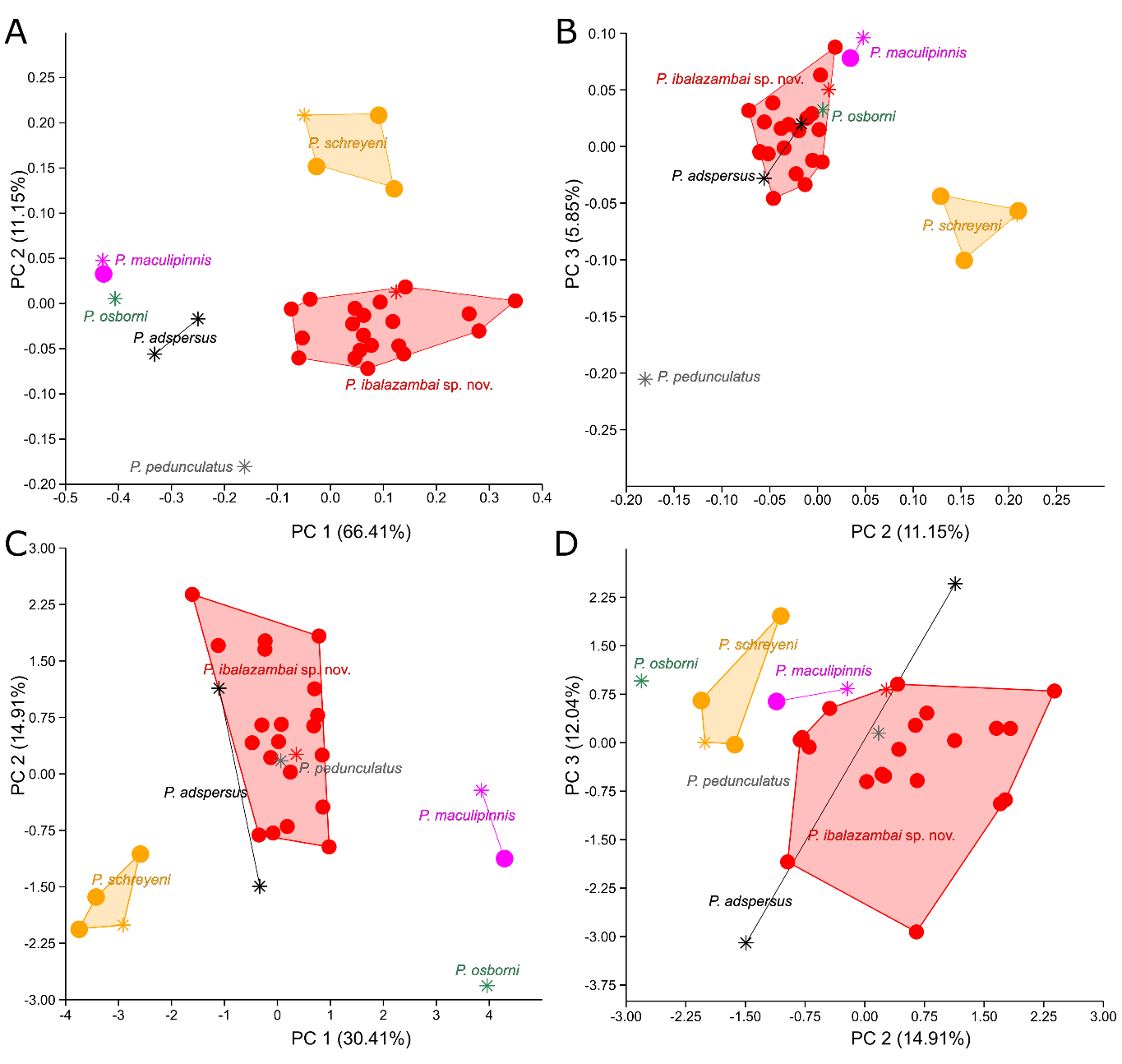


**Figure S26.** Plots of morphometric data of slender-tailed *Pollimyrus* Taverne, 1971 without the most distinct species. A. PC 1 (proxy for SL) against PC 2 for a PCA on 26 log-transformed measurements (n=31). B. PC 2 against PC 3 for a PCA on 26 log-transformed measurements (n=31). C. PC 1 against PC 2 for a PCA on 12 meristics (n=31). D. PC 2 against PC 3 for a PCA on 12 meristics (n=31). Stars indicate syntypes and holotypes, and circles paratypes. Explained variance is noted between brackets for each PCA.

**Table S6.** Factor loadings of PC 1, PC 2 and PC 3 of PCA of 26 log-transformed measurements of the *P.* *marianne* species-complex (n= 27).

|  | **PC 1** | **PC 2** | | **PC 3** |
| --- | --- | --- | --- | --- |
| **Standard length** | 0.21715 | -0.112060 | -0.005091 | |
| **Predorsal length** | 0.19640 | -0.053838 | -0.061262 | |
| **Preanal length** | 0.19878 | -0.061415 | -0.090729 | |
| **Prepectoral length** | 0.17323 | 0.004010 | 0.019115 | |
| **Prepelvic length** | 0.19305 | -0.046406 | -0.083072 | |
| **Length of dorsal fin** | 0.27159 | -0.211320 | -0.012708 | |
| **Length of anal fin** | 0.26847 | -0.234380 | -0.126790 | |
| **Length of pectoral fin** | 0.21166 | -0.165640 | -0.067226 | |
| **Length of pelvic fin** | 0.18248 | -0.122440 | 0.169720 | |
| **Posterodorsal distance** | 0.23994 | -0.174080 | 0.093684 | |
| **Pectoral-pelvic distance** | 0.20989 | -0.037078 | -0.276340 | |
| **Caudal peduncle length** | 0.20820 | -0.123400 | 0.265670 | |
| **Caudal peduncle depth** | 0.16883 | 0.191140 | 0.249920 | |
| **Belly length** | 0.21749 | -0.086724 | -0.244150 | |
| **Head width** | 0.17734 | -0.117700 | 0.311600 | |
| **Head length** | 0.15797 | 0.040343 | 0.060297 | |
| **Snout-posterior side of eye** | 0.15768 | 0.067405 | 0.007833 | |
| **Snout centre of eye** | 0.14513 | 0.080358 | 0.076514 | |
| **Internasal length** | 0.13673 | 0.287380 | 0.131950 | |
| **Eye diameter** | 0.17794 | -0.011511 | -0.061528 | |
| **Lower jaw width** | 0.20949 | 0.547860 | -0.419970 | |
| **Upper jaw width** | 0.25176 | 0.138220 | 0.075431 | |
| **Body depth** | 0.19263 | 0.025784 | -0.336570 | |
| **Snout length** | 0.14589 | 0.011419 | 0.402150 | |
| **Interorbital width** | 0.14329 | 0.554960 | 0.252710 | |
| **Postorbital length** | 0.15455 | 0.091592 | 0.067845 | |


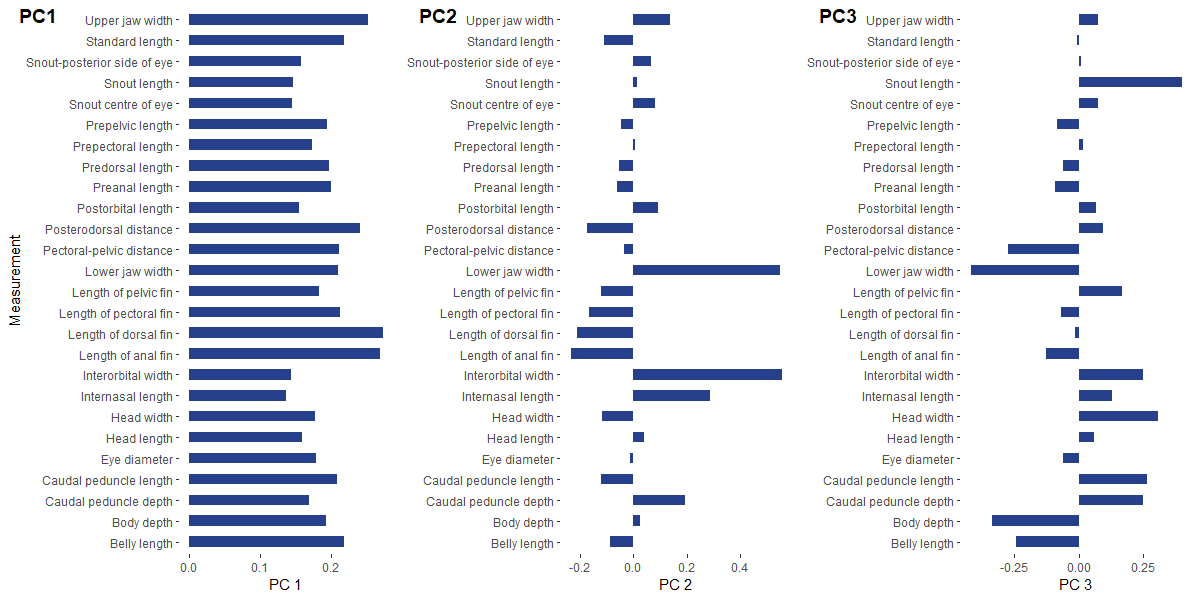


**Figure S27.** Factor loadings of PC 1, PC 2 and PC 3 of PCA of 26 log-transformed measurements of the *P.* *marianne* species-complex (n= 27).

**Table S7.** Factor loadings of PC 1, PC 2 and PC 3 of PCA of 26 log-transformed measurements of the *P. pulverulentus*-complex (n= 16).

|  | **PC 1** | **PC 2** | **PC 3** |
| --- | --- | --- | --- |
| **Standard length** | 0.18426 | 0.106410 | -0.095950 |
| **Predorsal length** | 0.18528 | 0.066382 | -0.043050 |
| **Preanal length** | 0.19455 | 0.147570 | -0.144310 |
| **Prepectoral length** | 0.17961 | 0.004719 | -0.038790 |
| **Prepelvic length** | 0.19201 | 0.062520 | -0.125970 |
| **Length of dorsal fin** | 0.20720 | -0.048930 | -0.441200 |
| **Length of anal fin** | 0.19002 | 0.028031 | -0.147980 |
| **Length of pectoral fin** | 0.15829 | 0.235090 | -0.007750 |
| **Length of pelvic fin** | 0.17749 | -0.072810 | -0.016710 |
| **Posterodorsal distance** | 0.18101 | 0.078615 | -0.138010 |
| **Pectoral-pelvic distance** | 0.22333 | 0.122190 | -0.039640 |
| **Caudal peduncle length** | 0.18296 | 0.267450 | 0.113780 |
| **Caudal peduncle depth** | 0.23390 | 0.148610 | 0.264220 |
| **Belly length** | 0.19633 | 0.394530 | -0.254920 |
| **Head width** | 0.20945 | -0.102940 | -0.006530 |
| **Head length** | 0.18591 | -0.039950 | 0.067057 |
| **Snout-posterior side of eye** | 0.19088 | -0.049690 | 0.042729 |
| **Snout centre of eye** | 0.21374 | -0.125120 | -0.082340 |
| **Internasal length** | 0.20567 | -0.250340 | 0.001861 |
| **Eye diameter** | 0.15253 | 0.216640 | 0.412220 |
| **Lower jaw width** | 0.21967 | 0.027204 | 0.567930 |
| **Upper jaw width** | 0.24818 | -0.220040 | 0.121700 |
| **Body depth** | 0.21493 | -0.060550 | -0.174360 |
| **Snout length** | 0.20354 | -0.635800 | 0.008107 |
| **Interorbital width** | 0.14128 | -0.165190 | 0.106830 |
| **Postorbital length** | 0.19034 | 0.012824 | 0.056409 |


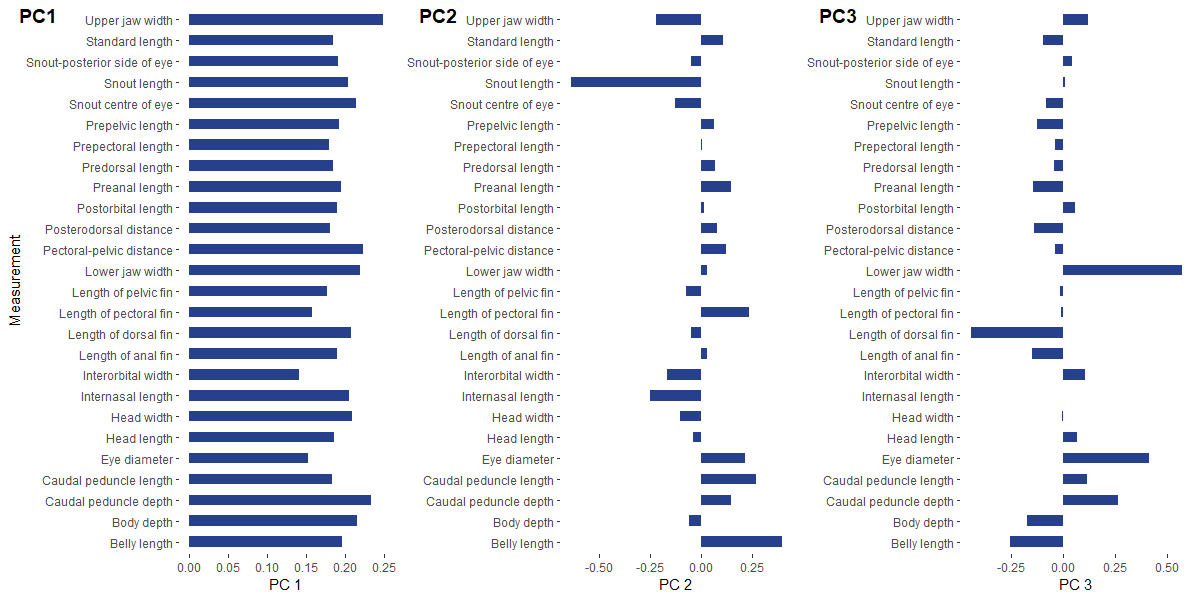


**Figure S28.** Factor loadings of PC 1, PC 2 and PC 3 of PCA of 26 log-transformed measurements of the *P. pulverulentus*-complex (n= 16).

**Table S8.** Factor loadings of PC 1, PC 2 and PC 3 of PCA of 12 meristics of the *P. pulverulentus*-complex (n= 16).

|  | **PC 1** | **PC 2** | **PC 3** |
| --- | --- | --- | --- |
| **Dorsal fin rays** | 0.451750 | -0.101240 | -0.179870 |
| **Anal fin rays** | 0.496220 | -0.191630 | -0.025540 |
| **Pectoral fin rays** | -0.025991 | 0.051930 | 0.060995 |
| **Vertebra** | 0.010920 | 0.500060 | -0.257370 |
| **Teeth in upper jaw** | 0.183590 | -0.276760 | 0.140360 |
| **Teeth in lower jaw** | -0.128260 | 0.275160 | 0.621520 |
| **Lateral line scales** | 0.287950 | 0.413770 | -0.287150 |
| **Caudal peduncle scales** | -0.219060 | 0.451420 | -0.189680 |
| **Scales lateral line - anal fin origin** | 0.306740 | 0.176640 | 0.550760 |
| **Scales lateral line - dorsal fin origin** | 0.309850 | 0.367530 | 0.236390 |
| **Scales lateral line - pelvic fin origin** | 0.421590 | 0.081829 | -0.115720 |


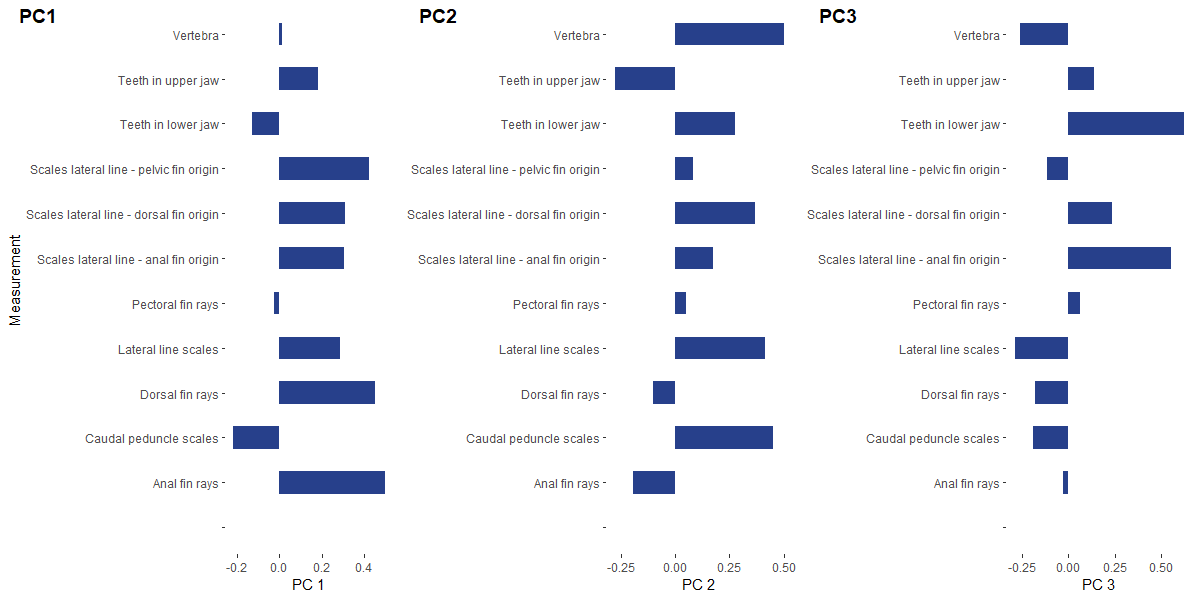


**Figure S29.** Factor loadings of PC 1, PC 2 and PC 3 of PCA of 12 meristics of the *P. pulverulentus*-complex (n= 16).

## Additional species data

**Table S9.** Measurements of the specimens within the *P. pulverulentus* complex examined*.*

| **Species** | ***P. nigripinnis*** | | | ***P. nigripinnis “Uéré”*** | ***P. nigripinnis “lac blue”*** | | | ***P. pulverulentus*** | | |
| --- | --- | --- | --- | --- | --- | --- | --- | --- | --- | --- |
|  | Syntype (n=6) | | | Syntype (n=1) | (n=5) | | | Syntype (n=4) | | |
|  | min | max | mean |  | min | max | mean | min | max | mean |
| **Standard length** | 72.5 | 102.2 | 87.3 | 61.3 | 63.4 | 114.3 | 88.6 | 61.6 | 89.9 | 80.3 |
| **Measurement in % SL** |  |  |  |  |  |  |  |  |  |  |
| **Total length** | 114.0% | 118.1% | 117.0% | 119.9% | 114.8% | 128.3% | 118.5% | 116.1% | 119.8% | 118.4% |
| **Body depth** | 28.4% | 30.7% | 29.1% | 28.2% | 30.7% | 35.8% | 32.4% | 30.0% | 33.4% | 31.8% |
| **Predorsal length** | 61.8% | 66.4% | 64.1% | 66.0% | 63.4% | 66.5% | 65.2% | 65.3% | 67.3% | 66.2% |
| **Preanal length** | 58.7% | 64.4% | 61.3% | 61.0% | 59.2% | 61.0% | 60.0% | 57.2% | 64.9% | 60.3% |
| **Prepectoral length** | 26.6% | 28.7% | 27.5% | 29.8% | 27.9% | 30.2% | 29.0% | 29.2% | 30.1% | 29.6% |
| **Prepelvic length** | 37.4% | 41.8% | 39.4% | 41.6% | 40.4% | 41.4% | 41.0% | 38.5% | 42.9% | 40.3% |
| **Length of dorsal fin** | 20.9% | 23.6% | 22.1% | 18.5% | 22.3% | 24.8% | 23.9% | 22.8% | 25.4% | 24.2% |
| **Length of anal fin** | 27.1% | 28.7% | 27.9% | 28.1% | 28.0% | 29.8% | 29.1% | 28.6% | 30.8% | 29.7% |
| **Length of pectoral fin** | 24.9% | 26.7% | 25.9% | 26.7% | 23.2% | 25.1% | 24.0% | 23.4% | 25.6% | 24.3% |
| **length of pelvic fin** | 9.9% | 11.1% | 10.7% | 11.8% | 10.8% | 12.4% | 11.6% | 11.3% | 12.3% | 11.8% |
| **Posterodorsal distance** | 38.8% | 42.1% | 40.2% | 38.7% | 39.4% | 41.7% | 40.5% | 40.1% | 42.9% | 41.2% |
| **Pectoral-pelvic distance** | 14.8% | 16.1% | 15.4% | 16.2% | 14.0% | 17.6% | 15.7% | 14.3% | 15.8% | 15.2% |
| **Caudal peduncle length** | 16.4% | 18.1% | 17.1% | 16.5% | 13.6% | 17.3% | 15.7% | 14.0% | 16.3% | 15.3% |
| **Caudal peduncle depth** | 4.5% | 5.1% | 4.7% | 5.1% | 3.9% | 5.4% | 4.7% | 4.1% | 4.7% | 4.5% |
| **Belly length** | 22.2% | 24.7% | 23.1% | 21.6% | 19.0% | 22.3% | 20.2% | 18.1% | 23.6% | 20.5% |
| **Head length** | 23.5% | 25.3% | 24.2% | 27.1% | 25.0% | 27.7% | 26.3% | 24.9% | 26.8% | 25.8% |
| **Measurement in % HL** |  |  |  |  |  |  |  |  |  |  |
| **Head width** | 47.5% | 51.3% | 49.1% | 49.3% | 47.6% | 54.1% | 52.0% | 47.7% | 54.7% | 50.8% |
| **Snout-posterior side of eye** | 44.7% | 50.8% | 47.5% | 48.7% | 44.7% | 53.8% | 47.9% | 46.8% | 50.0% | 48.0% |
| **Snout-centre of eye** | 32.4% | 37.6% | 36.0% | 32.6% | 35.4% | 43.8% | 38.3% | 36.7% | 39.8% | 37.9% |
| **Internasal length** | 6.8% | 8.2% | 7.5% | 7.8% | 8.5% | 9.1% | 8.7% | 7.7% | 8.3% | 8.2% |
| **Eye diameter** | 22.8% | 24.2% | 23.5% | 25.3% | 17.5% | 24.9% | 19.5% | 22.2% | 24.5% | 23.2% |
| **Lower jaw width** | 15.9% | 18.6% | 17.1% | 18.8% | 14.2% | 20.8% | 16.8% | 15.6% | 17.9% | 16.4% |
| **Upper jaw width** | 15.0% | 17.7% | 16.3% | 17.2% | 15.5% | 21.5% | 18.7% | 16.2% | 19.5% | 18.1% |
| **Snout length** | 9.8% | 17.4% | 14.1% | 15.4% | 18.2% | 19.9% | 18.8% | 16.4% | 16.7% | 16.5% |
| **Interorbital width** | 31.1% | 35.7% | 33.3% | 38.7% | 29.8% | 38.0% | 34.7% | 31.1% | 39.1% | 35.6% |
| **Postorbital length** | 54.7% | 57.4% | 56.1% | 56.0% | 54.4% | 58.0% | 55.5% | 49.7% | 56.2% | 53.2% |
| **Caudal peduncle depth (% BD)** | 15.3% | 16.9% | 16.1% | 18.3% | 12.7% | 17.4% | 14.5% | 13.7% | 14.4% | 14.1% |
| **Caudal peduncle depth (% CPL)** | 25.0% | 30.38% | 27.6% | 31.2% | 25.5% | 32.0% | 30.0% | 26.6% | 32.5% | 29.5% |
| **Anal fin length (% LD)** | 121.4% | 130.3% | 126.5% | 151.8% | 118.0% | 129.7% | 122.0% | 120.0% | 126.7% | 122.9% |

**Table S10.** Meristics of the specimens within the *P. pulverulentus* complex examined*.*

| **Species** | ***P. nigripinnis*** | | ***P. nigripinnis “Uéré”*** | ***P. nigripinnis “lac blue”*** | | ***P. pulverulentus*** | | |
| --- | --- | --- | --- | --- | --- | --- | --- | --- |
|  | Syntype (n=6) | | Syntype (n=1) | (n=5) | | Syntype (n=4) | | |
|  | min | max |  | min | max | min | max |  |
| **Dorsal fin rays** | 19 | 21 | 18 | 19 | 23 | 22 | 24 |  |
| **Anal fin rays** | 25 | 26 | 25 | 25 | 27 | 27 | 28 |  |
| **Pectoral fin rays** | 10 | 10 | 10 | 9 | 10 | 10 | 10 |  |
| **Pelvic fin rays** | 6 | 6 | 6 | 6 | 6 | 6 | 6 |  |
| **Vertebra** | 38 | 40 | 37 | 37 | 39 | 38 | 38 |  |
| **Teeth in upper jaw** | 8 | 9 | 10 | 9 | 10 | 9 | 10 |  |
| **Teeth in lower jaw** | 10 | 11 | ? | 9 | 11 | 9 | 10 |  |
| **Lateral line scales** | 47 | 54 | 47 | 42 | 52 | 49 | 54 |  |
| **Caudal peduncle scales** | 12 | 16 | 13 | 11 | 12 | 11 | 14 |  |
| **Scales lateral line - anal fin origin** | 10 | 14 | 10 | 12 | 13 | 11 | 14 |  |
| **Scales lateral line - dorsal fin origin** | 11 | 13 | 11 | 10 | 12 | 11 | 13 |  |
| **Scales lateral line - pelvic fin origin** | 13 | 17 | 16 | 12 | 18 | 13 | 18 |  |

**Table S11.** Measurements of the type specimens of *P. cuandoensis,* now a junior synonym of *P. marianne*, examined*.*

| **Species** | ***P. cuandoensis*** | | | |
| --- | --- | --- | --- | --- |
|  | Holotype | Paratype (n=11) | | All |
|  |  | min | max | mean |
| **Standard length** | 44.1 | 38.1 | 52.7 | 45.1 |
| **Measurement in % SL** |  |  |  |  |
| **Total length** | 117.0% | 105.6% | 117.0% | 112.6% |
| **Body depth** | 28.5% | 25.2% | 29.8% | 27.3% |
| **Predorsal length** | 66.7% | 64.6% | 69.2% | 66.3% |
| **Preanal length** | 61.4% | 58.9% | 62.4% | 60.5% |
| **Prepectoral length** | 29.8% | 26.3% | 29.8% | 27.7% |
| **Prepelvic length** | 43.4% | 40.3% | 43.9% | 42.1% |
| **Length of dorsal fin** | 18.2% | 16.0% | 18.9% | 17.1% |
| **Length of anal fin** | 22.2% | 19.9% | 24.1% | 22.5% |
| **Length of pectoral fin** | 21.8% | 19.4% | 21.9% | 21.1% |
| **length of pelvic fin** | 11.5% | 9.8% | 11.9% | 11.2% |
| **Posterodorsal distance** | 40.0% | 36.4% | 40.5% | 39.1% |
| **Pectoral-pelvic distance** | 17.9% | 16.1% | 17.9% | 16.7% |
| **Caudal peduncle length** | 18.0% | 17.1% | 20.8% | 18.7% |
| **Caudal peduncle depth** | 8.8% | 6.6% | 8.8% | 7.7% |
| **Belly length** | 18.2% | 17.3% | 20.1% | 18.6% |
| **Head length** | 26.8% | 23.3% | 26.8% | 25.1% |
| **Measurement in % HL** |  |  |  |  |
| **Head width** | 55.8% | 47.8% | 63.4% | 55.8% |
| **Snout-posterior side of eye** | 45.3% | 40.5% | 45.3% | 42.8% |
| **Snout-centre of eye** | 36.0% | 31.3% | 36.8% | 34.3% |
| **Internasal length** | 10.7% | 8.6% | 11.0% | 9.9% |
| **Eye diameter** | 18.7% | 15.5% | 21.9% | 18.9% |
| **Lower jaw width** | 20.6% | 13.6% | 21.1% | 17.1% |
| **Upper jaw width** | 15.5% | 13.3% | 20.2% | 15.9% |
| **Snout length** | 12.4% | 12.4% | 16.0% | 13.9% |
| **Interorbital width** | 47.9% | 26.1% | 50.1% | 38.6% |
| **Postorbital length** | 62.9% | 58.0% | 63.5% | 61.6% |
| **Caudal peduncle depth (% BD)** | 30.8% | 23.1% | 31.2% | 28.3% |
| **Caudal peduncle depth (% CPL)** | 48.6% | 37.8% | 48.6% | 41.4% |
| **Anal fin length (% LD)** | 122.3% | 122.9% | 140.7% | 131.8% |

**Table S12.** Meristics of the type specimens of *P. cuandoensis,* now a junior synonym of *P. marianne*, examined*.*

| **Species** | ***P. cuandoensis*** | | |
| --- | --- | --- | --- |
|  | Holotype | Paratype (n=11) | |
|  |  | min | max |
| **Dorsal fin rays** | 16 | 15 | 16 |
| **Anal fin rays** | 22 | 20 | 22 |
| **Pectoral fin rays** | 9 | 9 | 10 |
| **Pelvic fin rays** | 6 | 6 | 6 |
| **Vertebra** | 38 | 37 | 37 |
| **Teeth in upper jaw** | 7 | 6 | 7 |
| **Teeth in lower jaw** | 8 | 7 | 9 |
| **Lateral line scales** | 48 | 46 | 51 |
| **Caudal peduncle scales** | 14 | 14 | 16 |
| **Scales lateral line - anal fin origin** | 11 | 10 | 13 |
| **Scales lateral line - dorsal fin origin** | 10 | 9 | 12 |
| **Scales lateral line - pelvic fin origin** | 12 | 11 | 13 |

**Table S13.** Measurements of non-*Pollimyrus* species examined, *Paramormyrops eburneensis* and *Cyphomyrus. petherici.*

| **Species** | ***P. eburneensis*** | | | | ***C. petherici*** | | |
| --- | --- | --- | --- | --- | --- | --- | --- |
|  | Holotype | Paratype (n=7) | | All | Syntype (n=3) | | |
|  |  | min | max | mean | min | max | mean |
| **Standard length** | 72.9 | 48.1 | 75.9 | 65.2 | 127.5 | 194.2 | 165.7 |
| **Measurement in % SL** |  |  |  |  |  |  |  |
| **Total length** | 109.8% | 109.8% | 116.3% | 113.1% | 116.3% | 118.3% | 117.3% |
| **Body depth** | 18.7% | 18.7% | 21.3% | 20.0% | 28.5% | 32.0% | 30.5% |
| **Predorsal length** | 67.0% | 67.0% | 70.6% | 69.4% | 56.1% | 56.8% | 56.4% |
| **Preanal length** | 60.3% | 59.9% | 65.5% | 62.7% | 61.2% | 63.3% | 62.3% |
| **Prepectoral length** | 24.3% | 24.3% | 26.7% | 25.7% | 22.5% | 23.0% | 22.6% |
| **Prepelvic length** | 40.0% | 39.6% | 43.5% | 41.5% | 37.2% | 40.4% | 38.6% |
| **Length of dorsal fin** | 15.2% | 15.2% | 17.7% | 16.4% | 31.2% | 33.8% | 32.5% |
| **Length of anal fin** | 21.0% | 21.0% | 25.5% | 23.0% | 19.7% | 21.1% | 20.2% |
| **Length of pectoral fin** | 14.7% | 14.7% | 20.0% | 16.8% | 20.3% | 20.8% | 20.5% |
| **length of pelvic fin** | 10.0% | 10.0% | 12.4% | 11.2% | 11.1% | 12.2% | 11.8% |
| **Posterodorsal distance** | 32.4% | 31.4% | 36.0% | 33.3% | 49.5% | 52.1% | 50.9% |
| **Pectoral-pelvic distance** | 17.8% | 17.0% | 18.4% | 17.7% | 16.4% | 19.4% | 18.2% |
| **Caudal peduncle length** | 15.0% | 12.7% | 17.2% | 14.8% | 20.3% | 21.1% | 20.8% |
| **Caudal peduncle depth** | 6.5% | 6.3% | 7.0% | 6.7% | 6.5% | 7.5% | 7.1% |
| **Belly length** | 20.3% | 19.1% | 23.3% | 21.0% | 22.8% | 24.5% | 23.8% |
| **Head length** | 21.0% | 21.0% | 23.9% | 23.0% | 19.9% | 21.2% | 20.6% |
| **Measurement in % HL** |  |  |  |  |  |  |  |
| **Head width** | 53.1% | 47.9% | 69.4% | 54.0% | 50.6% | 55.9% | 52.7% |
| **Snout-posterior side of eye** | 43.0% | 39.0% | 48.7% | 41.7% | 50.3% | 52.7% | 51.2% |
| **Snout-centre of eye** | 35.1% | 33.7% | 36.4% | 34.7% | 39.4% | 41.8% | 40.4% |
| **Internasal length** | 8.6% | 7.3% | 9.4% | 8.5% | 5.6% | 7.1% | 6.6% |
| **Eye diameter** | 13.4% | 10.5% | 14.6% | 12.4% | 18.2% | 25.6% | 22.3% |
| **Lower jaw width** | 13.2% | 13.2% | 24.6% | 18.1% | 12.6% | 14.7% | 13.8% |
| **Upper jaw width** | 14.2% | 12.1% | 16.2% | 14.7% | 9.7% | 15.6% | 13.1% |
| **Snout length** | 14.6% | 10.5% | 15.4% | 14.1% | 16.3% | 16.9% | 16.5% |
| **Interorbital width** | 34.9% | 31.9% | 40.0% | 36.3% | 32.6% | 34.8% | 33.6% |
| **Postorbital length** | 60.5% | 60.5% | 65.5% | 62.9% | 51.5% | 51.9% | 51.8% |
| **Caudal peduncle depth (% BD)** | 34.9% | 29.4% | 36.5% | 33.3% | 22.7% | 23.7% | 23.3% |
| **Caudal peduncle depth (% CPL)** | 43.6% | 39.3% | 54.7% | 45.2% | 30.8% | 37.0% | 34.2% |
| **Anal fin length (% LD)** | 137.7% | 129.5% | 155.0% | 140.3% | 60.7% | 63.6% | 62.2% |

**Table S14.** Meristics of non-*Pollimyrus* species examined, *Paramormyrops eburneensis* and *Cyphomyrus petherici.*

| **Species** | ***P. eburneensis*** | | | ***C. petherici*** | |
| --- | --- | --- | --- | --- | --- |
|  | Holotype | Paratype (n=7) | | Syntype (n=3) | |
|  |  | min | max | min | max |
| **Dorsal fin rays** | 15 | 15 | 16 | 34 | 35 |
| **Anal fin rays** | 20 | 19 | 21 | 24 | 26 |
| **Pectoral fin rays** | 9 | 9 | 9 | 10 | 10 |
| **Pelvic fin rays** | 6 | 6 | 6 | 6 | 6 |
| **Vertebra** | 42 | 42 | 43 | 41 | 41 |
| **Teeth in upper jaw** | 7 | 7 | 8 | 5 | 6 |
| **Teeth in lower jaw** | 7 | 7 | 8 | 5 | 7 |
| **Lateral line scales** | 63 | 57 | 64 | 65 | 69 |
| **Caudal peduncle scales** | 17 | 14 | 19 | 12 | 18 |
| **Scales lateral line - anal fin origin** | 10 | 9 | 11 | 9 | 15 |
| **Scales lateral line - dorsal fin origin** | 10 | 9 | 11 | 20 | 24 |
| **Scales lateral line - pelvic fin origin** | 12 | 11 | 14 | 12 | 16 |
